# Biomarkers and therapeutic strategies targeting microglia in neurodegenerative diseases: current status and future directions

**DOI:** 10.1186/s13024-025-00867-4

**Published:** 2025-07-10

**Authors:** Min-Young Noh, Hyuk Sung Kwon, Min-Soo Kwon, Minyeop Nahm, Hee Kyung Jin, Jae-sung Bae, Seung Hyun Kim

**Affiliations:** 1 Department of Neurology, College of Medicine, Hanyang University, 222, Wangsimni-Ro, Seongdong-Gu, Seoul, 04763 Republic of Korea; 2 Department of Neurology, Hanyang University Guri Hospital, Hanyang University College of Medicine, 153, Gyeongchun-Ro, Guri-Si, Gyeonggi-Do, 11923 Republic of Korea; 3 Department of Pharmacology, Research Institute of Basic Medical Science, School of Medicine, CHA University, CHA Bio Complex, 335 Pangyo, Gyeonggi-do, 13488 Republic of Korea; 4 Dementia Research Group, Korea Brain Research Institute, Daegu, 41062 Republic of Korea; 5 Department of Laboratory Animal Medicine, College of Veterinary Medicine, Kyungpook National University, Daegu, 41566 Republic of Korea; 6 Department of Physiology, School of Medicine, Kyungpook National University, Daegu, 41944 Republic of Korea; 7 Cell Therapy Center, Hanyang University Medical Center, Wangsimniro 222-1, Seoul, 04763 Republic of Korea

**Keywords:** Microglia-targeting therapy, Microglial dysfunction, Companion diagnostics, Neurodegenerative diseases, AD, PD, ALS, FTD, Precision medicine

## Abstract

Recent advances in our understanding of non-cell-autonomous mechanisms in neurodegenerative diseases (NDDs) have highlighted microglial dysfunction as a core driver of disease progression. Conditions such as Alzheimer’s disease (AD), amyotrophic lateral sclerosis (ALS), Parkinson’s disease (PD), and frontotemporal dementia (FTD) share features of impaired microglial phagocytosis, chronic neuroinflammation, and metabolic dysregulation. These insights have prompted new therapeutic strategies targeting microglial function and emphasized the need for reliable biomarkers to monitor disease progression and treatment response. Well-established therapeutic targets, such as triggering receptor expressed on myeloid cells 2 (TREM2), progranulin (PGRN), and sortilin (SORT1), along with emerging candidates including LILRB4, P2Y6R, TAM receptors, and neuroinflammation-related markers, are discussed alongside novel blood, cerebrospinal fluid (CSF), and imaging biomarkers. Despite notable progress, many of these biomarkers remain restricted to preclinical studies and face translational challenges due to species-specific differences, lack of standardization, and clinical heterogeneity. Emerging technologies—including single-cell omics, spatial transcriptomics, and artificial intelligence (AI)-driven integration of multimodal data—offer new opportunities to align biomarker profiles with evolving disease states and improve patient stratification. Building on the model of companion diagnostics (CDx) in oncology, integrating multimodal biomarker strategies holds promise for guiding personalized interventions, improving clinical outcomes, and deepening our mechanistic understanding of microglial contributions across the neurodegenerative spectrum.

**Supplementary Information:**

The online version contains supplementary material available at 10.1186/s13024-025-00867-4.

## Introduction

Recent advances have highlighted the crucial role of non-neuronal cells in maintaining the central nervous system’s (CNS) homeostasis. Once considered merely supportive, glial cells are now known to actively contribute to synaptic remodeling, CNS immunity, and the integrity of the blood–brain barrier (BBB) [1–3]. Their involvement in pathological conditions has also come into focus, with non-cell-autonomous mechanisms driving neurodegeneration through neuroinflammation pathways, altered glial function, and disrupted metabolic support [4]. This dynamic interplay between degenerating neurons and glial cells creates a feedback loop that exacerbates neurodegeneration and accelerates disease progression. Understanding these mechanisms has revealed new therapeutic opportunities, indicating that targeting glial cells and modulating the neuronal microenvironment may offer promising strategies for treating neurodegenerative diseases (NDDs) such as Alzheimer’s disease (AD), amyotrophic lateral sclerosis (ALS), Frontotemporal dementia (FTD), and Parkinson’s disease (PD).

Microglia, the innate immune cells of the CNS, serve as the first line of defense by constantly surveilling their environment. They play crucial roles in synaptic pruning, damage repair, homeostasis maintenance, phagocytosis, and communication with other glial and neuronal cells [5]. The concept of microglial “activation” has evolved to better capture their complex responses to external insults and internal pathological stimuli. The term “reactive microglia” is now preferred over “activated microglia” to emphasize the nuanced and heterogeneous nature of their responses [6].

Historically, microglial activation was linked to neuroinflammation. However, current evidence shows that their responses are highly dynamic and cannot be captured by the binary M1/M2 polarization model [7–9]. Recent multi-omics studies have identified diverse microglial subtypes, such as disease-associated microglia (DAM), neurodegenerative microglia (MGnD), white matter-associated microglia (WAM), and lipid-droplet-accumulating microglia (LDAM) [8, 10]. Although these subtypes represent promising therapeutic targets, translating this knowledge into precise clinical interventions remains a challenge.

A key breakthrough in microglia research was the discovery of *triggering receptor expressed on myeloid cells 2 (TREM2)* variants as risk factors for AD [11]. TREM2 is a membrane receptor expressed exclusively on microglia and plays a critical role in clearing toxic protein aggregates, including amyloid-beta (Aβ), TAR DNA-binding protein 43 (TDP-43), and alpha-synuclein (α-Syn) [12–15]. Notably, recently approved monoclonal antibodies (mAbs) against amyloid aggregates, such as Lecanemab, Aducanumab, and Donanemab, are all linked to microglial phagocytic function, highlighting the therapeutic relevance of enhancing this function in AD [16]. However, increased microglial phagocytosis may activate Fc receptors (FcRs), potentially triggering immune-related adverse effects, such as amyloid-related imaging abnormalities (ARIA) [17, 18]. A genome-wide association study (GWAS) has identified *Apolipoprotein E4* (*ApoE4*) as the primary genetic risk factor for ARIA, underscoring the importance of precision medicine and patient stratification in optimizing treatment strategies [19]. Ongoing clinical trials targeting microglia in AD, ALS, PD, and other NDDs are summarized in Table 1. This review focuses specifically on molecular targets related to microglial function, highlighting promising biomarkers with potential clinical applications. The general concept of biomarkers and conventional fluid biomarkers (e.g., Aβ and tau) [20, 21], will be briefly described. Following this, we will discuss future directions and remaining challenges in the development and clinical translation of microglia-related biomarkers.

**Table 1:** Summary of Clinical Trials Targeting Microglia in Neurodegenerative Diseases: Investigational Agents, Microglial Targets, Mechanisms, and Biomarker Insights (Data from ClinicalTrials.gov, Accessed April 2025)

Target	Investigational Agent	Mode of Action	NCT number	Clinical Study Title	Disease	Phase	Sponsor	Biomarkers	Reference
TREM2	AL002	mAb targeting TREM2 receptors	NCT04592874 (Completed)	A Phase 2 Study to Evaluate Efficacy and Safety of AL002 in Participants with Early AD	AD	Phase 2	Alector Inc	CSF sTREM2, neuroimaging biomarkers (amyloid PET, tau PET)	[22]
			NCT05744401 (Recruiting)	A Long-term Extension Study to Evaluate Safety, Tolerability, and Efficacy of AL002 in AD	AD	Phase 2	Alector Inc	CSF sTREM2, microglial signaling markers (CSF1R, osteopontin, IL-1RA), AD pathophysiology (pTau217, MTBR-Tau243, pTau181, Aβ42/40, amyloid PET, tau PET), astrogliosis (GFAP, YKL-40), neuronal/synaptic injury (NfL, neurogranin, volumetric MRI)	[23]
	VHB937		NCT06643481 (Recruiting)	A Clinical Trial to Learn About the Effects of VHB937 in People with ALS (ASTRALS)	ALS	Phase 2	Novartis	Ratio to baseline in NfL concentration in serum	[24]
	VG-3927	Small molecule TREM2 agonist	NCT06343636 (Completed)	A Phase 1 Study of VG-3927 in Healthy Adults and Patients With AD	Healthy, AD	Phase 1	Vigil Neuroscience	sTREM2 in CSF	[25]
CD33	AL003	mAb targeting CD33	NCT03822208 (Completed)	First in Human Study for Safety and Tolerability of AL003	AD	Phase 1	Alector Inc	Soluble CD33 in CSF, CD33 expression on blood monocytes	[26]
PGRN	AVB-101	Gene therapy using AAV-PGRN	NCT06064890 (Recruiting)	A Study to Evaluate the Safety and Effect of AVB-101, a Gene Therapy Product, in Subjects with a Genetic Sub-type of FTD (FTD-GRN) (ASPIRE-FTD)	FTD	Phase 1/2	AviadoBio Ltd	PGRN, NfL in CSF and blood, MRI (brain volumes)	[27]
	DNL593 (PTV:PGRN)	Recombinant PGRN protein	NCT05262023 (Active, not recruiting)	A Study to Evaluate the Safety, Tolerability, Pharmacokinetics, and Pharmacodynamics of DNL593 in Healthy Participants and Participants with FTD (FTD-GRN)	FTD	Phase 1/2	Denali Therapeutics Inc	DNL593 in CSF, DNL593 CSF: serum concentration ratio	[28, 29]
	Latozinemab (AL001)	mAb targeting SORT1	NCT04374136 (Active, not recruiting)	A Phase 3 Study to Evaluate Efficacy and Safety of AL001 in FTD (INFRONT-3)	FTD	Phase 3	Alector Inc	PGRN, NfL in CSF and blood, MRI (brain volumes)	[30–32]
			NCT05053035 (Terminated)	A Phase 2 Study to Evaluate AL001 in C9orf72-associated ALS	ALS	Phase 2	Alector Inc	PGRN, NfL in CSF and blood	_*
			NCT03636204 (Completed)	A First in Human Study in Healthy Volunteers and in Participants with FTD With Granulin (GRN) Mutation	FTD	Phase 1	Alector Inc	PGRN, NfL in CSF and blood, WBC sortilin	[31]
			NCT05363293 (Completed)	Multiple Ascending Dose Safety, Tolerability, PK Study of AL001 in AD Patients & Healthy Adult Subjects	AD	Phase 1/2	Alzamend Neuro, Inc	_	_*
	AL101	mAb targeting SORT1	NCT06079190 (Recruiting)	Efficacy and Safety of GSK4527226 [AL101] in Participants with Early AD (PROGRESS-AD)	AD	Phase 2	GlaxoSmithKline	_	_*
**Target**	**Investigational Agent**	**Mode of Action**	**NCT number**	**Clinical Study Title**	**Disease**	**Phase**	**Sponsor**	**Biomarkers**	**Reference**
ApoE2	LX1001	Gene-therapy using AAV-ApoE2	NCT03634007 (Active, not recruiting)	Gene Therapy for ApoE4 Homozygote of AD	AD	Phase 1/2	Lexeo Therapeutics	Neuroimaging biomarkers (Aβ-PET, Tau-PET, MRI), CSF studies	[33]
c-Abl	Nilotinib BE (AMN107)	Abl Tyrosine kinase inhibitor	NCT05143528 (Not yet recruiting)	Evaluating the Efficacy and Safety of Nilotinib BE in Subjects with Early AD (NILEAD)	AD	Phase 3	KeifeRx, LLC	Aβ40, Aβ42, Tau, p-Tau in blood and CSF, Neuroimaging biomarkers (Aβ-PET, Tau-PET)	[34]
CD38	Daratumumab	mAb targeting CD38	NCT04070378 (Completed)	Study of Daratumumab in Patients with Mild to Moderate AD	AD	Phase 2	Marc L Gordon, MD	CD38 + proportion of CD8 ^+^ CD4^−^ T cells	[35]
Complement C1q	ANX005	mAb targeting C1q	NCT04569435 (Completed)	Study of ANX005 in Adults With ALS	ALS	Phase 2	Annexon, Inc	C1q, NfL in serum	_*
Complement C5	Ravulizumab	mAb targeting C5	NCT04248465 (Terminated)	An Efficacy and Safety Study of Ravulizumab in ALS Participants	ALS	Phase 3	Alexion Pharmaceuticals, Inc	C5, NfL in serum	[36]
CSF-1R	[18F] CSF-23	PET Probe for CSF1R expression	NCT06148233 (Recruiting)	CSF1R PET Probe [18F] CSF-23 in AD Brain Imaging	AD	Not Applicable	Huashan Hospital	_	_*
CSF-1R	Edicotinib (JNJ-40346527)	Antagonist of the CSF-1 receptor	NCT04121208 (Terminated)	Microglial Colony Stimulating Factor-1 Receptor (CSF1R) in AD	AD	Phase 1	University of Oxford	CSF-1R ligands (CSF-1, IL-34) in CSF and Plasma	_*
Galectin-3	TB006	mAb targeting galectin-3	NCT05476783 (Active, not recruiting)	A Long-Term Extension Study to Assess the Safety of TB006 in Participants With AD	AD	Phase 2	TrueBinding, Inc	Plasma Aβ42, Aβ42/40 ratio, p-Tau181, NfL, and neuroimaging biomarkers (Aβ-PET)	_*
			NCT05074498 (Completed)	Study to Assess the Safety, Tolerability, Pharmacokinetics, Pharmacodynamics, and Efficacy of TB006 in Participants With AD	AD	Phase 1/2	TrueBinding, Inc	TB006 levels in plasma and CSF	_*
GLP-1	NLY01	GLP-1 receptor agonists	NCT04154072 (Completed)	A Clinical Study of NLY01 in Patient’s with Early PD	PD	Phase 2	Neuraly, Inc	_	[37]
	Exenatide		NCT04232969 (Active, not recruiting)	Exenatide Once Weekly Over 2 Years as a Potential Disease Modifying Treatment for PD (Exenatide-PD3)	PD	Phase 3	University College, London	_	[38]
GM-CSF	Sargramostim	Recombinant human GM-CSF	NCT04902703 (Recruiting)	Phase II Trial to Evaluate Safety and Efficacy of GM-CSF/Sargramostim in AD	AD	Phase 2	University of Colorado, Denver	Plasma Aβ40, Aβ42, total tau, UCH‐L1, GFAP, NfL	_*
			NCT01409915 (Completed)	Study of the Safety & Efficacy of Leukine® in the Treatment of AD	AD	Phase 2	University of Colorado, Denver	Plasma Aβ40, Aβ42, total tau, UCH‐L1, GFAP, NfL	[39]
IL-1β	Canakinumab	mAb targeting IL‐1β	NCT04795466 (Terminated)	Study of the Efficacy and Safety of Various Anti-inflammatory Agents in Participants With MCI or Mild AD	AD	Phase 2	Novartis Pharmaceuticals	Immunogenetic anti-agent antibody levels in serum, plasma, and CSF	_*
JAK	Baricitinib	JAK inhibitor	NCT05189106 (Recruiting)	Neurodegenerative AD and ALS (NADALS) Basket Trial	AD ALS	Phase 1/2	Massachusetts General Hospital	CSF concentrations of baricitinib, CCL2, PKR, phospho-PKR, pPKR/PKR ratio, CXCL10, IFNG, IL-6, NfL, Tau, pTau, and plasma TDP-43	_*
LRRK2	BIIB122 (DNL151)	LRRK2 inhibitor	NCT05418673 (Completed)	A Study to Assess if BIIB122 Tablets Are Safe and Can Slow Worsening of Early-Stage PD in Participants With Specific LRRK2 Genetic Variants Between the Ages of 30 and 80 Using the Movement Disorder Society-Unified Parkinson’s Disease Rating Scale (LIGHTHOUSE)	PD	Phase 3	Biogen	LRRK2-activity (autophosphorylated LRRK2 (pS1292-LRRK2) or Rab protein phosphorylation (e.g., pT73-Rab10))	[40]
			NCT05348785 (Recruiting)	A Study to Assess the Safety of BIIB122 Tablets and if it Can Slow the Worsening of Early-Stage Parkinson’s Disease in Participants Between the Ages of 30 and 80 (LUMA)	PD	Phase 2	Biogen	Total LRRK2 protein in CSF, LRRK2-activity	_*
			NCT06602193 (Recruiting)	Safety and Pharmacodynamic Effects of BIIB122 in Participants With LRRK2-Associated PD (LRRK2-PD)	PD	Phase 2a	Biogen	Total LRRK2 protein in CSF, LRRK2-activity	_*
	BIIB094	LRRK2-ASO	NCT03976349 (Completed)	A Study to Evaluate the Safety, Tolerability, and Pharmacokinetics of BIIB094 in Adults With PD	PD	Phase 1	Biogen	LRRK2 mRNA and protein levels	[41]
NF-kB	Bezisterim (NE3107)	ERK/NF-kB inhibitor	NCT04669028 (Completed)	A Phase 3 Study of NE3107 in Probable AD	AD	Phase 3	BioVie Inc	Inflammatory serum biomarker TNF-α, and CSF pTau and Aβ42	[42]
NLRP3	Dapansutrile (OLT1177)	Inhibitor of NLRP3	ISRCTN16806940	Anti-inflammatory Intervention with dapansutrile (OLT1177) for PD modification (DAPA-PD)	PD	Phase 2	Olatec Therapeutics Inc	Inflammatory biomarkers in blood including hsCRP, IL-1β, IL-18, IL-6, IFN-γ, TNF-α, ASC specks	[43]
	VTX3232		NCT06556173 (Recruiting)	Phase 2a Study of VTX3232 in PD	PD	Phase 2	Zomagen Biosciences Ltd	Inflammatory biomarkers in plasma and CSF	[44]
	VENT-02		NCT06822517 (Recruiting)	A Phase 1b Study of the NLRP3 Inhibitor VENT-02 in Patients with Mild to Moderate PD	PD	Phase 1/2	Ventus Therapeutics U.S., Inc	Inflammation and neurodegeneration biomarkers in plasma and CSF	[45]
	ZYIL1		NCT05981040 (Completed)	Efficacy, Safety, Tolerability, Pharmacokinetics, and Pharmacodynamics of ZYIL1 in Patients With ALS	ALS	Phase 2	Zydus Lifesciences Limited	Serum NfL	[46]
P2X7	P2X7R antagonists	P2X7 purinergic receptor blockers	NCT03918616 (Completed)	P2X7 Receptor, Inflammation and Neurodegenerative Diseases	NDDs	Observational Study	University of Pisa	P2X7R-inflammasome activity, NF-kB activity, serum α-synuclein, IL-1β, IL-18, miRNAs (miR-30 and miR-7)	_*
P2Y6	GC021109	Agonist of microglial P2Y6 receptor	NCT02386306 (Completed)	Study Evaluating Safety, Tolerability, and PK of Multiple Ascending Doses of GC021109 in Subjects with Mild to Moderate AD	AD	Phase 1	GliaCure, Inc	Volumetric MRI, PET (cortical metabolic rate), CRP, plasma (Aβ42/40 ratio, p-tau 217, GFAP, NfL, TNF, MCP-1), changes in DNA methylation, serum leptin and adiponectin	_*
p38α MAPK	Neflamapimod (VX-745)	p38α MAPK Inhibitor	NCT03435861 (Completed)	Effect of Neflamapimod on Brain Inflammation in AD Patients	AD	Phase 2	University Hospital, Toulouse	Brain inflammation using [18F] DPA-714, Blood and CSF biomarkers of inflammation (TNF-α, IL-1β, IFNg, IFNα/β/, IL-6, −8,−10,−12,−27, MCP-1, GM-CSF, chemokine receptors, PD-1, CD14/16, p-tau, Aβ40, Aβ42)	[47]
	MW150	p38α MAPK Inhibitor	NCT05194163 (Not yet recruiting)	MW150 Stress Kinase Inhibitor in Mild to Moderate AD (SKI-AD)	AD	Phase 2	Neurokine Therapeutics	Plasma cytokines (IFN-γ, IL-1β, IL-4, IL-5, IL-6, IL-8, IL-10, IL-12P70, IL-22, TNFα), neuronal biomarkers (Tau, NfL)	_*
PD-L1	IBC-Ab002	mAb targeting PD-L1	NCT05551741 (Recruiting)	A First in Human Study of IBC-Ab002 in Persons with Early AD	AD	Phase 1	Immunobrain Checkpoint	IBC-Ab002 levels in serum	[48]
RIPK1	SAR443060 (DNL747)	RIPK1 inhibitor	NCT03757351 (Terminated)	Study to Evaluate DNL747 in Subjects With ALS	ALS	Phase 1b	Sanofi	pS166-RIPK1 levels in PBMC	[49]
			NCT03757325 (Completed)	Study to Evaluate DNL747 in Subjects With AD	AD	Phase 1b	Denali Therapeutics Inc	pS166-RIPK1 levels in PBMC	[49]
	SAR443820 (DNL788)		NCT05237284 (Termicated)	Phase 2 Study for SAR443820 in Participants With ALS	ALS	Phase 2	Sanofi	pS166-RIPK1 levels in PBMC, serum NfL	[50]
SEMA4D	Pepinemab (VX15/2503)	mAb targeting SEMA4D	NCT04381468 (Completed)	SEMA4D Blockade Safety and Brain Metabolic Activity in AD	AD	Phase 1/2	Vaccinex Inc	FDG-PET (Brain metabolism), MRI (brain volume), serum and CSF biomarkers of inflammation, amyloid, and neurodegeneration	_*
Senescent cell	Dasatinib + Quercetin	Senolytic therapy combining dasatinib and quercetin (D + Q)	NCT05422885 (Completed)	Safety and Feasibility of Dasatinib and Quercetin in Adults at Risk for AD (STAMINA)	AD	Phase 1 Phase 2	Lewis Lipsitz	Senescent CD3 cells expressing p16, SASP factors (IL-1α, IL-6, MMP-9, −12) in blood and urine	_*
			NCT04785300 (Enrolling by invitation)	ALSENLITE: Senolytics for AD	AD	Phase 1 Phase 2	James L. Kirkland, MD, PhD	_	[51]
			NCT04685590 (Recruiting)	Senolytic Therapy to Modulate the Progression of AD (SToMP-AD) Study (SToMP-AD)	AD	Phase 2	Wake Forest University Health Sciences	CSF NfL, Inflammatory protein (plasma fractalkine and MMP-7, and CSF IL-6), SASP factors (eotaxin, MCP-1, VEGF) metabolic analysis in urine, lipidomics analysis in plasma and CSF, Transcriptomic analysis in PBMC	[51–53]
TLR2	NPT520-34	Small-molecule antagonist of TLR2	NCT03954600 (Completed)	A Study to Assess the Safety, Tolerability and PK of NPT520-34 in Healthy Subjects	Healthy	Phase 1	Neuropore Therapies Inc	DHEA-S, DHEA, Cortisol Free, Copeptin, Osmolality Serum, Prolactin, FSH, LH, TSH, T3 Free, T4 free, PGE2, Angiotensin II, Renin	_*
TNF-α	Pegipanermin (XPro1595)	Neutralization of soluble TNF-α	NCT05318976 (Recruiting)	A Study of XPro1595 in Patients With Early AD With Biomarkers of Inflammation	AD	Phase 2	Inmune Bio, Inc	Blood inflammatory and neurodegeneration biomarker amyloid, pTau, MRI (change in brain structure)	_*
			NCT05522387 (Active, not recruiting)	An Open-Label Extension of XPro1595 in Patients with Alzheimer’s Disease	AD	Phase 2	Inmune Bio, Inc	Blood inflammatory and neurodegeneration biomarker amyloid, pTau, Change in brain structure by MRI (neuroinflammation-White matter Free Water, axonal integrity- Apparent Fiber Density (AFD), Everyday Cognition (ECog))	_*
TSPO	[18F]-FEPPA	PET Probe for activated microglia by TSPO expression	NCT02945774 (Recruiting)	Molecular Neuroimaging of Neuroinflammation in Neurodegenerative Dementias	FTD	Not Applicable	Lawson Health Research Institute	MRI (cerebral perfusion, brain structure, white matter analysis)	[54]
	[11C] PBR28		NCT05205291 (Recruiting)	Molecular Imaging of Inflammation in PD Using LPS and TSPO-PET/MR	PD	Observational	University of Exeter	MRI, circulating molecules of inflammation levels in blood	[55]
			NCT04057807 (Completed)	Peripheral Benzodiazepine Receptors (PBR28) Brain PET Imaging with Lipopolysaccharide Challenge for the Study of Microglia Function in AD	AD	Early Phase 1	Yale University	Aβ-PET, Aβ in CSF	_*
Tyrosine kinase	Masitinib	Tyrosine kinase inhibitor	NCT05564169 (Not yet recruiting)	Masitinib in Patients with Mild to Moderate AD	AD	Phase 3	AB Science	_	[56]
			NCT03127267 (Recruiting)	Efficacy and Safety of Masitinib Versus Placebo in the Treatment of ALS Patients	ALS	Phase 3	AB Science	Cytochromes P450 (P450) 2C8 and 3A activity	[57]

^*^Clinical trial data were retrieved solely from ClinicalTrials.gov, and no published reference is available

*Abbreviations: AAV* Adeno-associated virus, *AD* Alzheimer’s disease, *AKAP8L* A-kinase anchor protein 8-like, *ALS* Amyotrophic lateral sclerosis, *ApoE* Apoprotein E, *ASO* Antisense oligonucleotide, *Aβ* Amyloid beta, *C1q* Complement component 1q, *C3* Complement component 3, *C5* Complement component 5, *c-Abl* Abelson tyrosine kinase, *CSF* Cerebrospinal fluid, *CSF1R* Colony stimulating factor 1 receptor, *DCI* Diffusion compartment imaging, *DTI* Diffusion tensor imaging, *GFAP* Glial fibrillary acidic protein, *GLP-1* Glucagon-like peptide-1, *GM-CSF* Granulocyte–macrophage colony-stimulating factor, *GPNMB* Glycoprotein NMB, *GRN* Granulin, *IL-1RA* Interleukin-1 receptor antagonist, *IL-1β* Interleukin-1 beta, *JAK* Janus kinase, *LRRK2* Leucine-rich repeat kinase 2, *mAb* Monoclonal antibody, *MCP-1* Monocyte chemoattractant protein-1, *miRNA* microRNA, *MRI* Magnetic resonance imaging, *NDDs* Neurodegenerative diseases, *NF-kB* Nuclear Factor Kappa B, *NfL* Neurofilament light chain, *NLRP3* NLR family pyrin domain containing 3, *P2X7* P2X purinoceptor 7, *P2Y6* P2Y purinoceptor 6, *p38 MAPK* p38 mitogen-activated protein kinases, *PD* Parkinson’s disease, *PD-L1* Programmed death-ligand 1, *PET* Positron emission tomography, *PGE2* Prostaglandin E2, *PGRN* Progranulin, *PKR* Protein-kinase R, *PTV* Protein transfer vehicle, *RIPK1* Receptor-interacting serine/threonine-protein kinase 1, *SASP* Senescence-associated secretory phenotype, *sCD22* Soluble CD22, *SORT1 *Sortilin, *SEMA4D* Semaphorin-4D, *sTREM2* Soluble TREM2, *TLR2* Toll-like receptor 2, *TNF-α* Tumor necrosis factor-α, *TREM2* triggering receptor expressed on myeloid cells 2, *TSPO* Translocator protein, *UCH‐L1* Ubiquitin C‐terminal hydrolase L1, *VEGF* Vascular endothelial growth factor, *YKL-40/CHI3L1* Chitinase 3-like 1

### Molecular targets of microglia in NDDs

#### Disease-associated genes of microglia: targets for ongoing and emerging clinical trials

**Fig. 1:**
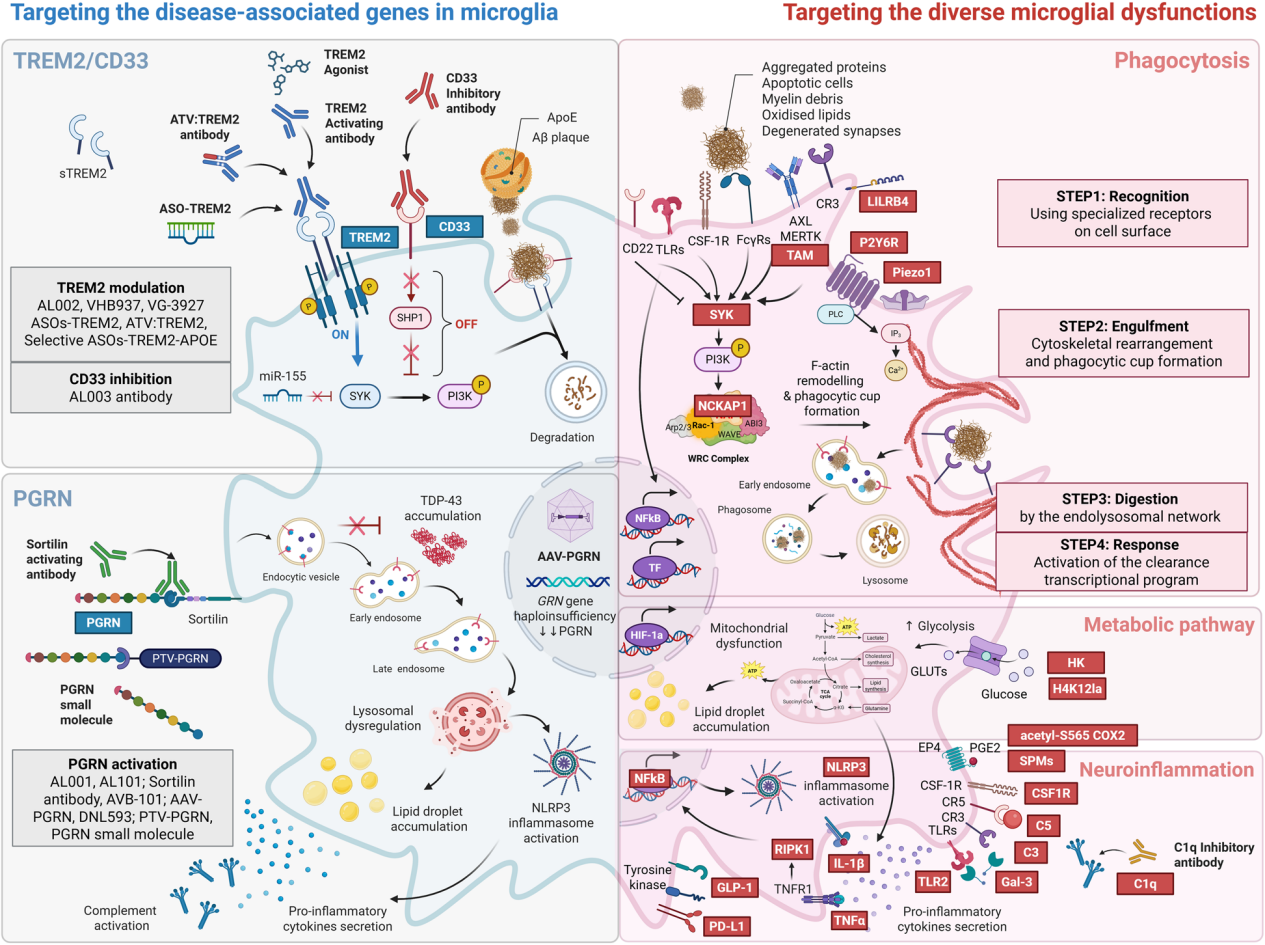
Therapeutic Strategies Targeting Microglia in Neurodegenerative Diseases. This figure illustrates therapeutic strategies targeting microglia in neurodegenerative diseases (NDDs), broadly categorized into two primary approaches: (Left)-Targeting Disease-Associated Genes: Therapies focused on modulating microglial genes such as *TREM2*, *CD33*, and *PGRN*. These pathways are critical in regulating microglial signaling, receptor activation, and cellular response to disease-related stressors. (Right)-Restoring Dysfunctional Microglial Functions: Interventions aimed at key microglial functions—phagocytosis, metabolism, and neuroinflammation—to repair dysfunction. The phagocytosis process, essential for clearing abnormal protein aggregates, apoptotic cells, myelin debris, synapses, and degenerated neurites, unfolds in four key steps: Step 1: Recognition via phagocytic receptors and engagement with find-me signals. Step 2: Engulfment facilitated by cytoskeletal rearrangement and phagocytic cup formation. Step 3: Digestion through the endolysosomal network. Step 4: Response involving activation of transcriptional programs and cytokine release. Microglial metabolism is tightly linked to both inflammatory responses and phagocytosis, highlighting its central role in maintaining microglial homeostasis. This interplay between genetic pathways and functional modulation provides a foundation for innovative therapeutic strategies. For detailed lists of investigational agents and specific targets, refer to Table 1 and Supplementary Table 1. Abbreviations: AAV; Adeno-associated virus, acetyl-S565 COX2: acetylation of serine 565 residues of cyclooxygenase-2, ApoE; Apoprotein E, ASO; Antisense oligonucleotide, Aβ; Amyloid beta, C1q; Complement component 1q, C3; Complement component 3, C5; Complement component 5, CSF: Cerebrospinal fluid, CSF1R; Colony stimulating factor 1 receptor, EP2; E-prostanoid receptor type 2, FcγR; Fc-gamma receptors, Gal-3; Galectin-3, GLP-1; Glucagon-like peptide-1, GLUTs; Glucose transporters, GPNMB; Glycoprotein NMB, GRN; Granulin, H4K12la; Histone H4 lysine 12 lactylation, HIF-1α; Hypoxia inducible factor 1 subunit alpha, HK2; Hexokinase 2, IL-1β; Interleukin-1 beta, JAK; Janus kinase, LILRB4; Leukocyte Ig-like receptor B4, miRNA; microRNA, NCKAP1; NCK associated protein 1, NDDs: Neurodegenerative diseases, NF-kB; Nuclear Factor Kappa B, NLRP3; NLR family pyrin domain containing 3, P2Y6R; P2Y6 receptor, PD-L1; Programmed death-ligand 1, PGE2; Prostaglandin E2, PGRN; Progranulin, PI3K; phosphatidylinositol 3-kinase, Piezo 1; Piezo type mechanosensitive ion channel component 1, PTV; Protein transfer vehicle, RIPK1; Receptor-interacting serine/threonine-protein kinase 1, SHP-1; Src-homology 2-domain-containing protein tyrosine phosphatase-1, SORT1; Sortilin, SPMs; Specialized pro-resolving mediators, sTREM2; Soluble TREM2, SYK; Spleen tyrosine kinase, TAM receptors; Tyro3, Axl and MerTK, TDP-43; TAR DNA-binding protein 43, TF; Transcription factor, TLR2; Toll-like receptor 2, TNF-α; Tumor necrosis factor-alpha, TREM2; Triggering receptor expressed on myeloid cells 2, WRC complex; WAVE regulatory complex

Advancements in next-generation sequencing have identified rare variants with strong disease associations, offering critical insight into microglial genes involved in NDD pathogenesis. Among these, *TREM2*, *CD33*, and *progranulin (PGRN)* have emerged as key candidates, and multiple clinical trials are underway to target microglia-specific dysfunction (Fig. 1 and Table 1).

NDDs, such as AD, ALS, FTD, and PD, share a common pathological hallmark: the accumulation of misfolded protein aggregates, including Aβ, TDP-43, and α-Syn [58]. Microglial phagocytosis plays a central role in the clearance of these aggregates, representing a promising therapeutic target. For instance, *C9orf72* mutations impair microglial clearance in ALS and FTD [59], while Aβ and p-tau accumulation in AD contribute to synaptic dysfunction and neurodegeneration. TREM2 regulates microglial phagocytosis and synaptic pruning, and its deficiency accelerates neuronal loss [60, 61]. Variants in *TREM2* have been identified as risk factors for AD, PD, FTD, and ALS [62–65]. Its exclusive expression in CNS-resident immune cells underscores the active role of microglia in NDD pathophysiology [12, 66]. GWAS have further linked the *TREM2* R47H variant to sporadic ALS and a 2- to 4-fold increased risk for AD [11, 62]. Other variants (R62H, T66M, H157Y, and D87N) modulate TREM2 expression and disease susceptibility [67].

#### TREM2 as a therapeutic target

TREM2 has emerged as a pivotal modulator of microglial responses in NDDs. Loss of TREM2 impairs Aβ clearance [68] and exacerbates tau pathology [69]. Conversely, strategies that upregulate TREM2, such as gene delivery, overexpression, or agonist antibodies, enhance microglial phagocytosis and cognitive performance in AD models [70]. Mechanistically, Aβ binds to TREM2, activating its downstream signaling and enhancing microglial phagocytosis [60]. However, tauopathy models show inconsistent results, suggesting context-dependent effects across disease stages [71, 72]. Beyond phagocytosis, TREM2 influences microglial survival and lipid metabolism [73, 74]. Nevertheless, concerns remain about its potential to promote microglial senescence, which limits its therapeutic utility in certain contexts [75]. Despite these challenges, therapeutic strategies targeting TREM2 continue to show promise. Several therapeutic trials are under development. AL002 (Alector) and VHB937 (Novartis) are TREM2-activating monoclonal antibodies (mAbs), while VG-3927 (Vigil Neurosciences) is a brain-penetrant small-molecule agonist. AL002c, an anti-TREM2 antibody, reduced Aβ plaque burden and improved cognition in the AD model [76]. In a Phase 1 study (INVOKE-1, NCT03635047), AL002 infusion resulted in a dose-dependent reduction in soluble TREM2 (sTREM2) in CSF, accompanied by increases in biomarkers of TREM2 signaling and microglial recruitment [77]. sTREM2, he cleavage product of TREM2, is considered a biomarker of increased microglial activation [78, 79]. Elevated CSF sTREM2 levels have been observed in AD, particularly during the early symptomatic stages [78, 80, 81]. However, in the AL002 study, the relationship between sTREM2 and increased microglial function may diverge, as AL002c appears to reduce sTREM2 levels by inducing receptor internalization and subsequent degradation, as indicated by reduced total TREM2 levels in brain lysates [77]. It is currently being investigated in two phase 2 clinical studies (NCT04592874 and NCT05744401) [82]. VHB937 increases TREM2 surface expression and activates downstream signaling (e.g., spleen tyrosine kinase (SYK) phosphorylation and calcium flux), enhancing microglial phagocytosis and chemotaxis [24]. In vivo*,* VHB937 reduced neuroinflammation, astrogliosis, and neuronal loss. A Phase 2 trial (NCT06643481) is currently underway to evaluate the efficacy and safety of VHB937 in participants with early-stage ALS (within 2 years of ALS symptom onset) over a 40-week period, followed by an open-label extension (ASTRALS). In contrast to antibody-based approaches, VG-3927 is a brain-penetrant small-molecule TREM2 agonist. In preclinical models, it reduced Aβ pathology and induced a DAM-like microglial phenotype [25]. A Phase 1 trial study (NCT06343636) demonstrated tolerability and dose-dependent reduction of CSF sTREM2, with data from AD patients expected in 2025 [83]. Additional TREM2-targeting therapy, 4D9, which facilitates clearance of Aβ and myelin debris in AD models [84]. And, DNL919 (ATV: TREM2), is a high-affinity human TREM2-activating antibody engineered with a monovalent transferrin receptor (TfR) binding site to enhance BBB penetration. While DNL919 showed target engagement, its Phase 1 trial (NCT05450549) was terminated due to reversible hematological changes at the highest dose [85]. These findings support TREM2 as a promising, albeit complex, therapeutic target in NDDs.

*CD33/Siglec-3* is another AD-susceptibility gene encoding a transmembrane sialic acid-binding receptor expressed on microglia [86]. Elevated CD33 expression suppresses microglial uptake of Aβ [87], while CD33 knockout enhances anti-inflammatory responses, reduces Aβ plaques, and improves cognitive function [88]. AL003, a CD33-blocking antibody (NCT03822208), was under development but discontinued in 2022. Nevertheless, interest in modulating CD33-mediated immune suppression remains [89].

#### PGRN as therapeutic targets

PGRN, encoded by the *GRN* gene, is a lysosomal regulatory protein essential for microglial function [90]. Loss of function of *GRN* mutations in FTD results in lysosomal impairment, accumulation of lipid droplets (LDs), and synapse loss through complement-mediated pruning [90–92]. C1qa deletion in *Grn*-deficient mice restored synapse density and extended survival [92]. *Grn* deficiency also reduces myelin debris clearance [93].

Novel therapies, such as AVB-101 (an AAV-based gene therapy) and DNL593 (a recombinant PGRN protein therapy), aim to restore PGRN levels and are currently undergoing clinical evaluation (NCT06064890, NCT05262023) [27, 28]. DNL593 consists of a PGRN protein fused to an antibody fragment that binds to the transferrin receptor. This association with transferrin receptors on BBB endothelial cells facilitates the receptor-mediated transcytosis of PGRN protein into the brain [28]. Interim results from the Phase 1/2 clinical trial showed that the total PGRN concentration in the CSF sampled 24 h after infusion also increased above baseline levels in a dose-dependent manner [29].

Additionally, Latozinemab (AL001), a monoclonal antibody targeting sortilin (SORT1) [30, 31], is under investigation not only for FTD (NCT04374136) but also for ALS (NCT05053035) and AD (NCT05363293 and NCT06079190). This antibody aims to increase PGRN levels by inhibiting the degradation pathway of PGRN, thereby offering potential therapeutic benefits across multiple NDDs. In the INFRONT-2 study, symptomatic FTD-*GRN* patients showed sustained normal plasma and CSF PGRN levels, reported normalization of lysosomal and inflammatory biomarkers, and exhibited stabilization of neurofilament light chain (NfL). In the Phase 2 trial for ALS related to *C9orf72* mutations, the primary objectives were safety, tolerability, pharmacokinetics, and pharmacodynamics, encompassing plasma and CSF PGRN levels and blood and CSF NfL concentrations as secondary outcomes. The Phase 3 study, INFRONT-3, aims to recruit 180 participants at risk of or diagnosed with FTD due to heterozygous mutations in the *GRN* gene. The trial includes CSF and plasma biomarkers assessing PGRN levels, along with multiple disease-relevant biomarkers of lysosomal function, complement activation, astrocyte function, neurodegeneration, and brain atrophy [32].

### Microglial functions: candidate targets in the preclinical level

**Fig. 2:**
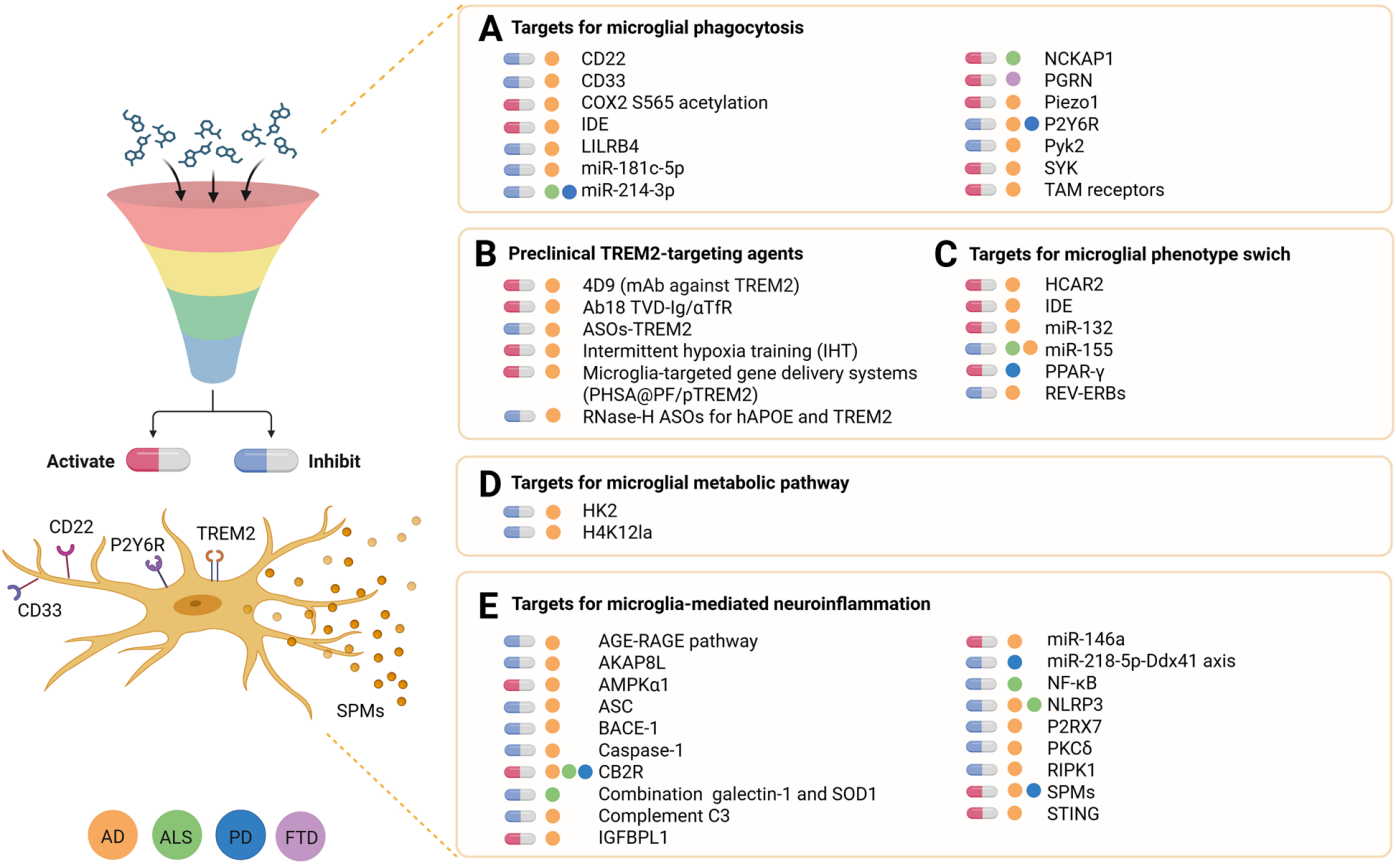
Molecular Targets and Investigational Agents Modulating Microglial Functions in Preclinical Studies. The molecular targets linked to microglial key functions, including (**A**) phagocytosis, (**B)** TREM2 signaling, (**C)** phenotype switching, (**D)** metabolic pathways, and (**E)** neuroinflammation, are highlighted in this figure. In vitro/in vivo studies have demonstrated that the functional recovery of microglia can be achieved through the activation or inhibition of these targets. The comprehensive list and detailed descriptions of these targets are provided in supplementary Table 1. Abbreviations: AAV; Adeno-associated virus, AD; Alzheimer’s disease, AKAP8L; A-kinase anchor protein 8-like, ALS; Amyotrophic lateral sclerosis, ApoE; Apoprotein E, ASO; Antisense oligonucleotide, BACE1; Beta-site amyloid precursor protein cleaving enzyme1, C3; Complement component 3, CB2R; Cannabinoid receptor type 2, cGAMP; Cyclic guanosine monophosphate–adenosine monophosphate, COX-2 S565; serine 565 of Cyclooxygenase-2, FTD; Frontotemporal dementia, GRN; Granulin, H4K12la; Histone H4 lysine 12 lactylation, HCAR2; Hydroxycarboxylic acid receptor 2, HK2; Hexokinase 2, IGFBPL1; Insulin-like growth factor binding protein-like 1, LXA4; Lipoxin A4, LILRB4; Leukocyte Ig-like receptor B4, mAb; Monoclonal antibody, miRNA (miR); microRNA, NCKAP1; NCK associated protein 1, N-AS; N-acetyl sphingosine, NLRP3; NLR family pyrin domain containing 3, P2Y6R; P2Y6 receptor, PD; Parkinson’s disease, PGRN; Progranulin, Piezo 1; Piezo type mechanosensitive ion channel component 1, Pyk2; Protein tyrosine kinase 2-beta, RIPK1; Receptor-interacting serine/threonine-protein kinase 1, RvD1; Resolvin D1**,** SCAP; Sterol regulatory element-binding protein (SREBP) cleavage-activating protein, SOD1; Superoxide dismutase type 1, SPMs; Specialized pro-resolving mediators, TAM receptors; Tyro3, Axl and MerTK, TDP-43; TAR DNA-binding protein 43, TREM2; Triggering receptor expressed on myeloid cells 2

Therapeutic strategies aimed at restoring microglial function by targeting markers associated with phagocytosis, metabolism, and neuroinflammation are schematically highlighted (Fig. 1, right side). Although several clinical trials targeting neuroinflammation are currently underway (Table 1), many targets related to microglial function modulation remain at the preclinical stage (Supplementary Table 1 and Fig. 2).

#### Microglial phagocytic function

Microglial phagocytosis—the clearance of extracellular debris, such as protein aggregates—is tightly linked to the pathology of NDDs [94]. This process consists of four key steps (Fig. 1): Step 1. Recognition of target molecules interacting with phagocytic receptors on the microglial surface (e.g., LILRB4, P2Y6R, Piezo1, TAM). Step 2. Engulfment, involving cytoskeletal rearrangement and phagocytic cup formation (e.g., SYK, NCKAP1). Step 3. Digestion of engulfed material within the endolysosomal network. Step 4. Response, including transcriptional activation and the release of cytokines [95].

CD22, an age-related genetic modifier of microglial phagocytosis, has been identified as a promising therapeutic target for NDDs associated with aging. Blocking CD22 has been shown to restore microglial function, enhance debris clearance, and improve cognitive performance in aged *Cd22*^*−/−*^ mice [96].

Microglial dysfunction often leads to chronic inflammation, which exacerbates the progression of NDDs. Specialized pro-resolving mediators (SPMs), including Lipoxin A4 (LxA4), Resolvin E1 (RvE1), and Resolvin D1 (RvD1), are crucial regulators of inflammation resolution [97, 98]. Specifically, SPMs promote neuronal survival and increase microglial phagocytosis [99, 100]. However, SPMs production and function are impaired in the AD brain [101]. A recent findings highlight sphingosine kinase 1 (SphK1) as a key regulator of SPMs production. In sphingolipid metabolism, SphK1 acts as an acetyltransferase of cyclooxygenase 2 (COX2), leading to the production of acetylation of serine 565 residues of COX2 (acetyl-S565 COX2), which enhances microglial phagocytosis and shifts microglia toward an anti-inflammatory phenotype. This results in reduced Aβ deposits, improved neuroinflammation, and restoration of memory in AD mouse models [102]. Administration of N-acetyl sphingosine (N-AS), an intermediate generated by SphK1, further enhanced acetyl-S565 COX2 levels and SPMs secretion (e.g., 15R-LxA4, RvE1, RvD1), thereby ameliorating AD pathologies. Notably, SphK1 induces acetylation of serine 565 residues of COX2 [103]. These findings highlight acetyl-S565 COX2 as a promising therapeutic target for improving microglial phagocytic function.

Comparative studies of human monocyte-derived microglia-like cells from slowly progressive versus rapidly progressive ALS revealed that reduced NCK associated protein 1 (NCKAP1) contributes to impaired phagocytosis and faster disease progression in ALS [104]. Elevated microRNA (miRNA)−214-3p levels targeting NCKAP1 were associated with phagocytic dysfunction and pro-inflammatory states in patients with rapidly progressing ALS. Inhibition of miR-214-3p restored defective microglial phagocytosis, correlating with slower disease progression. This suggests that miR-214-3p may be a reliable biomarker and a potential therapeutic target for ALS [105]. In PD, miR-214-3p inhibited autophagy and promoted apoptosis of dopaminergic neurons [106], indicating that miR-214-3p might be a potential target for modulating microglial dysfunction in NDDs.

The mechanosensitive Piezo1 channel, expressed in microglia, acts as a sensor of Aβ fiber stiffness [107]. Activation of Piezo1 reduces brain Aβ load and cognitive decline in AD models, identifying it as a promising therapeutic target [108, 109].

SYK, a non-receptor-type tyrosine kinase, regulates microglial phagocytosis and neuroinflammation. While SYK inhibition has been shown to reduce tau phosphorylation and oligomerization [110, 111], other studies suggest that SYK activity downstream of TREM2 and CLEC7A promotes the transition of microglia into DAM and enhances phagocytosis [112, 113]. These findings suggest that careful modulation of SYK activity may be necessary to balance its dual roles in neurodegenerative conditions.

TAM receptors–Tyro3, Axl, and Mertk–are critical mediators of microglial recognition and clearance of apoptotic cells and cellular debris. In the APP/PS1 mouse model of AD, TAM receptor signaling has been shown to promote the formation of dense-core amyloid plaques, emphasizing their complex and dualistic role in the pathology of NDDs [114]. The TAM receptor ligands growth arrest-specific 6 (Gas6) and protein S (PROS1), which are essential for receptor activation, may serve as potential biomarkers or therapeutic targets to modulate microglial responses via TAM signaling [115]. Notably, a novel fusion protein, αAβ–Gas6, which utilizes TAM receptor-mediated phagocytosis without eliciting inflammatory responses, has demonstrated enhanced Aβ clearance and behavioral improvement, with fewer adverse effects, compared to conventional Aβ-targeting antibodies [116].

Additionally, several therapeutic strategies have been explored to reduce Aβ pathology, with a focus on enhancing microglial phagocytosis. One approach involves upregulating insulin-degrading enzyme (IDE), a zinc-metalloprotease responsible for degrading insulin and Aβ peptides [117, 118]. Increased IDE expression or activity has been shown to reduce soluble Aβ levels, attenuate plaque deposition, and mitigate neurotoxicity in preclinical models [119, 120]. Other compounds act more directly on microglial phagocytic function. For instance, the purinergic receptor P2Y6 responds to extracellular nucleotides released during cellular injury. Agonists of P2Y6 enhance phagocytosis, shifting microglial behavior toward active surveillance and debris clearance [121].

Human leukocyte Ig-like receptor B4 (LILRB4) is an inhibitory receptor of the immunoglobulin (Ig) superfamily that is expressed on myeloid cells and recognizes apolipoprotein E (ApoE). LILRB4 is highly expressed in the microglia of AD patients and has also emerged as a potential therapeutic target [122]. In transgenic mice carrying a portion of human *LILR* that includes *LILRB4,* systemic treatment with an anti-human LILRB4 mAb reduced Aβ load, mitigated Aβ-related behavioral abnormalities, enhanced microglial activity, and attenuated the expression of interferon-induced genes [122]. Thus, LILRB4 modulation may represent a promising therapeutic approach for AD. Retinoid X receptor (RXR) agonists, such as bexarotene, and nuclear receptors, including PPARγ and RXR, have been shown to facilitate Aβ clearance in AD mouse models; however, clinical translation has remained challenging [123]. Interleukin (IL)−33, a cytokine belonging to the IL-1 family, also promotes microglial recruitment and Aβ clearance through the ST2/p38 signaling pathway, leading to a reduction in soluble Aβ and amyloid plaque burden [124]. These findings underscore the therapeutic potential of targeting microglial phagocytic activity to enhance Aβ clearance in AD.

Supplementary Table 1 provides an overview of preclinical studies exploring microglia-targeting strategies for NDD treatment. These include novel approaches targeting phagocytic receptors, inflammation modulators, and microglial metabolism pathways. These promising interventions remain under investigation, offering potential avenues for future clinical application.

#### Functional phenotype switching

Research into the contributions of microglia to ALS has advanced considerably, paralleling progress in AD. Early studies in superoxide dismutase 1 (SOD1) mouse models proposed that inflammatory responses from microglia play a pivotal role in ALS progression [125]. This hypothesis has fueled numerous clinical trials testing anti-inflammatory therapies. Furthermore, degenerative changes in motor neurons were observed in mice with *SOD1* mutations selectively induced in microglia, emphasizing a non-cell-autonomous mechanism underlying ALS progression [126].

In SOD1 mice, motor neuron degeneration progresses slowly during the early stages of disease, coinciding with an increase in microglia’s anti-inflammatory (M2) phenotype [127]. However, as the disease advanced, a switch to the pro-inflammatory (M1) phenotype accelerated motor neuron degeneration and worsened clinical outcomes [128]. Although the M1/M2 framework is now recognized as oversimplified, with multi-omics studies revealing substantial microglial heterogeneity based on factors such as brain region, sex, and disease context, these findings highlighted the critical role of microglia in determining ALS progression speed.

Mesenchymal stromal cells (MSCs) exert potent immunomodulatory effects on microglia through direct cell–cell interactions and paracrine signaling. One key mechanism involves the release of extracellular vesicles (EVs) that encapsulate bioactive molecules, including cytokines, chemokines, growth factors, and microRNAs, which mirror the anti-inflammatory and regenerative properties of MSCs [129, 130]. MSC-derived EVs (MSC-EVs) are internalized by microglia and have been shown to suppress the expression of pro-inflammatory mediators such as tumor necrosis factor-alpha (TNF-α) and nitric oxide (NO), both of which are prominently elevated in NDDs and CNS injury [131]. Furthermore, MSCs restore microglial phagocytic capacity and promote a shift from a pro-inflammatory (M1-like) to an anti-inflammatory (M2-like) phenotype by secreting transforming growth factor-beta (TGF-β) [132]. These findings support the therapeutic potential of MSC-EVs in alleviating neuroinflammation and restoring microglial homeostasis in NDDs. This mechanism was supported by results from ALS clinical trials [133, 134], suggesting that TGF-β could serve as a biomarker for modulating microglial phenotype and should be further investigated.

miRNAs are also crucial regulators of microglial function and neuroinflammation in NDDs. A recent systematic review cross-validated 250 miRNAs associated with AD, highlighting some miRNAs (miR-107, miR-26b, miR-30e, miR-34a, miR-485, miR-200c, miR-210, miR-146a, miR-34c, and miR-125b) in patients’ peripheral blood [135]. Specific miRNAs, such as miR-146a, miR-155, miR-190, miR-124, and miR-30a, have a significant impact on regulating microglial functions, from influencing the cells’ activation states to modulating their responses to pathological stimuli [136]. For instance, in AD, miR-155 and miR-191-5p influence protective and inflammatory responses [137, 138]. Overexpression of miR-146a in microglia benefits APP/PS1 transgenic mice by reducing cognitive impairment and neuroinflammation, suggesting the therapeutic potential of miR-146a [139]. Deletion of miR-155 induces a pre-MGnD activation state via interferon-gamma (IFN-γ) signaling. Blocking IFN-γ signaling attenuates MGnD induction and impairs microglial phagocytosis [138]. In ALS, miR-125b and miR-155 modulate nuclear factor kappa-B (NF-κB) signaling, affecting microglial activation [140, 141]. Deletion of miR-155 improves microglial phagocytosis, delays disease onset, and extends survival in SOD1 mice [142]. In PD, miR-124 and miR-132-3p regulate microglial inflammation and autophagy [143, 144].

Hydroxycarboxylic acid receptor 2 (HCAR2), a niacin receptor expressed in microglia, is markedly induced by amyloid pathology in AD. Activation of HCAR2 using Food and Drug Administration (FDA)-approved niacin (Niaspan) enhances the microglial protective response and attenuates amyloid pathology, highlighting its therapeutic potential in modulating microglial phenotypes [145].

#### Microglial metabolic pathway

A recent meta-analysis of genetic risk factors for AD, identified through GWAS, revealed a strong enrichment of genes related to lipid metabolism and innate immunity, alongside those involved in amyloid and tau processing [146]. *ApoE*, the strongest genetic risk factor for late-onset AD [147], influences numerous processes, including amyloid aggregation and clearance, tau-induced neurodegeneration, glucose metabolism, and synaptic function [148]. The ApoE protein, critical for lipid homeostasis, mediates the transport of cholesterol and lipids between cells. ApoE expression is notably upregulated in microglia in AD [149]. Microglia expressing ApoE4 exhibit exaggerated immune responses and a diminished capacity to clear Aβ compared to microglia expressing ApoE2 or ApoE3 [150, 151]. In addition to its effects on glucose metabolism, ApoE4 disrupts lipid homeostasis in microglia, leading to LDs accumulation and impaired lipid processing, particularly in the context of amyloid pathology [152]. Recently, single-nucleus RNA sequencing studies have identified an enrichment of LD-rich microglia in ApoE 4/4 AD brains [153], linking ApoE, particularly the ApoE4 isoform, to the alteration of microglial metabolism by disrupting multiple metabolic pathways, leading to impaired immune responses and the exacerbation of AD pathology [154, 155].

One of the major receptors of ApoE is TREM2 [156], which can modify metabolic pathways, including oxidative phosphorylation, glycolysis, and lipid metabolism, thereby regulating microglial activation states and functions with implications for NDDs [74]. Accumulating evidence suggests that TREM2 deficiency results in impaired lipid metabolism in microglia. In TREM2-deficient microglia, dysregulation of lipid metabolism–related genes leads to the accumulation of cholesterol esters and impaired cholesterol transport within the brain [74]. Similarly, alterations in microglial metabolic pathways have been observed in the brains of AD patients carrying the *TREM2* R47H variant [157]. In addition, *TREM2* R47H loss-of-function in human induced pluripotent stem cell (iPSC)-derived microglia resulted in significant metabolic deficits, including reduced mitochondrial respiratory capacity and an inability to perform a glycolytic immunometabolic switch [158].

Microglial immunometabolism has been implicated in broader pathomechanisms related to AD. For instance, exposure to Aβ triggers acute microglial inflammation, accompanied by metabolic reprogramming from oxidative phosphorylation to glycolysis via the hypoxia-inducible factor (HIF)−1α pathway [159]. And, HIF plays an essential role in the cellular response to low oxygen, orchestrating a metabolic switch that allows cells to survive in inflammatory or immune-mediated diseases [160]. This change subsequently leads to the inhibition and breakdown of mitochondrial energy metabolism [161, 162]. Furthermore, prolonged exposure to Aβ plaques, neurofibrillary tangles, and excessive cell debris significantly alters overall lipid metabolism, leading to the accumulation of LDs [163]. Microglia with excessive LDs accumulation, known as LDAMs, exhibit significantly reduced phagocytic ability and elevated reactive oxygen species (ROS) production, promoting neuroinflammation in AD [163, 164]. These metabolic and epigenetic adaptations contribute to chronic microglial activation, defective clearance of pathological proteins, and sustained neuroinflammation—hallmarks of AD pathology. Targeting these pathways may offer novel strategies for restoring homeostatic microglial function in NDDs. Regulating lipoprotein lipase via hexokinase 2 (HK2) inhibition [165] and CD36 overexpression enhances remyelinating functions in aged microglia [166]. Histone H4 lysine 12 lactylation (H4K12la) drives a glycolysis-pyruvate kinase M2 (PKM2) feedback loop that exacerbates microglial dysfunction, contributing to AD. Pharmacological inhibition of PKM2 ameliorates this dysfunction, demonstrating a link between microglial glucose metabolism and AD pathology [167].

Prostaglandin E2 (PGE2) is another key player in microglial metabolism. Elevated PGE2 levels have been observed in the spinal cords, CSF, and serum of patients with ALS [168, 169] and the CSF of patients with AD [170]. Pharmacological inhibition of PGE2 receptors (EP2/EP4) enhances microglial energy metabolism, converting glycogen to glucose and restoring phagocytic function [171]. Additionally, TDP-43, a protein that regulates cellular metabolism, links microglial dysfunction to metabolic reprogramming. TDP-43 mislocalization disrupts metabolic pathways and exacerbates Aβ pathology, while its depletion promotes Aβ clearance by microglia [172, 173]. These findings emphasize the role of metabolic reprogramming in restoring microglial function and highlight its therapeutic potential for mitigating NDD progression.

#### Microglial-mediated neuroinflammation

Neuroinflammation is undeniably a key contributor to the pathogenesis of NDDs. However, previous research often approached neuroinflammation as a purely aggravating factor, neglecting a comprehensive, multidimensional perspective that considers genetic background, disease onset, progression, and environmental influences. This limited approach is likely to explain the lack of success in neuroinflammation-targeted treatments to date. As research progresses, a more nuanced understanding of neuroinflammation has emerged, emphasizing the complexity of cellular interactions, stages of neuroinflammatory progression, and the specific roles of microglia. This evolving knowledge strengthens the potential of neuroinflammation-targeted therapies as viable treatment strategies.

##### Ongoing clinical trials targeting neuroinflammation

Several therapeutic agents targeting neuroinflammation are currently under clinical investigation (Table 1 and Fig. 1). Masitinib, a tyrosine kinase inhibitor, has demonstrated potential in reducing neuroinflammatory processes and is being evaluated in AD (NCT05564169), ALS (NCT03127267), and multiple sclerosis (MS) (NCT01433497) [174, 175]. Canakinumab, an IL-1β inhibitor neutralizing antibody, is being investigated for its anti-inflammatory effects in AD (NCT04795466) [176]. Pegipanermin (XPro1595), a selective inhibitor of soluble TNF-α, is currently in clinical trials for AD (NCT05318976) [177]. Galectin-3 inhibitors, such as TB006, are being evaluated for their potential to modulate glial activity and reduce amyloid-associated inflammation in AD (NCT05074498, NCT05476783) [178, 179]. Additionally, P2X7 receptor inhibitors, which target microglia-driven neuroinflammatory signaling, are being explored across various NDDs [180]. NLRP3 inflammasome inhibitors, including VTX3232 (NCT06556173), Dapansutrile (OLT1177) (ISRCTN16806940), VENT-02 (NCT06822517), and ZYIL1 (NCT05981040), are currently under investigation for their ability to suppress innate immune activation and downstream neurotoxic cascades [43, 44, 46, 181].

##### Key pathways and targets in microglia-mediated neuroinflammation

NOD-like receptor family pyrin domain containing 3 (NLRP3) is a key pattern recognition receptor highly expressed in microglia and astrocytes, where it forms an inflammasome complex with apoptosis-associated speck-like protein containing a caspase recruitment domain (ASC) and caspase-1, triggering neuroinflammation in response to cellular stress or damage. Activation of the NLRP3 inflammasome releases IL-1β and IL-18, inducing pyroptosis and contributing to immune and inflammatory responses in the brain [182]. Recent studies have shown that aberrant NLRP3 activation plays a pathogenic role in various NDDs, including MS, PD, AD, and stroke. A range of therapeutic strategies is being developed to inhibit NLRP3 inflammasome activation, including both direct inhibitors and indirect approaches targeting key components or processes associated with NLRP3 inflammasome activation, including inhibiting NF-κB activation, IL-1β production, ASC and caspase-1 activation, blocking chloride ion efflux, and mitochondrial damage [183]. Various NLRP3 inhibitors show efficacy in preclinical studies, and several are currently in clinical trials, including AD, PD (OLT1177, VTX3232, VENT-02), and ALS (ZYIL1). VTX3232, a CNS-penetrant oral NLRP3 inhibitor by Ventyx Biosciences, showed safety and IL-1β inhibition in a Phase 1 trial and is now in a Phase 2a trial for early PD (NCT06556173)[44]. Dapansutrile (OLT1177), another oral NLRP3 inhibitor, has been shown to improve cognition and reduce pathology in AD and PD models [45, 184]. It is also entering a Phase 2 trial for PD (ISRCTN16806940) [43]. VENT-02, a novel brain-penetrant compound, is undergoing Phase 1 testing in PD (NCT06822517) [181]. Inhibiting specific components of the canonical NLRP3 inflammasome signaling pathway is another approach. ZYIL1, developed by Zydus Lifesciences, inhibits the NLRP3 pathway by blocking ASC oligomerization and is in a Phase 2 trial for ALS (NCT05981040) [46]. VX-740 and VX-765, selective caspase-1 inhibitors, block the release of IL-1β and IL-18, reducing inflammation. Preclinical studies have shown that these inhibitors can improve cognitive function in AD mouse models [185]. Additionally, the ASC-targeting antibody ACI-6635 has effectively reduced amyloid plaque size and ASC burden in APP/PS1 mice [186].

Receptor-interacting protein kinase 1 (RIPK1), a Ser/Thr kinase with a death domain, mediates deleterious mechanisms downstream of type I TNF-α receptor and is highly expressed in microglia in human AD brains [187]. Increased RIPK1 activation is implicated in various NDDs, including AD, ALS, and PD [188–190]. RIPK1 inhibition, either pharmacologically or genetically, reduces neuroinflammation, decreases Aβ accumulation (via downregulation of cystatin F encoded by *Cst7*), and improves behavioral deficits by enhancing the microglial clearance of Aβ in APP/PS1 mice [189]. Numerous RIPK1 inhibitors are currently in clinical trials for inflammatory diseases, including AD (NCT03757325) and ALS (NCT03757351, NCT05237284) [49, 50, 191–193]. However, no RIPK1 inhibitors have reached phase III trials, as dosing strategies for long-term safety and efficacy remain under optimization.

Leucine-Rich Repeat Kinase 2 (LRRK2)/PARK8, encoded by *LRRK2*, is the most common genetic mutation in both familial and sporadic PD [194]. Upon stimulation by toll-like receptor (TLR)-2 or TLR-4, increased expression and phosphorylation of LRRK2 in microglia drive pro-inflammatory responses [195]. LRRK2 inhibition modulates microglial inflammation by interfering with NF-κB signaling and suppressing phosphorylation of the NF-κB inhibitory subunit p50 [196]. Genetic variants, such as rs6581593 in the *LRRK2* locus, are associated with an increased risk of PD [197]. Biogen and Denali Therapeutics are conducting Phase 3 trials for BIIB122, a small-molecule LRRK2 kinase inhibitor, in PD patients with *LRRK2* mutations (NCT05418673, NCT06602193) [198]. LRRK2-mediated centrosomal alterations in peripheral blood-derived cells have been proposed as a biomarker for PD progression and patient stratification [199].

DJ-1, encoded by the *PARK7* gene, regulates microglial inflammatory responses. DJ-1 knockdown increases microglial production of pro-inflammatory cytokines and oxidative stress, while DJ-1 deficiency amplifies mitochondrial ROS production, exacerbating neuroinflammation [200–202]. DJ-1 knockout mice exhibit increased STAT1 phosphorylation and elevated levels of inflammatory mediators, such as COX-2, iNOS, and TNF-α [201]. Restoring microglial phagocytic function through approaches such as metabolic reprogramming and autophagy enhancement may hold promise for treating NDDs like AD, ALS, FTD, and PD. Identifying reliable biomarkers for microglial activity and neuroinflammation will be critical for advancing targeted therapies. Continued exploration of these microglia-related pathways will provide deeper insights into disease mechanisms and inform the development of novel therapeutic strategies.

### Classification of biomarkers in the BEST resource and potential biomarkers in NDDs

The FDA-National Institutes of Health (NIH) Biomarker Working Group classified biomarkers according to their intended use [203]. Biomarkers serve as indicators of normal biological processes, pathological processes, or responses to therapeutic interventions. They may include molecular, histologic, radiographic, or physiologic characteristics, but not direct measures of how a person feels, functions, or survives. Categories include safety, monitoring, predictive, prognostic, response, susceptibility/risk, and diagnostic biomarkers. Definitions and examples of each category are summarized in Table 2, based on the Biomarkers, EndpointS, and other Tools (BEST) Resource [204].

**Table 2:** Category and definition of biomarkers in the BEST (Biomarkers, EndpointS, and other Tools) resource and potential biomarkers for NDDs

BEST Resource (FDA)			Potential Biomarkers in NDDs		
**Biomarker Category**	**Definition**	**Examples**	**AD**	**PD**	**ALS/FTD**
1. Diagnostic Biomarker	A biomarker is used to detect or confirm the presence of a disease or condition of interest or to identify individuals with a subtype of the disease	*CFTR* mutations for cystic fibrosis HbA1c for Type 2 diabetes mellitus (DM) [205] Glomerular filtration rate (GFR) for chronic kidney disease [206]	Causative genes of AD* (***APP***, ***PSEN1***, ***PSEN2***) [207, 208] CSF sTREM2* [78–81, 209, 210] sCD22* [211] acetyl-S565 COX2* [103] Amyloid pathology: **Amyloid PET**, **Aβ42**, **p-tau217**, **p-tau181**, **p-tau231** [212] Tau pathology: **MTBR-tau243**, **p-tau205** [212]	Causative genes of PD* (***LRRK2****, ****DJ-1****, ***etc**.) [194, 213–215] **α-Syn** [216] Dopamine transporter (DAT) **Single photon emission computed tomography (SPECT)**, **F-DOPA PET** [217]	Causative genes of ALS/FTD* (***SOD1***, ***C9orf72***, ***TDP-43*, etc*.***) [218, 219] NfL* [220] TDP-43 pathology: HDGFL2 [221]
			PGRN* [222, 223], CX3CL1* [224], CHI3L1/YKL-40* [225–227] Synapse loss markers: SNAP-25 [228], GAP-43 [229], Ng [230–232] **GFAP** [233–235]		
2. Monitoring Biomarker	A biomarker is measured repeatedly to assess the status of a disease or medical condition or to demonstrate the effect of a medical product	CA-125 in ovarian cancer [236, 237]	PGRN* [222] CTSB* [238, 239] SPMs* [99, 101, 240] CD33* [241] IL-8, MCP-1* [242, 243] **Amyloid PET** [244, 245] **Tau PET** [244]	**α-Syn** [246]	CHIT1* [247, 248] Pro-inflammatory cytokines (e.g., IL-6, IL-8, MCP-1)* [249, 250] miR-214-3p* [105] TGF-β* [133, 134]
3. Pharmacodynamic /Response Biomarker	A biomarker is used to show that a biological response, potentially beneficial or harmful, has occurred in an individual exposed to a medical product or an environmental agent	The urinary level of glycosaminoglycans [251]	TSPO PET* [252, 253] sTREM2* [209, 254] YKL-40* [225–227] MCP-1* [255] **p-tau217**, **p-tau181**, **p-tau213** [212] **Aβ42/Aβ40 ratio** **Amyloid PET** **Tau PET** **FDG PET** **Hippocampal volume** **GFAP** [256] NfL*, VILIP-1, GAP-43 [255]	TSPO PET* [252, 253] - Dopamine levels in CSF or PET imaging α-Syn [246]	NfL* [257] SOD1* [258, 259] DRP [260, 261]
4. Predictive Biomarker	A biomarker is used to identify individuals more likely than similar individuals without the biomarker to experience a favorable or unfavorable effect from exposure to a medical product or an environmental agent companion diagnostics (CDx)	BReast CAncer genes 1 and 2 (*BRCA1/2*) mutations may be used as predictive biomarkers when evaluating women with platinum-sensitive ovarian cancer, to identify patients likely to respond to Poly (ADP-ribose) polymerase (PARP) inhibitors [262]	Companion diagnostics (CDx): not yet developed		
			Candidates: Tau PET [263] ApoE ε4 genotype* [17]	Candidates: α-Syn [246] Complementary Diagnostics (CoD): Dopamine transporter imaging (e.g., DaTscan)	
5. Prognostic Biomarker	A biomarker is used to identify the likelihood of a clinical event, disease recurrence, or progression in patients who have the disease or medical condition of interest Biomarkers indicate the likely progression, severity, or survival of a disease, independent of treatment	Increasing prostate-specific antigen (PSA) with prostate cancer during follow-up, to assess the likelihood of cancer progression [264]	NfL* [265] miR-214*(unpublished data) **p-tau181** and **p-tau217** [266, 267] sTREM2* [268] **GFAP, total tau, NfL** [269] YKL-40*, VILIP-1 [270, 271] **Amyloid PET** [272]	NfL* [273] **GFAP** [274]	NfL* [275–278] pNfH [279, 280] miR-214* [105]
6. Safety Biomarker	A biomarker is measured before or after exposure to a medical product or an environmental agent to indicate the likelihood, presence, or extent of toxicity as an adverse effect	HLA-B*1502 allele may be used as a safety biomarker to screen patients prior to initiating carbamazepine treatment, corrected QT interval (QTc) [281]	ARIA* [258] **High cerebral amyloid angiopathy (CAA)** [282, 283]	_	_
7. Susceptibility /Risk Biomarker	A biomarker that indicates the potential for developing a disease or medical condition in an individual who does not currently have a clinically apparent disease or medical condition	*ApoE* gene variations may be used as susceptibility/risk biomarkers to identify individuals with a predisposition to develop AD [284, 285]	APP*, PSEN1*, PSEN2* [207] ApoE ε4 genotype* [286] **Amyloid PET** [272] TREM2 variants* [11] CAA [282, 283]	PARK*, GBA1*, MAPT*, DJ-1*, LRRK2* [217, 287]	NfL* [288–290] SOD1* [291] C9orf72* [292, 293] SCA2 repeat number [294]

^*^Biomarkers linked to microglial function

Biomarkers identified in multiple clinical studies are shown in bold

*Abbreviations: acetyl-S565 COX2* acetylation of serine 565 residues of cyclooxygenase-2, *AD* Alzheimer’s disease, *ALS* Amyotrophic lateral sclerosis, *ApoE* Apolipoprotein E, *APP* Amyloid-beta precursor protein, *ARIA* Amyloid-related imaging abnormalities, *Aβ* Amyloid β, *BEST *Biomarkers, EndpointS, and other Tools, *BRCA1/2* Breast cancer genes 1 and 2, *C9orf72* chromosome 9 open reading frame 72, *CAA* Cerebral amyloid angiopathy, *CDx* Companion diagnostics, *CHIT1* Chitinase-1, *CoD* Complementary diagnostics, *CSF* Cerebrospinal fluid, *CTSB* Cathepsin B, *DAT* Dopamine transporter, *DM* Diabetes mellitus, *DRP* Dipeptide repeat proteins, *FTD* Frontotemporal dementia, *GAP-43* Growth-associated protein 43, *GFAP* Glial fibrillary acidic protein, *GFR* Glomerular filtration rate, *HDGFL2* Hepatoma-derived growth factor-like protein 2, *IL-6* Interleukin-6, *IL-8* Interleukin-8, *LRRK2* Leucine-rich repeat kinase 2, *MCP-1* Monocyte chemoattractant crotein-1, *miRNA* microRNA, *NfL* Neurofilament light chain, *Ng* Neurogranin, *PARP* Poly (ADP-ribose) polymerase, *PD* Parkinson’s disease, *PET* Positron emission tomography, *PGRN* Progranulin, *PSA* Prostate-specific antigen, *PSEN* Presenilin, *QTc* Corrected QT interval, *SCA2* Spinocerebellar ataxia type 2, *sCD22* Soluble CD22, *SPECT* Single photon emission computed tomography, *SNAP-25* Synaptosomal-associated protein 25, *SOD1* Superoxide dismutase type 1, *SPMs* Specialized pro-resolving mediators, *sTREM2* Soluble TREM2, *TDP-43* TAR DNA-binding protein 43, *TREM2* Triggering receptor expressed on myeloid cells 2, *TSPO* Translocator protein, *VILIP-1*Visinin-like protein 1, *YKL-40/CHI3L1* Chitinase 3-like 1, *α-Syn* Alpha-synuclein

A major challenge in designing clinical trials for NDDs, including AD, PD, and ALS, is achieving appropriate patient stratification through the use of reliable biomarkers. NDD pathology heterogeneity underpins variability in symptoms and progression rate, necessitating biomarkers to stratify patients, monitor treatment efficacy, predict prognosis, and evaluate safety. The European Medicines Agency (EMA) and the FDA highlight that carefully selected biomarkers can reduce the risk of trial failure by identifying study populations more likely to respond to therapies [295].

Despite advances, few biomarkers have been validated for clinical use in NDDs. Nevertheless, numerous candidates show promise for diagnosing, monitoring, and predicting disease progression, as detailed in Table 2. Many biomarkers overlap, but microglia-related biomarkers, in particular, stand out for their potential utility.

### Current and emerging biomarkers in NDDs

**Fig. 3:**
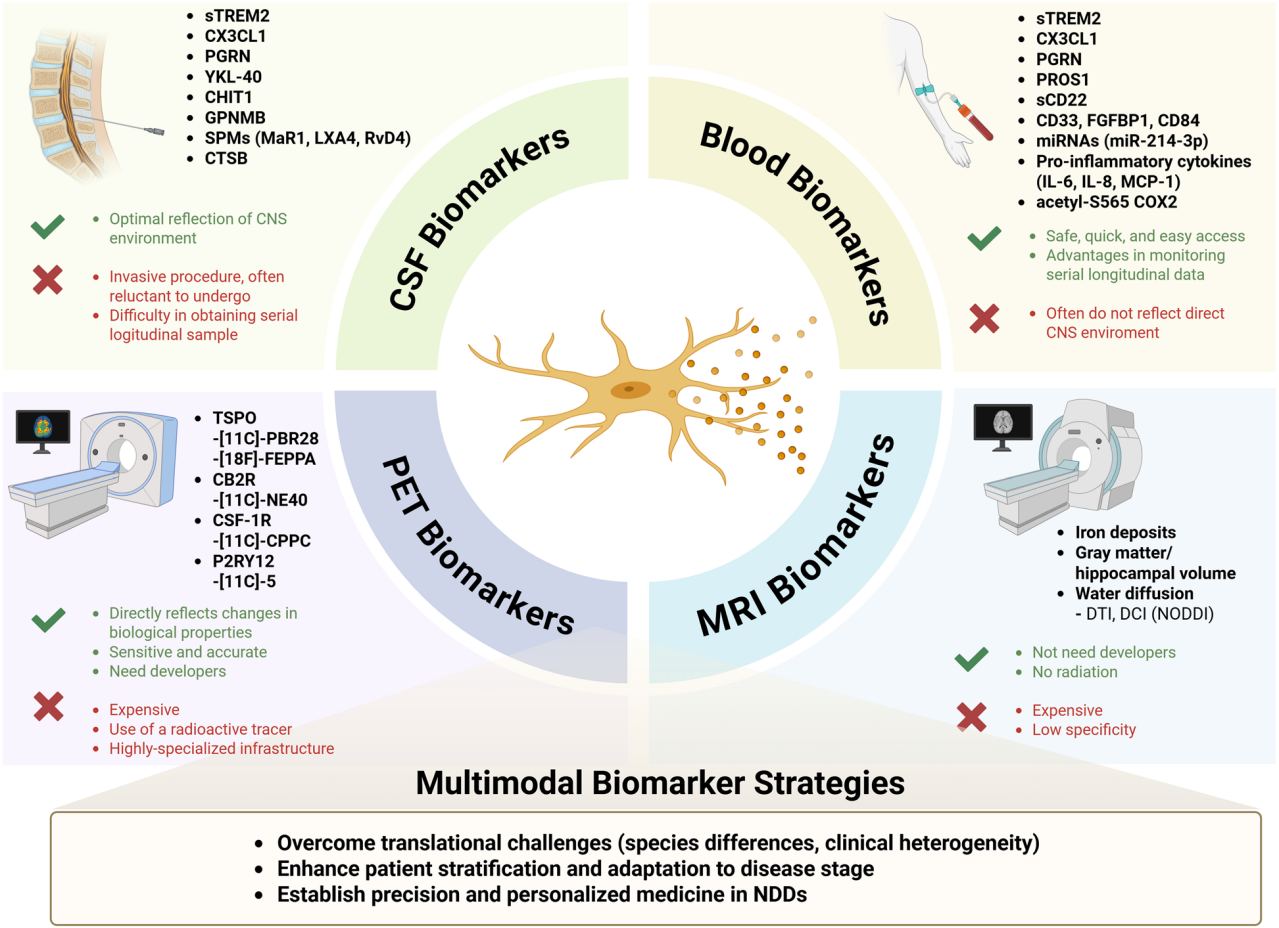
Summary of Fluid and Image Biomarkers for Microglial Functions: Advantages and Limitations. Abbreviations: acetyl-S565 COX2; acetylation of serine 565 residues of cyclooxygenase-2, CAPG; Macrophage-capping protein, CB2R; Cannabinoid receptor type 2, CHIT1; Chitinase-1, CNS; Central nervous system, CSF-1R; Colony stimulating factor-1 receptor, CXCL1; Chemokine (C-X-C motif) ligand 1, DCI; Diffusion compartment imaging, DTI; Diffusion tensor imaging, FABP3; Fatty acid binding protein 3, FGFBP1; Fibroblast growth factor-binding protein 1, GDI1; GDP dissociation inhibitor1, GPNMB; Glycoprotein NMB, IL-6; Interleukin-6, LXA4; Lipoxin A4, MaR 1; Maresin1, MCP-1; Monocyte chemoattractant protein-1, MDH1; Malate dehydrogenase 1, miRNA (miR); microRNA, MRI; Magnetic resonance imaging, P2RY12; Purinergic receptor P2Y12, P2X7R; P2X7 receptor, PET; Positron emission tomography, PGRN; Progranulin, PKCδ; Protein kinase C delta, PROS1; Vitamin K-dependent protein S1, RvD1; Resolvin D1, sCD22; Soluble CD22, SPMs; Specialized pro-resolving mediators, sTREM2; Soluble TREM2,TSPO; Translocator protein, YKL-40; Chitinase 3-like 1

In recent years, substantial progress has been made in developing accessible fluid and imaging biomarkers that reflect the pathological processes of NDDs [296]. Among neuropathology-based biomarkers, reliable assays for amyloid, α-Syn, and TDP-43 are either in development or have already been established. For example, in AD, the ATN classification system—categorizing biomarkers into amyloid (A), tau (T), and neurodegeneration (N)—has provided a useful framework for patient stratification in clinical trials [20]. Furthermore, the Alzheimer’s Association has proposed revised criteria that group fluid biomarkers according to the underlying pathogenic mechanisms [212]. In this framework, Aβ42, phosphorylated tau (p-tau)217, p-tau181, and p-tau231 are considered as biomarkers that become abnormal concurrently with amyloid positron emission tomography (PET) positivity [212]. Regarding tau pathology, tau fragments such as microtubule-binding region containing residue 243 (MTBR-tau243), p-tau205, and non-phosphorylated mid-region tau show strong correlations with tau PET imaging [212, 297]. NfL serves as a marker for neuronal injury, dysfunction, and degeneration, while glial fibrillary acidic protein (GFAP), a marker for astrocytic activation, reflects neuroinflammation [298, 299]. For detecting α-Syn pathology in disorders such as PD or dementia with Lewy bodies (DLB), the α-synuclein seed amplification assay has recently been introduced [300, 301]. In addition, biomarkers reflecting TDP-43 pathology, synaptic dysfunction, and microglial activity are gaining increasing attention [302–305]. In the near future, the development of reliable and disease-specific biomarkers is expected to be instrumental in identifying optimal therapeutic targets, guiding personalized treatment strategies, enabling combinatorial or cocktail approaches, monitoring therapeutic responses, and predicting prognosis Fig. 3.

Although the primary focus of this review is on microglia and microglia-related biomarkers, it is important to first highlight several key biomarkers that have recently garnered attention for their diagnostic, prognostic, and disease-monitoring potential in NDDs. These include plasma Aβ42/40 ratios, various p-tau species, NfL, GFAP, and biomarkers reflecting TDP-43 pathology, such as cryptic exon (CE)–containing proteins, as well as synapse-related markers. After briefly introducing these representative biomarkers, we next discuss emerging CSF and blood-based biomarkers that specifically reflect microglial activation, dysfunction, and their implications for neuroinflammation and disease progression.

#### Various p-tau isoforms

CSF and plasma p-tau217, p-tau181, and p-tau231 can accurately distinguish Aβ-positive from Aβ-negative individuals across both cognitively unimpaired (CU) and cognitively impaired populations [306, 307]. Among these, p-tau217—particularly the percentage ratio of p-tau217 to non-phosphorylated tau (%p-tau217)—has demonstrated superior diagnostic accuracy than other plasma biomarkers, including p-tau181, p-tau231, GFAP, NfL, and Aβ42/40 [308], and has performed comparably or better than FDA-approved CSF assays for Aβ status classification [309]. Adding *ApoE* genotype, NfL, or GFAP to plasma p-tau further improves the accuracy of predicting amyloid positivity or disease prognosis [307, 310]. Plasma p-tau also serves as a prognostic marker, predicting future cognitive decline in individuals with CU or mild cognitive impairment (MCI) [311]. Longitudinal increases in p-tau217 are associated with clinical deterioration and brain atrophy in CU subjects [312]. However, plasma p-tau217, p-tau181, and p-tau231 show stronger correlations with Aβ pathology than tau aggregates [313, 314]. In contrast, CSF p-tau205 and MTBR-tau243 show better correlation with tau PET than amyloid PET imaging [212, 297]. Notably, plasma MTBR-tau243**,** a newly developed endogenously cleaved tau fragment, has shown strong associations with tau PET and closely reflects cognitive status [314].

#### Neurofilaments

Neurofilaments- particularly NfL and phosphorylated neurofilament heavy chain (pNfH)- are among the most promising biomarkers across NDDs and are measurable in both CSF and blood. It reflects axonal degeneration and injury, irrespective of the underlying cause, and is elevated in various NDDs, including AD, ALS, FTD, Huntington’s disease (HD), PD, and related disorders [279, 280, 315, 316]. CSF NfL has been thoroughly evaluated as diagnostic, prognostic, susceptibility/risk, and pharmacodynamic/response biomarkers for NDDs. More recently, the advent of highly sensitive assay technologies has enabled the reliable detection of neurofilaments in blood, providing a less invasive alternative to CSF and facilitating large-scale studies. Especially, NfL has demonstrated utility as a prognostic biomarker in AD [317], PD [273], and ALS [275], with higher levels correlating with faster disease progression and higher rates of brain atrophy [315]. They have also shown promise as pharmacodynamic biomarkers, as demonstrated in clinical trials such as VALOR (NCT02623699) and ATLAS (NCT04856982), to evaluate the efficacy and safety of tofersen, an antisense oligonucleotide (ASO) targeting SOD1, in patients with ALS [257, 318]. Despite their utility, neurofilaments have limitations, including a lack of specificity, variability across assays and cohorts, and potential confounders. The interpretation of blood NfL levels must account for physiological confounders, such as age and body mass index (BMI), as levels increase with age and decrease modestly with higher BMI [319, 320]. In ALS, although NfL and TDP-43 help identify rapidly progressing cases, their clinical sensitivity is limited by genetic and phenotypic heterogeneity [321]. Nevertheless, ongoing efforts to integrate neurofilaments with other biomarkers, such as miRNAs (e.g., miR-214-3p, miR-181), inflammatory markers, and genetic risk profiles, may improve their diagnostic and prognostic precision across the heterogeneous spectrum of NDDs [105, 322].

#### SOD1

SOD1 reduction is a key therapeutic strategy in ALS patients with *SOD1* mutations, as mutant SOD1 misfolding and aggregation are linked to toxicity [291]. Preclinical studies in rodents and non-human primates have demonstrated that SOD1-targeting ASOs reduce CNS SOD1 expression, delay disease onset, and slow progression [258, 259]. These ASOs also lowered CSF SOD1 levels, supporting its use as a pharmacodynamic marker. Clinical evidence from the VALOR trial further confirmed that tofersen administration reduces CSF SOD1, indicating effective target engagement [257].

#### Dipeptide repeat proteins

G_4_C_2_ repeat expansions in the *C9orf72* gene, the most common genetic cause of ALS and FTD, lead to the production of toxic dipeptide repeat proteins (DPRs), including poly(GP), poly(GA), and poly(GR) [292, 293]. Although CSF DPR levels do not correlate with clinical severity, they are detectable in symptomatic carriers of *C9orf72* expansions [260, 323]. Preclinical studies demonstrate that ASOs targeting repeat RNA reduce DPR levels in the brain, spinal cord, and CSF, supporting their use as pharmacodynamic biomarkers [260, 261]. Ongoing clinical trials, such as BIIB078 (NCT03626012), an investigational ASO for *C9orf72*-associated ALS that degrades G_4_C_2_ expansion-containing mRNA, and WVE-004 (NCT04931862), an ASO targeting repeat-containing pre-mRNA variants in patients with c9ALS/FTD, aim to evaluate these effects. Therefore, biomarkers may help assess target engagement in future therapeutic strategies for *C9orf72*-associated ALS/FTD.

#### GFAP

Plasma GFAP, a marker of reactive astrocytes, is considered a plasma biomarker linking amyloid pathology to early tau pathology, as Aβ is associated with p-tau only in individuals with higher GFAP levels [324]. It predicts disease progression in the preclinical stage of AD and PD [269, 325]. GFAP can also be increased in patients with FTD or ALS [233, 326].

#### TDP-43-related biomarkers

TDP-43 pathology—characterized by its nuclear depletion and cytoplasmic aggregation—is a central feature of ALS and FTD [327], and is increasingly recognized in other NDDs, such as LATE and AD [328, 329]. Although measuring total or modified TDP-43 in CSF and plasma has shown some diagnostic potential [330–332], the results have been inconsistent due to variability in detection methods, antibody specificity, and uncertainties regarding the origin and interpretation of TDP-43 in biofluids [332, 333]. To overcome these challenges, recent efforts have focused on biomarkers that reflect TDP-43 functional loss, particularly the aberrant inclusion of CE in transcripts due to impaired splicing regulation caused by nuclear TDP-43 depletion [334]. Recent studies have shown that identified TDP-43-mediated misspliced cryptic transcripts, such as *stathmin 2 (STMN2)*, *UNC13A*, and *hepatoma-derived growth factor-like protein 2 (HDGFL2)*, can produce stable CE-containing novel proteins in some cases [221, 302, 335–337]. One promising direction involves detecting CE–encoded peptides, such as HDGFL2, which are elevated in CSF from both symptomatic and presymptomatic *C9orf72* mutation carriers and may precede neurofilament elevation [302]. CE–encoded peptides reflect early TDP-43 dysfunction and show promise as early diagnostic and prognostic biomarkers in ALS and FTD. Mass spectrometry has identified multiple cryptic peptides in the CSF of affected patients, highlighting their broader biomarker potential [338]. Although further validation is needed to determine their specificity and sensitivity, these findings represent an important step toward enabling earlier diagnosis and monitoring target engagement in clinical trials for therapies aimed at restoring TDP-43 function.

#### Synapse-related biomarkers

Emerging biomarkers of synaptic degeneration include synaptosomal-associated protein 2 A (SV2A), synaptosomal-associated protein 25 (SNAP-25), and growth-associated protein 43 (GAP-43) for pre-synaptic damage, and neurogranin (Ng) for post-synaptic degeneration [230, 339–341]. GAP-43, a presynaptic protein involved in axonal growth and synaptic remodeling, is elevated in AD and correlates with the progression of tau pathology [229, 342]. Ng, a postsynaptic protein critical for memory formation, is predominantly expressed in the cortex, hippocampus, and amygdala—regions vulnerable to AD pathology [343]. Increased CSF Ng levels help differentiate AD from other NDDs such as FTLD [230, 231] and are associated with cognitive decline in AD [232]. SNAP-25, essential for synaptic vesicle fusion and neurotransmitter release, has also been proposed as a CSF biomarker of synaptic dysfunction [228]. Since synaptic loss correlates most closely with functional impairment in NDDs, synaptic proteins such as SNAP-25 and Ng hold strong potential as diagnostic and disease-monitoring biomarkers [344].

### Candidate biomarkers related to microglial function

#### CSF biomarkers

CSF biomarkers provide a direct window into central nervous system processes, although their invasive nature limits their routine clinical application. Several CSF biomarkers related to microglial activity are currently under investigation across various NDDs, yet most require further validation before clinical application.

sTREM2 is produced by the cleavage of the extracellular domain of TREM2 and released into the extracellular space [345]. CSF sTREM2 levels increase during the early symptomatic phase of AD, reflecting changes in microglial activation [78]. This increase occurs after the appearance of amyloid pathology but before the emergence of tau-related biomarkers, particularly in autosomal-dominant AD. Longitudinal studies associate rising CSF sTREM2 levels with baseline amyloid biomarkers rather than tau, supporting its role in early intervention strategies, including TREM2-targeted therapies [346, 347]. sTREM2 also appears to inhibit amyloid fibrillation and is associated with reduced hippocampal atrophy and slower cognitive decline [348, 349]. However, its utility is limited by inter-individual variability and its decoupling from microglial activation in specific contexts, such as treatment with AL002 [77, 349]. CSF sTREM2 levels have also been linked to disease severity and prognosis in PD and ALS [350, 351].

PGRN, a protein associated with microglial function, shows increased CSF levels during AD progression and correlates with sTREM2 and neurodegeneration markers [222]. Elevated CSF PGRN has been linked to cognitive decline in AD [352]. However, some studies report no significant differences in CSF and plasma PGRN levels among patients with MCI, AD, FTD, DLB, or ALS and healthy controls [353, 354]. Chemokine (C-X3-C motif) ligand 1 (CX3CL1/Fractalkine) is a neuronally expressed chemokine that modulates microglial activity. Its CSF levels are reduced in patients with AD dementia compared to controls [355], though elevated levels have been observed in MCI and AD relative to CU individuals [224]. Lower CX3CL1 levels have been associated with tau pathology, suggesting relevance in early disease processes [224].

Secreted by activated microglia and astrocytes, chitinase-3-like protein 1 (CHI3L1/YKL-40) is involved in inflammation and tissue remodeling [356]. Elevated CSF YKL-40 levels are associated with tau pathology, brain atrophy, AD progression, and the transition from MCI to AD dementia [225–227]. It correlates with other markers of neurodegeneration (e.g., total tau, NfL) and synaptic damage (e.g., Ng, SNAP-25) [357]. Elevated levels have also been reported in FTD, but not in vascular dementia or DLB [356].

Chitinase-1 (CHIT1), a marker of innate immune activation, is elevated in the CSF of patients with ALS and MS, correlating with early microglial activation and faster disease progression [247, 248, 358–360]. Glycoprotein NMB (GPNMB), which is partly localized to the cell surface, is elevated in the brains and CSF of patients with late-onset AD [361]. Soluble Aβ induces GPNMB expression in microglia, and increased CSF GPNMB has also been observed in PD [362, 363]. Lipid mediators, such as maresin 1 (MaR1), resolvin D1 (RvD1), and neuroprotectin D1 (NPD1), are diminished in the CSF of AD patients with cognitive impairment [240]. MaR1 enhances the microglial uptake of Aβ and contributes to resolving neuroinflammation [99, 101]. Cathepsin B (CTSB) is a lysosomal protease produced by microglia that is elevated in the plasma and brain of AD patients. It plays a role in amyloid metabolism and neuroinflammation [238, 239].

#### Blood biomarkers

Blood-based biomarkers are less invasive and more practical for screening and longitudinal monitoring in NDDs. Although their development lags behind CSF-based markers, several microglia-related candidates have emerged [105, 211, 241, 364, 365]. PROS1 binds phosphatidylserine on apoptotic cells, activating TAM receptors on phagocytes [366]. In AD mouse models, Aβ exposure stimulates microglia to release PROS1, with elevated serum levels in AD dementia compared to MCI or CU stages [365]. CD22, a negative regulator of microglial phagocytosis, is elevated in plasma from AD patients as soluble CD22 (sCD22) [96]. It negatively correlates with Aβ42 and cognition, and positively correlates with CSF p-tau and PET-detected amyloid burden [211]. CD33 suppresses microglial Aβ uptake. Blood levels of CD33 are increased in early AD/MCI and associated with amyloid pathology and disease progression, although its role in other NDDs remains to be established [241].

miRNAs (e.g., miR-214-3p), targeting the *NCKAP1* gene, have been identified as a marker of impaired microglial phagocytosis in rapidly progressive ALS. Its plasma levels correlate with disease severity, survival, and inflammatory markers such as NfL and CSF cytokines [105]. Longitudinal studies confirm low levels in slow progressors and sustained elevation in rapid progressors. While upregulation of miR-214 has been reported in prodromal PD [367], its role in other NDDs requires further investigation. In addition to these**,** recent studies suggest a neurodegenerative microglial phenotype characterized by reduced acetyl-S565 COX2 levels and decreased SPMs secretion in AD mouse models. Conversely, increased acetyl-S565 COX2 levels are associated with a reactive microglial phenotype that enhances phagocytosis and anti-inflammatory responses [103]. These findings suggest that acetyl-S565 COX2 could regulate microglial functions and serve as a biomarker for therapeutic targeting.

#### Imaging biomarkers for microglial activation and neuroinflammation

Imaging techniques, such as PET and magnetic resonance imaging (MRI), provide valuable tools for assessing microglial activation and neuroinflammation in NDDs.

##### PET imaging

Translocator protein (18 kDa) (TSPO), which is overexpressed in activated microglia and reactive astrocytes, has become a widely recognized biomarker for neuroinflammation [252, 253]. A variety of TSPO-PET radioligands have been developed, with ^11^C-(R)-PK11195 being the most commonly used in clinical studies. However, this first-generation tracer has limitations, including a short half-life and high nonspecific binding [368]. To overcome these issues, second-generation TSPO tracers such as ^11^C-DAA1106, ^11^C-PBR28, ^11^C-PBR06, ^18^F-FEPPA, ^11^C-CLINME, and ^18^F-DPA714 have been introduced. These tracers have been applied in clinical trials across a range of NDDs, including AD, PD, ALS, and FTD [369]. For instance, early-stage AD patients exhibit increased gray matter and hippocampal volumes, which are associated with early microglial activation, as detected via ^11^C-PBR28 PET [370]. Nevertheless, TSPO-PET tracers have limitations due to variations in binding affinity influenced by a single-nucleotide polymorphism (SNP) in the TSPO gene. Third-generation TSPO tracers, such as ^18^F-GE180 and ^11^C-ER176, have been developed to address these genetic influences; however, further research is required to assess their clinical utility [368]. A meta-analysis with first and second generation of TSPO tracers shows that TSPO PET signal in cortical grey matter increases in patients with AD (standardized mean difference [SMD] = 0.693, *P* < 0.001) and other NDDs including ALS, PD, FTD, DLB and HD (SMD = 0.929, *P* < 0.001) compared to controls. Increased TSPO PET signals have also been observed in cortico-limbic regions and thalamus across these conditions [369]. Beyond TSPO, the novel PET tracers are under development. For example, the colony-stimulating factor 1 receptor (CSF1R), which is upregulated in several NDD models, can be imaged using [^11^C]-CPPC, with increased uptake observed in PD patients that correlates with disease severity [371, 372]. The cannabinoid receptor 2 (CB2R), another marker of microglial activation, can be targeted using [^11^C]-NE40 [373, 374]. Additionally, the P2Y12 receptor (P2RY12), a marker of homeostatic microglia, is downregulated during neuroinflammation and can be monitored with [^11^C]−5 [375, 376]. While these PET tracers provide promising insights into microglial dynamics and hold therapeutic potential, most have been evaluated only in preclinical settings. Robust clinical validation is still needed to confirm their translational relevance.

##### MRI

MRI plays an essential role in both patient selection and treatment monitoring, particularly for treatment targeting amyloid pathology and other neurodegenerative processes. For example, MRI is routinely used to screen for contraindications to anti-amyloid agents, including cerebral microhemorrhages, macrohemorrhages, superficial siderosis, vasogenic edema, lacunar infarcts, white matter hyperintensities, amyloid-related angiitis, and cerebral amyloid angiopathy [377]. MRI is also critical for detecting ARIA, thus aiding treatment decisions regarding anti-amyloid therapies [377].

In the context of monitoring microglial function, iron-sensitive MRI techniques have been explored. High-field MRI using gradient echo sequences can detect MR hypointensities that reflect differential iron deposition in the frontal cortex of AD patients compared to controls. These changes correlate with Aβ plaque burden and tau pathology [378, 379]. Microglial iron accumulation increases with aging, potentially impairing autophagy and exacerbating inflammation. Emerging MRI modalities offer novel avenues for detecting microglial activation. Diffusion tensor imaging (DTI) has been used to assess microstructural changes such as axonal degeneration and myelin loss, though it lacks sensitivity for detecting reactive microglia [380]. But it has failed to detect reactive microglia. In contrast, diffusion compartment imaging (DCI)—particularly neurite orientation dispersion and density imaging (NODDI)—has shown promise in preclinical models by detecting changes in microglial density [380, 381]. Longitudinal imaging using DCI has tracked the progression of reactive microglia through extra-axonal diffusion characteristics. These findings suggest that DCI may offer a non-invasive method for monitoring microglial activity, although validation across NDDs remains limited. Further large-scale studies are required to establish the reliability and clinical applicability of microglial imaging. Future research should prioritize the standardization of imaging protocols, the development of more specific tracers, and the integration of imaging with fluid biomarkers and clinical data. These advances will be essential to realize the full potential of imaging biomarkers in precision medicine approaches for NDDs.

#### Translational challenges and technological advances in microglia-targeted biomarker development

Identifying reliable biomarkers that reflect early pathological processes is critical for managing NDDs across their clinical continuum. Microglia-focused biomarker research has become increasingly important in elucidating the complex immune and inflammatory pathways that drive disease progression. Longitudinal biomarker studies, in particular, enable the tracking of dynamic changes during early disease stages and provide insight into treatment responses [382].

Despite growing interest, many microglia-related biomarkers remain limited to preclinical animal studies. Rodent models have been essential in elucidating microglial biology and identifying candidate biomarkers, but they fall short in replicating the genetic and phenotypic heterogeneity observed in human NDDs [383, 384]. Furthermore, species-specific differences in microglial responses—including the so-called microglial “sensome”—further complicate translation [384, 385]. Emerging chimeric mouse models engrafted with human iPSC-derived microglia offer a promising solution to bridge this translational gap [384].

Advances in single-cell transcriptomics, spatial profiling, and AI–based multi-omics integration are reshaping the landscape of microglia-targeted biomarker and therapeutic development. Single-cell RNA sequencing has enabled the identification of disease-associated microglial states and subpopulations [386], while spatial transcriptomics adds anatomical context to these findings [387, 388]. The AI-driven integration of fluid biomarkers, neuroimaging, and polygenic risk scores has enhanced the classification of NDDs and the prediction of disease trajectories [389–391].

To improve the clinical translation of rodent microglial research, cross-species validation is essential. Platforms such as iPSC- or monocyte-derived human microglia, ex vivo human brain tissue, and humanized mouse models help narrow the translational gap [392–398]. Integrating cross-species multi-omics data, especially through AI-enabled analytics, facilitates the identification of conserved, functionally relevant biomarkers [398, 399]. Incorporating spatial and longitudinal analyses enhances the biological validity and clinical utility of these findings [400]. Moreover, predictive models that integrate clinical biomarkers, neuroimaging, and genomic risk profiles from AD, PD, and ALS cohorts further refine translational pipelines [401–403].

Despite these advances, several key challenges continue to hinder the clinical translation of microglia-targeted biomarkers. First, rigorous standardization and validation across large and diverse patient cohorts are essential to ensure the sensitivity, specificity, and reproducibility of candidate biomarkers. Second, the substantial clinical and biological heterogeneity observed in NDDs complicates the direct application of preclinical findings to real-world patient care. Lastly, no single biomarker modality can comprehensively fulfill diagnostic, prognostic, and therapeutic monitoring requirements. Thus, the integration of fluid, imaging, and genetic biomarkers—supported by advanced computational tools such as machine learning—is critical to fully capture the complexity of neurodegenerative disease processes.

## Conclusions and future directions

Microglial dysfunction is now widely acknowledged as a key driver in the pathophysiology of various NDDs. Recent advances in biomarker discovery and validation offer new avenues for personalized treatment strategies and stratified clinical trials. However, the heterogeneous nature and complexity of NDDs pose ongoing challenges to the development of reliable, clinically actionable biomarkers [404, 405].

In AD, for example, the addition of GFAP and *ApoE* genotype to plasma p-tau217 significantly improved the prediction of amyloid positivity [307], while cognitive metrics further enhanced the prediction of progression to dementia [406]. The effect of anti-amyloid therapy (e.g., donanemab) is greater in patients with low to intermediate tau pathology on PET imaging [263]. Similarly, astrocyte activation, measured via plasma GFAP, may serve as a link between amyloid burden and tau pathology [324].

In Lewy body diseases, multimodal biomarker integration—including myocardial ^18^F-dopamine PET, olfactory testing, and synuclein–tyrosine hydroxylase co-localization—can distinguish synucleinopathy-positive from -negative patients [407]. ^18^F-dopamine PET abnormalities also help predict conversion to Lewy body disorders [408]. In ALS, a combination of miR-181 and NfL levels has been shown to predict prognosis and mortality [322], while plasma miR-214 levels correlate with disease severity and rate of progression [105]. Notably, the impact of microglial dysfunction appears to vary by disease stage and subtype [409, 410]. Co-pathologies are frequent in NDDs and are associated with poorer outcomes. For instance, TDP-43 pathology is found in up to 57% of AD patients, synucleinopathy in ~ 30%, and cerebral amyloid angiopathy in ~ 25% [329, 411]. Such overlapping pathologies necessitate the use of composite biomarker panels to improve prognostication [212, 329, 412].

The future of NDDs research and therapy will likely rely on the integration of multimodal biomarkers, combining fluid, imaging, genetic, and clinical data tailored to each patient’s disease trajectory [413–415]. The success of companion diagnostics (CDx) in oncology offers a compelling model [416]. However, the dynamic, stage-dependent nature of NDDs—along with the frequent presence of co-pathologies—limits the utility of rigid CDx frameworks alone [417, 418].

To advance precision medicine in neurodegeneration, future strategies must employ flexible, combinatory biomarker approaches capable of capturing the spatial, temporal, and molecular heterogeneity of disease. These integrative approaches will enable more personalized and adaptive interventions, ultimately improving patient outcomes and guiding the development of next-generation therapeutics.

## Supplementary Information

Additional file 1.

## Abbreviations

AAV: Adeno-associated virus
acetyl-S565 COX2: Acetylation of serine 565 residues of cyclooxygenase-2
AD: Alzheimer’s disease
AI: Artificial intelligence
ALS: Amyotrophic lateral sclerosis
ApoE: Apolipoprotein E
ARIA: Amyloid-related imaging abnormalities
ASC: Apoptosis-associated speck-like protein containing a caspase recruitment domain
ASO: Antisense oligonucleotide
Aβ: Amyloid beta
BBB: Blood–brain barrier
BMI: Body mass index
C1q: Complement component 1q
C9orf72: Chromosome 9 open reading frame 72
CB2R: Cannabinoid receptor type 2
CDx: Companion diagnostics
CE: Cryptic exons
CHIT1: Chitinase-1
CNS: Central nervous system
CSF: Cerebrospinal fluid
CSF1R: Colony stimulating factor 1 receptor
CTSB: Cathepsin B
CU: Cognitively unimpaired
CXCL1: Chemokine (C-X-C motif) ligand 1
DAM: Disease-associated microglia
DCI: Diffusion compartment imaging
DPRs: Dipeptide repeat proteins
DLB: Dementia with lewy bodies
DTI: Diffusion tensor imaging
EVs: Extracellular vesicles
FcRs: Fc receptors
FcγR: Fc-gamma receptors
FDA: U.S. Food and Drug Administration
FTD: Frontotemporal dementia
FTLD: Frontotemporal lobar degeneration
GAP-43: Growth-associated protein 43
GFAP: Glial fibrillary acidic protein
GPNMB: Glycoprotein NMB
Gas6: Growth arrest-specific 6
GWAS: Genome-wide association studies
H4K12la: Histone H4 lysine 12 lactylation
HCAR2: Hydroxycarboxylic acid receptor 2
HD: Huntington’s disease
HDGFL2: Hepatoma-derived growth factor-like protein 2
HIF: Hypoxia-inducible factor
HK2: Hexokinase 2
iPSC: Induced pluripotent stem cell
IFN-γ: Interferon-gamma
IL: Interleukin
LDs: Lipid droplets
LDAM: Lipid-droplet associated microglia
LRRK2: Leucine-rich repeat kinase 2
mAb: Monoclonal antibody
MaR1: Maresin 1
MCI: Mild cognitive impairment
MCP-1: Monocyte chemoattractant protein-1
MGnD: Neurodegenerative microglia
miRNA: MicroRNA
MRI: Magnetic resonance imaging
MS: Multiple sclerosis
N-AS: N-acetyl sphingosine
NCKAP1: NCK Associated protein 1
NDDs: Neurodegenerative diseases
NfL: Neurofilament light
NF-κB: Nuclear factor kappa-B
Ng: Neurogranin
NLRP3: NOD-like receptor family pyrin domain containing 3
P2RY12: Purinergic receptor P2Y12
PD: Parkinson’s disease
PET: Positron emission tomography
PGE2: Prostaglandin E2
PGRN: Progranulin
Piezo 1: Piezo type mechanosensitive ion channel component 1
PKM2: Pyruvate kinase M2
pNfH: Phosphorylated neurofilament heavy chain
PROS1: Protein S
RIPK1: Receptor-interacting serine/threonine-protein kinase 1
ROS: Reactive oxygen species
RvD1: Resolvin D1
RvE1: Resolvin E1
SMD: Standardized mean difference
SNAP-25: Synaptosomal-associated protein 25
SNP: Single-nucleotide polymorphism
SOD1: Superoxide dismutase 1
SORT1: Sortilin
SphK1: Sphingosine kinase 1
SPMs: Specialized pro-resolving mediators
sTREM2: Soluble TREM2
SYK: Spleen tyrosine kinase
TfR: Transferrin receptor
TAM Receptors: Tyro3, Axl, and MerTK
TDP-43: TAR DNA-binding protein 43
TREM2: Triggering receptor expressed on myeloid cells 2
TLR: Toll-like receptor
TSPO: Translocator protein (18 kDa)
WAM: White matter-associated microglia
α-Syn: Alpha-synuclein

## References

[1] Ransohoff RM, Brown MA. Innate immunity in the central nervous system. J Clin Invest. 2012;122:1164–71. 22466658 10.1172/JCI58644 PMC3314450

[2] Alvarez JI, Katayama T, Prat A. Glial influence on the blood brain barrier. Glia. 2013;61:1939–58. 24123158 10.1002/glia.22575 PMC4068281

[3] Allen NJ, Lyons DA. Glia as architects of central nervous system formation and function. Science. 2018;362:181–5. 30309945 10.1126/science.aat0473 PMC6292669

[4] Gao C, Jiang J, Tan Y, Chen S. Microglia in neurodegenerative diseases: mechanism and potential therapeutic targets. Signal Transduct Target Ther. 2023;8:359. 37735487 10.1038/s41392-023-01588-0 PMC10514343

[5] Galloway DA, Phillips AEM, Owen DRJ, Moore CS. Phagocytosis in the Brain: Homeostasis and Disease. Front Immunol. 2019;10:790. 31040847 10.3389/fimmu.2019.00790 PMC6477030

[6] Paolicelli RC, Sierra A, Stevens B, Tremblay ME, Aguzzi A, Ajami B, Amit I, Audinat E, Bechmann I, Bennett M, et al. Microglia states and nomenclature: A field at its crossroads. Neuron. 2022;110:3458–83. 36327895 10.1016/j.neuron.2022.10.020 PMC9999291

[7] Ransohoff RM. A polarizing question: do M1 and M2 microglia exist? Nat Neurosci. 2016;19:987–91. 27459405 10.1038/nn.4338

[8] Stratoulias V, Venero JL, Tremblay ME, Joseph B. Microglial subtypes: diversity within the microglial community. EMBO J. 2019;38: e101997. 31373067 10.15252/embj.2019101997 PMC6717890

[9] Kwon MS. Advanced therapeutic strategies targeting microglia: beyond neuroinflammation. Arch Pharm Res. 2022;45:618–30. 36166145 10.1007/s12272-022-01406-1

[10] Marschallinger J, Iram T, Zardeneta M, Lee SE, Lehallier B, Haney MS, Pluvinage JV, Mathur V, Hahn O, Morgens DW, et al. Lipid-droplet-accumulating microglia represent a dysfunctional and proinflammatory state in the aging brain. Nat Neurosci. 2020;23:194–208. 31959936 10.1038/s41593-019-0566-1 PMC7595134

[11] Guerreiro R, Wojtas A, Bras J, Carrasquillo M, Rogaeva E, Majounie E, Cruchaga C, Sassi C, Kauwe JS, Younkin S, et al. TREM2 variants in Alzheimer’s disease. N Engl J Med. 2013;368:117–27. 23150934 10.1056/NEJMoa1211851 PMC3631573

[12] Colonna M. The biology of TREM receptors. Nat Rev Immunol. 2023;23:580–94. 36750615 10.1038/s41577-023-00837-1 PMC9904274

[13] Xie M, Liu YU, Zhao S, Zhang L, Bosco DB, Pang YP, Zhong J, Sheth U, Martens YA, Zhao N, et al. TREM2 interacts with TDP-43 and mediates microglial neuroprotection against TDP-43-related neurodegeneration. Nat Neurosci. 2022;25:26–38. 34916658 10.1038/s41593-021-00975-6 PMC8741737

[14] Guo Y, Wei X, Yan H, Qin Y, Yan S, Liu J, Zhao Y, Jiang F, Lou H. TREM2 deficiency aggravates alpha-synuclein-induced neurodegeneration and neuroinflammation in Parkinson’s disease models. FASEB J. 2019;33:12164–74. 31370707 10.1096/fj.201900992R PMC6902667

[15] Li XX, Zhang F. Targeting TREM2 for Parkinson’s Disease: Where to Go? Front Immunol. 2021;12: 795036. 35003116 10.3389/fimmu.2021.795036 PMC8740229

[16] Loeffler DA. Antibody-Mediated Clearance of Brain Amyloid-beta: Mechanisms of Action, Effects of Natural and Monoclonal Anti-Abeta Antibodies, and Downstream Effects. J Alzheimers Dis Rep. 2023;7:873–99. 37662616 10.3233/ADR-230025 PMC10473157

[17] Salloway S, Chalkias S, Barkhof F, Burkett P, Barakos J, Purcell D, Suhy J, Forrestal F, Tian Y, Umans K, et al. Amyloid-Related Imaging Abnormalities in 2 Phase 3 Studies Evaluating Aducanumab in Patients With Early Alzheimer Disease. JAMA Neurol. 2022;79:13–21. 34807243 10.1001/jamaneurol.2021.4161 PMC8609465

[18] Honig LS, Barakos J, Dhadda S, Kanekiyo M, Reyderman L, Irizarry M, Kramer LD, Swanson CJ, Sabbagh M. ARIA in patients treated with lecanemab (BAN2401) in a phase 2 study in early Alzheimer’s disease. Alzheimers Dement (N Y). 2023;9: e12377. 36949897 10.1002/trc2.12377 PMC10026083

[19] Loomis SJ, Miller R, Castrillo-Viguera C, Umans K, Cheng W, O’Gorman J, Hughes R, Budd Haeberlein S, Whelan CD. Genome-Wide Association Studies of ARIA From the Aducanumab Phase 3 ENGAGE and EMERGE Studies. Neurology. 2024;102: e207919. 38165296 10.1212/WNL.0000000000207919 PMC11097767

[20] Jack CR Jr, Bennett DA, Blennow K, Carrillo MC, Dunn B, Haeberlein SB, Holtzman DM, Jagust W, Jessen F, Karlawish J, et al. NIA-AA Research Framework: Toward a biological definition of Alzheimer’s disease. Alzheimers Dement. 2018;14:535–62. 29653606 10.1016/j.jalz.2018.02.018 PMC5958625

[21] Jack CR Jr, Bennett DA, Blennow K, Carrillo MC, Feldman HH, Frisoni GB, Hampel H, Jagust WJ, Johnson KA, Knopman DS, et al. A/T/N: An unbiased descriptive classification scheme for Alzheimer disease biomarkers. Neurology. 2016;87:539–47. 27371494 10.1212/WNL.0000000000002923 PMC4970664

[22] Paul R, Jackson S, Ward M, Joshi A, Castro A, Yeh FL, Liao Y, Morrison G. INVOKE-2: A phase 2 randomized, double-blind, placebo-controlled study to evaluate the efficacy and safety of AL002 in participants with early Alzheimer’s disease. Alzheimer’s & Dementia. 2021;17: e054615.

[23] Baseline Characteristics for INVOKE-2: A Phase 2 Randomized, Double-Blind, Placebo-Controlled Study Evaluating AL002 in Early Alzheimer’s Disease. https://investors.alector.com/static-files/2f05cf85-8deb-4755-9cb8-6e6a0f8e3d04.

[24] Martin Stangel DF, Derya Shimshek, Fabrizio Gasparini, Ivan Galimberti, Nathalie George, Gisela Peraus, and Judit Sovago: VHB937, a TREM2 Stabilizing and Activating Antibody Strongly Reduces Pathology After Peripheral Administration in a Broad Range of Animal Models for Neuroinflammation and Neurodegeneration (P4–4.004). Neurology 2024.

[25] Christian Mirescu: Vigil Announces Oral Presentation on Small Molecule TREM2 Agonist VG-3927 as a Potential Disease-Modifying Therapeutic at AD/PD 2024. https://investors.vigilneuro.com/news-releases/news-release-details/vigil-announces-oral-presentation-small-molecule-trem2-agonist.

[26] Maslyar D, Paul R, Long H, Rhinn H, Tassi I, Morrison G, Yeh F, Schwabe T, Ward M. A Phase 1 Study of AL003 in Healthy Volunteers and Participants with Alzheimer’s disease (P5–3.002). Neurology. 2022;98:3582.

[27] J.Y.C. Chan YGH, 1 C.M. Powell,1 D.L. Cooper1: ASPIRE-FTD: A Phase 1/2 Clinical Trial to Evaluate AVB-101 in FTD with GRN mutations (FTD-GRN). ISFTD, Amsterdam, 2024 (https://aviadobio.com/wp-content/uploads/2024/09/ISFTD-2024_AVB-Trial-Design_DIGITAL_FINAL_04Sep2024-1.pdf).

[28] Logan T, Simon MJ, Rana A, Cherf GM, Srivastava A, Davis SS, Low RLY, Chiu CL, Fang M, Huang F, et al. Rescue of a lysosomal storage disorder caused by Grn loss of function with a brain penetrant progranulin biologic. Cell. 2021;184(4651–4668): e4625. 10.1016/j.cell.2021.08.002 PMC8489356 34450028

[29] Amy Chang Berger IC, Mohammad Jafarnejad, Chi-Lu Chiu, Akhil Bhalla, Lorna Damo, Niraj M Shanbhag, Arthur Simen, Hong Lu, Stephen Zicha, Martin Bednar, Matthew D. Troyer, Carole Ho, Richard Tsai: Safety and pharmacokinetics of single ascending doses ofTAK-594/DNL593, a brain-penetrant progranulin replacementtherapy, in healthy volunteers: Interim results from Part A of aPhase 1/2 clinical trial. *Alzheimer’s & DementiaVolume 19: Drug Development* 2023.

[30] Kurnellas M, Mitra A, Schwabe T, Paul R, Arrant AE, Roberson ED, Ward M, Yeh F, Long H, Rosenthal A. Latozinemab, a novel progranulin-elevating therapy for frontotemporal dementia. J Transl Med. 2023;21:387. 37322482 10.1186/s12967-023-04251-y PMC10268535

[31] Ward M, Carter LP, Huang JY, Maslyar D, Budda B, Paul R, Rosenthal A. Phase 1 study of latozinemab in progranulin-associated frontotemporal dementia. Alzheimers Dement (N Y). 2024;10: e12452. 38356474 10.1002/trc2.12452 PMC10865485

[32] Alector I: Alector Presents Baseline Characteristics for Pivotal INFRONT-3 Phase 3 Clinical Trial.* ISFTD 2024, Amsterdam, The Netherlands.*

[33] Rosenberg JB, Kaplitt MG, De BP, Chen A, Flagiello T, Salami C, Pey E, Zhao L, Ricart Arbona RJ, Monette S, et al. AAVrh.10-Mediated APOE2 Central Nervous System Gene Therapy for APOE4-Associated Alzheimer’s Disease. Hum Gene Ther Clin Dev. 2018;29:24–47. 10.1089/humc.2017.231 PMC5870071 29409358

[34] Turner RS, Hebron ML, Lawler A, Mundel EE, Yusuf N, Starr JN, Anjum M, Pagan F, Torres-Yaghi Y, Shi W, et al. Nilotinib Effects on Safety, Tolerability, and Biomarkers in Alzheimer’s Disease. Ann Neurol. 2020;88:183–94. 32468646 10.1002/ana.25775 PMC7383852

[35] Gordon ML, Christen E, Keehlisen L, Gong M, Lam F, Giliberto L, Gomar JJ, Koppel J. An Open-Label, Pilot Study of Daratumumab SC in Mild to Moderate Alzheimer’s Disease. J Alzheimers Dis Rep. 2024;8:1111–4. 39114556 10.3233/ADR-240089 PMC11305838

[36] Genge A, van den Berg LH, Frick G, Han S, Abikoff C, Simmons A, Lin Q, Patra K, Kupperman E, Berry JD. Efficacy and Safety of Ravulizumab, a Complement C5 Inhibitor, in Adults With Amyotrophic Lateral Sclerosis: A Randomized Clinical Trial. JAMA Neurol. 2023;80:1089–97. 37695623 10.1001/jamaneurol.2023.2851 PMC10495927

[37] McGarry A, Rosanbalm S, Leinonen M, Olanow CW, To D, Bell A, Lee D, Chang J, Dubow J, Dhall R, et al. Safety, tolerability, and efficacy of NLY01 in early untreated Parkinson’s disease: a randomised, double-blind, placebo-controlled trial. Lancet Neurol. 2024;23:37–45. 38101901 10.1016/S1474-4422(23)00378-2

[38] Vijiaratnam N, Girges C, Auld G, Chau M, Maclagan K, King A, Skene S, Chowdhury K, Hibbert S, Morris H, et al. Exenatide once weekly over 2 years as a potential disease-modifying treatment for Parkinson’s disease: protocol for a multicentre, randomised, double blind, parallel group, placebo controlled, phase 3 trial: The ‘Exenatide-PD3’ study. BMJ Open. 2021;11: e047993. 34049922 10.1136/bmjopen-2020-047993 PMC8166598

[39] Potter H, Woodcock JH, Boyd TD, Coughlan CM, O’Shaughnessy JR, Borges MT, Thaker AA, Raj BA, Adamszuk K, Scott D, et al. Safety and efficacy of sargramostim (GM-CSF) in the treatment of Alzheimer’s disease. Alzheimers Dement (N Y). 2021;7: e12158. 33778150 10.1002/trc2.12158 PMC7988877

[40] Muller T. DNL151, DNL201, and BIIB094: experimental agents for the treatment of Parkinson’s disease. Expert Opin Investig Drugs. 2023;32:787–92. 37755071 10.1080/13543784.2023.2263357

[41] Zhao HT, John N, Delic V, Ikeda-Lee K, Kim A, Weihofen A, Swayze EE, Kordasiewicz HB, West AB, Volpicelli-Daley LA. LRRK2 Antisense Oligonucleotides Ameliorate alpha-Synuclein Inclusion Formation in a Parkinson’s Disease Mouse Model. Mol Ther Nucleic Acids. 2017;8:508–19. 28918051 10.1016/j.omtn.2017.08.002 PMC5573879

[42] Reading CL, Ahlem CN, Murphy MF. NM101 Phase III study of NE3107 in Alzheimer’s disease: rationale, design and therapeutic modulation of neuroinflammation and insulin resistance. Neurodegener Dis Manag. 2021;11:289–98. 34251287 10.2217/nmt-2021-0022

[43] Anti-inflammatory Intervention with dapansutrile (OLT1177) for PD modification (DAPA-PD) (https://www.isrctn.com/ISRCTN16806940).

[44] Patricia Inácio P. 1st patient dosed in Phase 2 trial of Parkinson’s therapy VTX3232. SAN DIEGO, June 17, 2025 (https://ir.ventyxbio.com/node/9471/pdf).

[45] Amo-Aparicio J, Daly J, Hojen JF, Dinarello CA. Pharmacologic inhibition of NLRP3 reduces the levels of alpha-synuclein and protects dopaminergic neurons in a model of Parkinson’s disease. J Neuroinflammation. 2023;20:147. 37349821 10.1186/s12974-023-02830-w PMC10286423

[46] Marisa Wexler M: Zydus launches Phase 2 trial of anti-inflammatory ZYIL1. Ahmedabad, India, October 25, 2023.

[47] Tormahlen NM, Martorelli M, Kuhn A, Maier F, Guezguez J, Burnet M, Albrecht W, Laufer SA, Koch P. Design and Synthesis of Highly Selective Brain Penetrant p38alpha Mitogen-Activated Protein Kinase Inhibitors. J Med Chem. 2022;65:1225–42. 33974419 10.1021/acs.jmedchem.0c01773

[48] Baruch K, Kertser A, Matalon O, Forsht O, Braiman S, Shochat E, David C, Yoles E. IBC-Ab002, an anti-PD-L1 monoclonal antibody tailored for treating Alzheimer’s disease: Nonhuman/Lead optimization studies. Alzheimer’s & Dementia. 2020;16: e042978.

[49] Vissers M, Heuberger J, Groeneveld GJ, Oude Nijhuis J, De Deyn PP, Hadi S, Harris J, Tsai RM, Cruz-Herranz A, Huang F, et al. Safety, pharmacokinetics and target engagement of novel RIPK1 inhibitor SAR443060 (DNL747) for neurodegenerative disorders: Randomized, placebo-controlled, double-blind phase I/Ib studies in healthy subjects and patients. Clin Transl Sci. 2022;15:2010–23. 35649245 10.1111/cts.13317 PMC9372423

[50] Hincelin-Mery A, Nicolas X, Cantalloube C, Pomponio R, Lewanczyk P, Benamor M, Ofengeim D, Krupka E, Hsiao-Nakamoto J, Eastenson A, Atassi N. Safety, pharmacokinetics, and target engagement of a brain penetrant RIPK1 inhibitor, SAR443820 (DNL788), in healthy adult participants. Clin Transl Sci. 2024;17: e13690. 38010108 10.1111/cts.13690 PMC10772668

[51] Guerrero A, De Strooper B, Arancibia-Carcamo IL. Cellular senescence at the crossroads of inflammation and Alzheimer’s disease. Trends Neurosci. 2021;44:714–27. 34366147 10.1016/j.tins.2021.06.007

[52] Garbarino VR, Palavicini JP, Melendez J, Barthelemy N, He Y, Kautz TF, Lopez-Cruzan M, Mathews JJ, Xu P, Zhan B, et al. Evaluation of Exploratory Fluid Biomarker Results from a Phase 1 Senolytic Trial in Mild Alzheimer’s Disease. Preprint at 10.21203/rs.3.rs-3994894/v1 (Res Sq. 2024)10.1016/j.neurot.2025.e0059140274471

[53] Gonzales MM, Krishnamurthy S, Garbarino V, Daeihagh AS, Gillispie GJ, Deep G, Craft S, Orr ME. A geroscience motivated approach to treat Alzheimer’s disease: Senolytics move to clinical trials. Mech Ageing Dev. 2021;200: 111589. 34687726 10.1016/j.mad.2021.111589 PMC9059898

[54] Zammit M, Tao Y, Olsen ME, Metzger J, Vermilyea SC, Bjornson K, Slesarev M, Block WF, Fuchs K, Phillips S, et al. [(18)F]FEPPA PET imaging for monitoring CD68-positive microglia/macrophage neuroinflammation in nonhuman primates. EJNMMI Res. 2020;10:93. 32761399 10.1186/s13550-020-00683-5 PMC7410886

[55] Zurcher NR, Loggia ML, Mullett JE, Tseng C, Bhanot A, Richey L, Hightower BG, Wu C, Parmar AJ, Butterfield RI, et al. [(11)C]PBR28 MR-PET imaging reveals lower regional brain expression of translocator protein (TSPO) in young adult males with autism spectrum disorder. Mol Psychiatry. 2021;26:1659–69. 32076115 10.1038/s41380-020-0682-z PMC8159742

[56] Dubois B, Lopez-Arrieta J, Lipschitz S, Doskas T, Spiru L, Moroz S, Venger O, Vermersch P, Moussy A, Mansfield CD, et al. Masitinib for mild-to-moderate Alzheimer’s disease: results from a randomized, placebo-controlled, phase 3, clinical trial. Alzheimers Res Ther. 2023;15:39. 36849969 10.1186/s13195-023-01169-x PMC9972756

[57] Latham BD, Oskin DS, Crouch RD, Vergne MJ, Jackson KD. Cytochromes P450 2C8 and 3A Catalyze the Metabolic Activation of the Tyrosine Kinase Inhibitor Masitinib. Chem Res Toxicol. 2022;35:1467–81. 36048877 10.1021/acs.chemrestox.2c00057 PMC10226528

[58] Lim SM, Nahm M, Kim SH. Proteostasis and Ribostasis Impairment as Common Cell Death Mechanisms in Neurodegenerative Diseases. J Clin Neurol. 2023;19:101–14. 36854331 10.3988/jcn.2022.0379 PMC9982182

[59] O’Rourke JG, Bogdanik L, Yanez A, Lall D, Wolf AJ, Muhammad AK, Ho R, Carmona S, Vit JP, Zarrow J, et al. C9orf72 is required for proper macrophage and microglial function in mice. Science. 2016;351:1324–9. 26989253 10.1126/science.aaf1064 PMC5120541

[60] Zhao Y, Wu X, Li X, Jiang LL, Gui X, Liu Y, Sun Y, Zhu B, Pina-Crespo JC, Zhang M, et al. TREM2 Is a Receptor for beta-Amyloid that Mediates Microglial Function. Neuron. 2018;97(1023–1031): e1027. 10.1016/j.neuron.2018.01.031 PMC5889092 29518356

[61] Filipello F, Morini R, Corradini I, Zerbi V, Canzi A, Michalski B, Erreni M, Markicevic M, Starvaggi-Cucuzza C, Otero K, et al. The Microglial Innate Immune Receptor TREM2 Is Required for Synapse Elimination and Normal Brain Connectivity. Immunity. 2018;48(979–991): e978. 10.1016/j.immuni.2018.04.016 29752066

[62] Cady J, Koval ED, Benitez BA, Zaidman C, Jockel-Balsarotti J, Allred P, Baloh RH, Ravits J, Simpson E, Appel SH, et al. TREM2 variant p.R47H as a risk factor for sporadic amyotrophic lateral sclerosis. JAMA Neurol. 2014;71:449–53. 24535663 10.1001/jamaneurol.2013.6237 PMC4087113

[63] Rikos D, Siokas V, Aloizou AM, Tsouris Z, Aslanidou P, Koutsis G, Anagnostouli M, Bogdanos DP, Grigoriadis N, Hadjigeorgiou GM, Dardiotis E. TREM2 R47H (rs75932628) variant is unlikely to contribute to Multiple Sclerosis susceptibility and severity in a large Greek MS cohort. Mult Scler Relat Disord. 2019;35:116–8. 31362167 10.1016/j.msard.2019.07.007

[64] Peplonska B, Berdynski M, Mandecka M, Barczak A, Kuzma-Kozakiewicz M, Barcikowska M, Zekanowski C. TREM2 variants in neurodegenerative disorders in the Polish population. Homozygosity and compound heterozygosity in FTD patients. Amyotroph Lateral Scler Frontotemporal Degener. 2018;19:407–12. 29557178 10.1080/21678421.2018.1451894

[65] Dardiotis E, Rikos D, Siokas V, Aloizou AM, Tsouris Z, Sakalakis E, Brotis AG, Bogdanos DP, Hadjigeorgiou GM. Assessment of TREM2 rs75932628 variant’s association with Parkinson’s disease in a Greek population and Meta-analysis of current data. Int J Neurosci. 2021;131:544–8. 32250197 10.1080/00207454.2020.1750388

[66] Jay TR, von Saucken VE, Landreth GE. TREM2 in Neurodegenerative Diseases. Mol Neurodegener. 2017;12:56. 28768545 10.1186/s13024-017-0197-5 PMC5541421

[67] Jin SC, Benitez BA, Karch CM, Cooper B, Skorupa T, Carrell D, Norton JB, Hsu S, Harari O, Cai Y, et al. Coding variants in TREM2 increase risk for Alzheimer’s disease. Hum Mol Genet. 2014;23:5838–46. 24899047 10.1093/hmg/ddu277 PMC4189899

[68] Xiang X, Werner G, Bohrmann B, Liesz A, Mazaheri F, Capell A, Feederle R, Knuesel I, Kleinberger G, Haass C. TREM2 deficiency reduces the efficacy of immunotherapeutic amyloid clearance. EMBO Mol Med. 2016;8:992–1004. 27402340 10.15252/emmm.201606370 PMC5009806

[69] Lee SH, Meilandt WJ, Xie L, Gandham VD, Ngu H, Barck KH, Rezzonico MG, Imperio J, Lalehzadeh G, Huntley MA, et al. Trem2 restrains the enhancement of tau accumulation and neurodegeneration by beta-amyloid pathology. Neuron. 2021;109(1283–1301): e1286. 10.1016/j.neuron.2021.02.010 33675684

[70] Li Y, Xu H, Wang H, Yang K, Luan J, Wang S. TREM2: Potential therapeutic targeting of microglia for Alzheimer’s disease. Biomed Pharmacother. 2023;165: 115218. 37517293 10.1016/j.biopha.2023.115218

[71] Bemiller SM, McCray TJ, Allan K, Formica SV, Xu G, Wilson G, Kokiko-Cochran ON, Crish SD, Lasagna-Reeves CA, Ransohoff RM, et al. TREM2 deficiency exacerbates tau pathology through dysregulated kinase signaling in a mouse model of tauopathy. Mol Neurodegener. 2017;12:74. 29037207 10.1186/s13024-017-0216-6 PMC5644120

[72] Leyns CEG, Ulrich JD, Finn MB, Stewart FR, Koscal LJ, Remolina Serrano J, Robinson GO, Anderson E, Colonna M, Holtzman DM. TREM2 deficiency attenuates neuroinflammation and protects against neurodegeneration in a mouse model of tauopathy. Proc Natl Acad Sci U S A. 2017;114:11524–9. 29073081 10.1073/pnas.1710311114 PMC5663386

[73] Wang Y, Cella M, Mallinson K, Ulrich JD, Young KL, Robinette ML, Gilfillan S, Krishnan GM, Sudhakar S, Zinselmeyer BH, et al. TREM2 lipid sensing sustains the microglial response in an Alzheimer’s disease model. Cell. 2015;160:1061–71. 25728668 10.1016/j.cell.2015.01.049 PMC4477963

[74] Nugent AA, Lin K, van Lengerich B, Lianoglou S, Przybyla L, Davis SS, Llapashtica C, Wang J, Kim DJ, Xia D, et al. TREM2 Regulates Microglial Cholesterol Metabolism upon Chronic Phagocytic Challenge. Neuron. 2020;105(837–854): e839. 10.1016/j.neuron.2019.12.007 31902528

[75] Rachmian N, Medina S, Cherqui U, Akiva H, Deitch D, Edilbi D, Croese T, Salame TM, Ramos JMP, Cahalon L, et al. Identification of senescent, TREM2-expressing microglia in aging and Alzheimer’s disease model mouse brain. Nat Neurosci. 2024;27:1116–24. 38637622 10.1038/s41593-024-01620-8

[76] Wang S, Mustafa M, Yuede CM, Salazar SV, Kong P, Long H, Ward M, Siddiqui O, Paul R, Gilfillan S, et al. Anti-human TREM2 induces microglia proliferation and reduces pathology in an Alzheimer’s disease model. J Exp Med. 2020;217:e20200785. 10.1084/jem.20200785 PMC7478730 32579671

[77] Long H, Simmons A, Mayorga A, Burgess B, Nguyen T, Budda B, Rychkova A, Rhinn H, Tassi I, Ward M, et al. Preclinical and first-in-human evaluation of AL002, a novel TREM2 agonistic antibody for Alzheimer’s disease. Alzheimers Res Ther. 2024;16:235. 39444037 10.1186/s13195-024-01599-1 PMC11515656

[78] Suarez-Calvet M, Kleinberger G, Araque Caballero MA, Brendel M, Rominger A, Alcolea D, Fortea J, Lleo A, Blesa R, Gispert JD, et al. sTREM2 cerebrospinal fluid levels are a potential biomarker for microglia activity in early-stage Alzheimer’s disease and associate with neuronal injury markers. EMBO Mol Med. 2016;8:466–76. 26941262 10.15252/emmm.201506123 PMC5120370

[79] Suarez-Calvet M, Araque Caballero MA, Kleinberger G, Bateman RJ, Fagan AM, Morris JC, Levin J, Danek A, Ewers M, Haass C, Dominantly Inherited Alzheimer N. Early changes in CSF sTREM2 in dominantly inherited Alzheimer’s disease occur after amyloid deposition and neuronal injury. Sci Transl Med. 2016;8:369ra178. 27974666 10.1126/scitranslmed.aag1767 PMC5385711

[80] Piccio L, Deming Y, Del-Aguila JL, Ghezzi L, Holtzman DM, Fagan AM, Fenoglio C, Galimberti D, Borroni B, Cruchaga C. Cerebrospinal fluid soluble TREM2 is higher in Alzheimer disease and associated with mutation status. Acta Neuropathol. 2016;131:925–33. 26754641 10.1007/s00401-016-1533-5 PMC4867123

[81] Heslegrave A, Heywood W, Paterson R, Magdalinou N, Svensson J, Johansson P, Ohrfelt A, Blennow K, Hardy J, Schott J, et al. Increased cerebrospinal fluid soluble TREM2 concentration in Alzheimer’s disease. Mol Neurodegener. 2016;11:3. 26754172 10.1186/s13024-016-0071-x PMC4709982

[82] Cummings J, Zhou Y, Lee G, Zhong K, Fonseca J, Cheng F. Not Available. Alzheimers Dement (N Y). 2024;10: e12465. 38659717 10.1002/trc2.12465 PMC11040692

[83] Vigil neuroscience announces interim data from its ongoing phase 1 clinical trial evaluating VG-3927 in healthy volunteers supporting continued development in Alzheimer’s disease. WATERTOWN, Mass., Jan. 23, 2025.

[84] Schlepckow K, Monroe KM, Kleinberger G, Cantuti-Castelvetri L, Parhizkar S, Xia D, Willem M, Werner G, Pettkus N, Brunner B, et al. Enhancing protective microglial activities with a dual function TREM2 antibody to the stalk region. EMBO Mol Med. 2020;12: e11227. 32154671 10.15252/emmm.201911227 PMC7136959

[85] van Lengerich B, Zhan L, Xia D, Chan D, Joy D, Park JI, Tatarakis D, Calvert M, Hummel S, Lianoglou S, et al. A TREM2-activating antibody with a blood-brain barrier transport vehicle enhances microglial metabolism in Alzheimer’s disease models. Nat Neurosci. 2023;26:416–29. 36635496 10.1038/s41593-022-01240-0 PMC9991924

[86] Estus S, Shaw BC, Devanney N, Katsumata Y, Press EE, Fardo DW. Evaluation of CD33 as a genetic risk factor for Alzheimer’s disease. Acta Neuropathol. 2019;138:187–99. 30949760 10.1007/s00401-019-02000-4 PMC7035471

[87] Griciuc A, Serrano-Pozo A, Parrado AR, Lesinski AN, Asselin CN, Mullin K, Hooli B, Choi SH, Hyman BT, Tanzi RE. Alzheimer’s disease risk gene CD33 inhibits microglial uptake of amyloid beta. Neuron. 2013;78:631–43. 23623698 10.1016/j.neuron.2013.04.014 PMC3706457

[88] Griciuc A, Federico AN, Natasan J, Forte AM, McGinty D, Nguyen H, Volak A, LeRoy S, Gandhi S, Lerner EP, et al. Gene therapy for Alzheimer’s disease targeting CD33 reduces amyloid beta accumulation and neuroinflammation. Hum Mol Genet. 2020;29:2920–35. 32803224 10.1093/hmg/ddaa179 PMC7566501

[89] Cummings J, Lee G, Zhong K, Fonseca J, Taghva K. Alzheimer’s disease drug development pipeline: 2021. Alzheimers Dement (N Y). 2021;7: e12179. 34095440 10.1002/trc2.12179 PMC8145448

[90] Rhinn H, Tatton N, McCaughey S, Kurnellas M, Rosenthal A. Progranulin as a therapeutic target in neurodegenerative diseases. Trends Pharmacol Sci. 2022;43:641–52. 35039149 10.1016/j.tips.2021.11.015

[91] Sung W, Noh MY, Nahm M, Kim YS, Ki CS, Kim YE, Kim HJ, Kim SH. Progranulin haploinsufficiency mediates cytoplasmic TDP-43 aggregation with lysosomal abnormalities in human microglia. J Neuroinflammation. 2024;21:47. 38347588 10.1186/s12974-024-03039-1 PMC10863104

[92] Lui H, Zhang J, Makinson SR, Cahill MK, Kelley KW, Huang HY, Shang Y, Oldham MC, Martens LH, Gao F, et al. Progranulin Deficiency Promotes Circuit-Specific Synaptic Pruning by Microglia via Complement Activation. Cell. 2016;165:921–35. 27114033 10.1016/j.cell.2016.04.001 PMC4860138

[93] Wu Y, Shao W, Todd TW, Tong J, Yue M, Koga S, Castanedes-Casey M, Librero AL, Lee CW, Mackenzie IR, et al. Microglial lysosome dysfunction contributes to white matter pathology and TDP-43 proteinopathy in GRN-associated FTD. Cell Rep. 2021;36: 109581. 34433069 10.1016/j.celrep.2021.109581 PMC8491969

[94] Sokolowski JD, Mandell JW. Phagocytic clearance in neurodegeneration. Am J Pathol. 2011;178:1416–28. 21435432 10.1016/j.ajpath.2010.12.051 PMC3078427

[95] Botelho RJ, Grinstein S. Phagocytosis. Curr Biol. 2011;21:R533-538. 21783028 10.1016/j.cub.2011.05.053

[96] Pluvinage JV, Haney MS, Smith BAH, Sun J, Iram T, Bonanno L, Li L, Lee DP, Morgens DW, Yang AC, et al. CD22 blockade restores homeostatic microglial phagocytosis in ageing brains. Nature. 2019;568:187–92. 30944478 10.1038/s41586-019-1088-4 PMC6574119

[97] Buckley CD, Gilroy DW, Serhan CN. Proresolving lipid mediators and mechanisms in the resolution of acute inflammation. Immunity. 2014;40:315–27. 24656045 10.1016/j.immuni.2014.02.009 PMC4004957

[98] Serhan CN, Hong S, Gronert K, Colgan SP, Devchand PR, Mirick G, Moussignac RL. Resolvins: a family of bioactive products of omega-3 fatty acid transformation circuits initiated by aspirin treatment that counter proinflammation signals. J Exp Med. 2002;196:1025–37. 12391014 10.1084/jem.20020760 PMC2194036

[99] Zhu M, Wang X, Hjorth E, Colas RA, Schroeder L, Granholm AC, Serhan CN, Schultzberg M. Pro-Resolving Lipid Mediators Improve Neuronal Survival and Increase Abeta42 Phagocytosis. Mol Neurobiol. 2016;53:2733–49. 26650044 10.1007/s12035-015-9544-0 PMC4824659

[100] Zhu M, Wang X, Sun L, Schultzberg M, Hjorth E. Can inflammation be resolved in Alzheimer’s disease? Ther Adv Neurol Disord. 2018;11:1756286418791107. 30116300 10.1177/1756286418791107 PMC6088473

[101] Wang X, Zhu M, Hjorth E, Cortes-Toro V, Eyjolfsdottir H, Graff C, Nennesmo I, Palmblad J, Eriksdotter M, Sambamurti K, et al. Resolution of inflammation is altered in Alzheimer’s disease. Alzheimers Dement. 2015;11(40–50):e41-42. 10.1016/j.jalz.2013.12.024 PMC4275415 24530025

[102] Lee JY, Han SH, Park MH, Baek B, Song IS, Choi MK, Takuwa Y, Ryu H, Kim SH, He X, et al. Neuronal SphK1 acetylates COX2 and contributes to pathogenesis in a model of Alzheimer’s Disease. Nat Commun. 2018;9:1479. 29662056 10.1038/s41467-018-03674-2 PMC5902554

[103] Lee JY, Han SH, Park MH, Song IS, Choi MK, Yu E, Park CM, Kim HJ, Kim SH, Schuchman EH, et al. N-AS-triggered SPMs are direct regulators of microglia in a model of Alzheimer’s disease. Nat Commun. 2020;11:2358. 32398649 10.1038/s41467-020-16080-4 PMC7217877

[104] Noh MY, Kwon MS, Oh KW, Nahm M, Park J, Kim YE, Ki CS, Jin HK, Bae JS, Kim SH. Role of NCKAP1 in the Defective Phagocytic Function of Microglia-Like Cells Derived from Rapidly Progressing Sporadic ALS. Mol Neurobiol. 2023;60:4761–77. 37154887 10.1007/s12035-023-03339-2 PMC10293423

[105] Noh MY, Kwon MS, Oh KW, Nahm M, Park J, Jin HK, Bae JS, Son B, Kim SH: miRNA-214 to predict progression and survival in ALS. J Neurol Neurosurg Psychiatry 2025 Jun 12;96(7):716-720. 10.1136/jnnp-2024-335177 PMC12322380 39915090

[106] Dong H, Yan J, Huang P, Wang X, Zhang R, Zhang C, Wang W, Qian W, Zhou J, Zhao Y, et al. miR-214-3p promotes the pathogenesis of Parkinson’s disease by inhibiting autophagy. Biomed Pharmacother. 2024;171: 116123. 38211424 10.1016/j.biopha.2024.116123

[107] Davis H, Attwell D. Plaque attack: Microglia have hard feelings toward amyloid-beta. Neuron. 2023;111:1–2. 36603547 10.1016/j.neuron.2022.11.014

[108] Zong B, Yu F, Zhang X, Pang Y, Zhao W, Sun P, Li L. Mechanosensitive Piezo1 channel in physiology and pathophysiology of the central nervous system. Ageing Res Rev. 2023;90: 102026. 37532007 10.1016/j.arr.2023.102026

[109] Hu J, Chen Q, Zhu H, Hou L, Liu W, Yang Q, Shen H, Chai G, Zhang B, Chen S, et al. Microglial Piezo1 senses Abeta fibril stiffness to restrict Alzheimer’s disease. Neuron. 2023;111(15–29): e18. 10.1016/j.neuron.2022.10.021 36368316

[110] Schweig JE, Yao H, Beaulieu-Abdelahad D, Ait-Ghezala G, Mouzon B, Crawford F, Mullan M, Paris D. Alzheimer’s disease pathological lesions activate the spleen tyrosine kinase. Acta Neuropathol Commun. 2017;5:69. 28877763 10.1186/s40478-017-0472-2 PMC5588676

[111] Yamaguchi T, Hamano T, Sada K, Asano R, Kanaan NM, Sasaki H, Yen SH, Kitazaki Y, Endo Y, Enomoto S, et al. Syk inhibitors reduce tau protein phosphorylation and oligomerization. Neurobiol Dis. 2024;201: 106656. 39233131 10.1016/j.nbd.2024.106656

[112] Wang S, Sudan R, Peng V, Zhou Y, Du S, Yuede CM, Lei T, Hou J, Cai Z, Cella M, et al. TREM2 drives microglia response to amyloid-beta via SYK-dependent and -independent pathways. Cell. 2022;185(4153–4169): e4119. 10.1016/j.cell.2022.09.033 PMC9625082 36306735

[113] Ennerfelt H, Frost EL, Shapiro DA, Holliday C, Zengeler KE, Voithofer G, Bolte AC, Lammert CR, Kulas JA, Ulland TK, Lukens JR. SYK coordinates neuroprotective microglial responses in neurodegenerative disease. Cell. 2022;185(4135–4152): e4122. 10.1016/j.cell.2022.09.030 PMC9617784 36257314

[114] Huang Y, Happonen KE, Burrola PG, O’Connor C, Hah N, Huang L, Nimmerjahn A, Lemke G. Microglia use TAM receptors to detect and engulf amyloid beta plaques. Nat Immunol. 2021;22:586–94. 33859405 10.1038/s41590-021-00913-5 PMC8102389

[115] Lew ED, Oh J, Burrola PG, Lax I, Zagorska A, Traves PG, Schlessinger J, Lemke G. Differential TAM receptor-ligand-phospholipid interactions delimit differential TAM bioactivities. Elife. 2014;3:e03385 10.7554/eLife.03385 PMC4206827 25265470

[116] Jung H, Lee SY, Lim S, Choi HR, Choi Y, Kim M, Kim S, Lee Y, Han KH, Chung WS, Kim CH. Anti-inflammatory clearance of amyloid-beta by a chimeric Gas6 fusion protein. Nat Med. 2022;28:1802–12. 35927581 10.1038/s41591-022-01926-9

[117] Kurochkin IV, Guarnera E, Berezovsky IN. Insulin-Degrading Enzyme in the Fight against Alzheimer’s Disease. Trends Pharmacol Sci. 2018;39:49–58. 29132916 10.1016/j.tips.2017.10.008

[118] Corraliza-Gomez M, Bermejo T, Lilue J, Rodriguez-Iglesias N, Valero J, Cozar-Castellano I, Arranz E, Sanchez D, Ganfornina MD. Insulin-degrading enzyme (IDE) as a modulator of microglial phenotypes in the context of Alzheimer’s disease and brain aging. J Neuroinflammation. 2023;20:233. 37817156 10.1186/s12974-023-02914-7 PMC10566021

[119] Qiu WQ, Walsh DM, Ye Z, Vekrellis K, Zhang J, Podlisny MB, Rosner MR, Safavi A, Hersh LB, Selkoe DJ. Insulin-degrading enzyme regulates extracellular levels of amyloid beta-protein by degradation. J Biol Chem. 1998;273:32730–8. 9830016 10.1074/jbc.273.49.32730

[120] Qiu WQ, Folstein MF. Insulin, insulin-degrading enzyme and amyloid-beta peptide in Alzheimer’s disease: review and hypothesis. Neurobiol Aging. 2006;27:190–8. 16399206 10.1016/j.neurobiolaging.2005.01.004

[121] Dundee JM, Puigdellivol M, Butler R, Brown GC. P2Y(6) Receptor-Dependent Microglial Phagocytosis of Synapses during Development Regulates Synapse Density and Memory. J Neurosci. 2023;43:8090–103. 37758475 10.1523/JNEUROSCI.1089-23.2023 PMC10697425

[122] Hou J, Chen Y, Cai Z, Heo GS, Yuede CM, Wang Z, Lin K, Saadi F, Trsan T, Nguyen AT, et al. Antibody-mediated targeting of human microglial leukocyte Ig-like receptor B4 attenuates amyloid pathology in a mouse model. Sci Transl Med. 2024;16:eadj9052. 38569016 10.1126/scitranslmed.adj9052 PMC11977387

[123] Yamanaka M, Ishikawa T, Griep A, Axt D, Kummer MP, Heneka MT. PPARgamma/RXRalpha-induced and CD36-mediated microglial amyloid-beta phagocytosis results in cognitive improvement in amyloid precursor protein/presenilin 1 mice. J Neurosci. 2012;32:17321–31. 23197723 10.1523/JNEUROSCI.1569-12.2012 PMC6621845

[124] Fu AK, Hung KW, Yuen MY, Zhou X, Mak DS, Chan IC, Cheung TH, Zhang B, Fu WY, Liew FY, Ip NY. IL-33 ameliorates Alzheimer’s disease-like pathology and cognitive decline. Proc Natl Acad Sci U S A. 2016;113:E2705-2713. 27091974 10.1073/pnas.1604032113 PMC4868478

[125] Boillee S, Yamanaka K, Lobsiger CS, Copeland NG, Jenkins NA, Kassiotis G, Kollias G, Cleveland DW. Onset and progression in inherited ALS determined by motor neurons and microglia. Science. 2006;312:1389–92. 16741123 10.1126/science.1123511

[126] Ilieva H, Polymenidou M, Cleveland DW. Non-cell autonomous toxicity in neurodegenerative disorders: ALS and beyond. J Cell Biol. 2009;187:761–72. 19951898 10.1083/jcb.200908164 PMC2806318

[127] Liao B, Zhao W, Beers DR, Henkel JS, Appel SH. Transformation from a neuroprotective to a neurotoxic microglial phenotype in a mouse model of ALS. Exp Neurol. 2012;237:147–52. 22735487 10.1016/j.expneurol.2012.06.011 PMC4126417

[128] Beers DR, Henkel JS, Zhao W, Wang J, Huang A, Wen S, Liao B, Appel SH. Endogenous regulatory T lymphocytes ameliorate amyotrophic lateral sclerosis in mice and correlate with disease progression in patients with amyotrophic lateral sclerosis. Brain. 2011;134:1293–314. 21596768 10.1093/brain/awr074 PMC3097891

[129] Lo Sicco C, Reverberi D, Balbi C, Ulivi V, Principi E, Pascucci L, Becherini P, Bosco MC, Varesio L, Franzin C, et al. Mesenchymal Stem Cell-Derived Extracellular Vesicles as Mediators of Anti-Inflammatory Effects: Endorsement of Macrophage Polarization. Stem Cells Transl Med. 2017;6:1018–28. 28186708 10.1002/sctm.16-0363 PMC5442783

[130] Song N, Scholtemeijer M, Shah K. Mesenchymal Stem Cell Immunomodulation: Mechanisms and Therapeutic Potential. Trends Pharmacol Sci. 2020;41:653–64. 32709406 10.1016/j.tips.2020.06.009 PMC7751844

[131] Liu YY, Li Y, Wang L, Zhao Y, Yuan R, Yang MM, Chen Y, Zhang H, Zhou FH, Qian ZR, Kang HJ. Mesenchymal stem cell-derived exosomes regulate microglia phenotypes: a promising treatment for acute central nervous system injury. Neural Regen Res. 2023;18:1657–65. 36751776 10.4103/1673-5374.363819 PMC10154505

[132] Noh MY, Lim SM, Oh KW, Cho KA, Park J, Kim KS, Lee SJ, Kwon MS, Kim SH. Mesenchymal Stem Cells Modulate the Functional Properties of Microglia via TGF-beta Secretion. Stem Cells Transl Med. 2016;5:1538–49. 27400795 10.5966/sctm.2015-0217 PMC5070497

[133] Oh KW, Noh MY, Kwon MS, Kim HY, Oh SI, Park J, Kim HJ, Ki CS, Kim SH. Repeated Intrathecal Mesenchymal Stem Cells for Amyotrophic Lateral Sclerosis. Ann Neurol. 2018;84:361–73. 30048006 10.1002/ana.25302 PMC6175096

[134] Kim SH, Oh KW, Noh MY, Kwon MS. Optimal Therapeutic Strategy of Bone Marrow-Originated Autologous Mesenchymal Stromal/Stem Cells for ALS. Stem Cells Transl Med. 2024;13:309–16. 38244235 10.1093/stcltm/szad095 PMC11016834

[135] Swarbrick S, Wragg N, Ghosh S, Stolzing A. Systematic Review of miRNA as Biomarkers in Alzheimer’s Disease. Mol Neurobiol. 2019;56:6156–67. 30734227 10.1007/s12035-019-1500-y PMC6682547

[136] Jadhav SP. MicroRNAs in microglia: deciphering their role in neurodegenerative diseases. Front Cell Neurosci. 2024;18:1391537. 38812793 10.3389/fncel.2024.1391537 PMC11133688

[137] Wan W, Liu G, Li X, Liu Y, Wang Y, Pan H, Hu J. MiR-191-5p alleviates microglial cell injury by targeting Map3k12 (mitogen-activated protein kinase kinase kinase 12) to inhibit the MAPK (mitogen-activated protein kinase) signaling pathway in Alzheimer’s disease. Bioengineered. 2021;12:12678–90. 34818971 10.1080/21655979.2021.2008638 PMC8810200

[138] Yin Z, Herron S, Silveira S, Kleemann K, Gauthier C, Mallah D, Cheng Y, Margeta MA, Pitts KM, Barry JL, et al. Identification of a protective microglial state mediated by miR-155 and interferon-gamma signaling in a mouse model of Alzheimer’s disease. Nat Neurosci. 2023;26:1196–207. 37291336 10.1038/s41593-023-01355-y PMC10619638

[139] Liang C, Zou T, Zhang M, Fan W, Zhang T, Jiang Y, Cai Y, Chen F, Chen X, Sun Y, et al. MicroRNA-146a switches microglial phenotypes to resist the pathological processes and cognitive degradation of Alzheimer’s disease. Theranostics. 2021;11:4103–21. 33754051 10.7150/thno.53418 PMC7977456

[140] Parisi C, Napoli G, Amadio S, Spalloni A, Apolloni S, Longone P, Volonte C. MicroRNA-125b regulates microglia activation and motor neuron death in ALS. Cell Death Differ. 2016;23:531–41. 26794445 10.1038/cdd.2015.153 PMC5072447

[141] Zingale VD, Gugliandolo A, Mazzon E. MiR-155: An Important Regulator of Neuroinflammation. Int J Mol Sci. 2021;23:90. 10.3390/ijms23010090 PMC8745074 35008513

[142] Butovsky O, Jedrychowski MP, Cialic R, Krasemann S, Murugaiyan G, Fanek Z, Greco DJ, Wu PM, Doykan CE, Kiner O, et al. Targeting miR-155 restores abnormal microglia and attenuates disease in SOD1 mice. Ann Neurol. 2015;77:75–99. 25381879 10.1002/ana.24304 PMC4432483

[143] Yao L, Zhu Z, Wu J, Zhang Y, Zhang H, Sun X, Qian C, Wang B, Xie L, Zhang S, Lu G. MicroRNA-124 regulates the expression of p62/p38 and promotes autophagy in the inflammatory pathogenesis of Parkinson’s disease. FASEB J. 2019;33:8648–65. 30995872 10.1096/fj.201900363R

[144] Gong X, Huang M, Chen L: Mechanism of miR-132–3p Promoting Neuroinflammation and Dopaminergic Neurodegeneration in Parkinson’s Disease. eNeuro 2022;9. 10.1523/ENEURO.0393-21.2021 PMC8805200 34983831

[145] Moutinho M, Puntambekar SS, Tsai AP, Coronel I, Lin PB, Casali BT, Martinez P, Oblak AL, Lasagna-Reeves CA, Lamb BT, Landreth GE. The niacin receptor HCAR2 modulates microglial response and limits disease progression in a mouse model of Alzheimer’s disease. Sci Transl Med. 2022;14:eabl7634. 35320002 10.1126/scitranslmed.abl7634 PMC10161396

[146] Kunkle BW, Grenier-Boley B, Sims R, Bis JC, Damotte V, Naj AC, Boland A, Vronskaya M, van der Lee SJ, Amlie-Wolf A, et al. Genetic meta-analysis of diagnosed Alzheimer’s disease identifies new risk loci and implicates Abeta, tau, immunity and lipid processing. Nat Genet. 2019;51:414–30. 30820047 10.1038/s41588-019-0358-2 PMC6463297

[147] Corder EH, Saunders AM, Strittmatter WJ, Schmechel DE, Gaskell PC, Small GW, Roses AD, Haines JL, Pericak-Vance MA. Gene dose of apolipoprotein E type 4 allele and the risk of Alzheimer’s disease in late onset families. Science. 1993;261:921–3. 8346443 10.1126/science.8346443

[148] Raulin AC, Doss SV, Trottier ZA, Ikezu TC, Bu G, Liu CC. ApoE in Alzheimer’s disease: pathophysiology and therapeutic strategies. Mol Neurodegener. 2022;17:72. 36348357 10.1186/s13024-022-00574-4 PMC9644639

[149] Mathys H, Davila-Velderrain J, Peng Z, Gao F, Mohammadi S, Young JZ, Menon M, He L, Abdurrob F, Jiang X, et al. Single-cell transcriptomic analysis of Alzheimer’s disease. Nature. 2019;570:332–7. 31042697 10.1038/s41586-019-1195-2 PMC6865822

[150] Cole GM, Ard MD. Influence of lipoproteins on microglial degradation of Alzheimer’s amyloid beta-protein. Microsc Res Tech. 2000;50:316–24. 10936886 10.1002/1097-0029(20000815)50:4<316::AID-JEMT11>3.0.CO;2-E

[151] Jiang Q, Lee CY, Mandrekar S, Wilkinson B, Cramer P, Zelcer N, Mann K, Lamb B, Willson TM, Collins JL, et al. ApoE promotes the proteolytic degradation of Abeta. Neuron. 2008;58:681–93. 18549781 10.1016/j.neuron.2008.04.010 PMC2493297

[152] Yin Z, Rosenzweig N, Kleemann KL, Zhang X, Brandao W, Margeta MA, Schroeder C, Sivanathan KN, Silveira S, Gauthier C, et al. APOE4 impairs the microglial response in Alzheimer’s disease by inducing TGFbeta-mediated checkpoints. Nat Immunol. 2023;24:1839–53. 37749326 10.1038/s41590-023-01627-6 PMC10863749

[153] Haney MS, Palovics R, Munson CN, Long C, Johansson PK, Yip O, Dong W, Rawat E, West E, Schlachetzki JCM, et al. APOE4/4 is linked to damaging lipid droplets in Alzheimer’s disease microglia. Nature. 2024;628:154–61. 38480892 10.1038/s41586-024-07185-7 PMC10990924

[154] Eskandari-Sedighi G, Blurton-Jones M. Microglial APOE4: more is less and less is more. Mol Neurodegener. 2023;18:99. 38115077 10.1186/s13024-023-00693-6 PMC10729371

[155] Victor MB, Leary N, Luna X, Meharena HS, Scannail AN, Bozzelli PL, Samaan G, Murdock MH, von Maydell D, Effenberger AH, et al. Lipid accumulation induced by APOE4 impairs microglial surveillance of neuronal-network activity. Cell Stem Cell. 2022;29(1197–1212): e1198. 10.1016/j.stem.2022.07.005 PMC9623845 35931030

[156] Yeh FL, Wang Y, Tom I, Gonzalez LC, Sheng M. TREM2 Binds to Apolipoproteins, Including APOE and CLU/APOJ, and Thereby Facilitates Uptake of Amyloid-Beta by Microglia. Neuron. 2016;91:328–40. 27477018 10.1016/j.neuron.2016.06.015

[157] Sayed FA, Kodama L, Fan L, Carling GK, Udeochu JC, Le D, Li Q, Zhou L, Wong MY, Horowitz R, et al. AD-linked R47H-TREM2 mutation induces disease-enhancing microglial states via AKT hyperactivation. Sci Transl Med. 2021;13:eabe3947. 34851693 10.1126/scitranslmed.abe3947 PMC9345574

[158] Piers TM, Cosker K, Mallach A, Johnson GT, Guerreiro R, Hardy J, Pocock JM. A locked immunometabolic switch underlies TREM2 R47H loss of function in human iPSC-derived microglia. FASEB J. 2020;34:2436–50. 31907987 10.1096/fj.201902447R PMC7027848

[159] Baik SH, Kang S, Lee W, Choi H, Chung S, Kim JI, Mook-Jung I. A Breakdown in Metabolic Reprogramming Causes Microglia Dysfunction in Alzheimer’s Disease. Cell Metab. 2019;30(493–507): e496. 10.1016/j.cmet.2019.06.005 31257151

[160] McGettrick AF, O’Neill LAJ. The Role of HIF in Immunity and Inflammation. Cell Metab. 2020;32:524–36. 32853548 10.1016/j.cmet.2020.08.002

[161] Iwasaki Y, Takeshima Y, Fujio K. Basic mechanism of immune system activation by mitochondria. Immunol Med. 2020;43:142–7. 32393116 10.1080/25785826.2020.1756609

[162] Xu Y, Chen Y, Zhang X, Ma J, Liu Y, Cui L, Wang F. Glycolysis in Innate Immune Cells Contributes to Autoimmunity. Front Immunol. 2022;13: 920029. 35844594 10.3389/fimmu.2022.920029 PMC9284233

[163] Chen H, Guo Z, Sun Y, Dai X. The immunometabolic reprogramming of microglia in Alzheimer’s disease. Neurochem Int. 2023;171: 105614. 37748710 10.1016/j.neuint.2023.105614

[164] Prakash P, Manchanda P, Paouri E, Bisht K, Sharma K, Rajpoot J, Wendt V, Hossain A, Wijewardhane PR, Randolph CE, et al. Amyloid beta Induces Lipid Droplet-Mediated Microglial Dysfunction in Alzheimer’s Disease. Immunity. 2025;58:1536-52. 10.1016/j.immuni.2025.04.029 PMC12168635 40393454

[165] Leng L, Yuan Z, Pan R, Su X, Wang H, Xue J, Zhuang K, Gao J, Chen Z, Lin H, et al. Microglial hexokinase 2 deficiency increases ATP generation through lipid metabolism leading to beta-amyloid clearance. Nat Metab. 2022;4:1287–305. 36203054 10.1038/s42255-022-00643-4

[166] Rawji KS, Young AMH, Ghosh T, Michaels NJ, Mirzaei R, Kappen J, Kolehmainen KL, Alaeiilkhchi N, Lozinski B, Mishra MK, et al. Niacin-mediated rejuvenation of macrophage/microglia enhances remyelination of the aging central nervous system. Acta Neuropathol. 2020;139:893–909. 32030468 10.1007/s00401-020-02129-7 PMC7181452

[167] Pan RY, He L, Zhang J, Liu X, Liao Y, Gao J, Liao Y, Yan Y, Li Q, Zhou X, et al. Positive feedback regulation of microglial glucose metabolism by histone H4 lysine 12 lactylation in Alzheimer’s disease. Cell Metab. 2022;34(634–648): e636. 10.1016/j.cmet.2022.02.013 35303422

[168] Almer G, Teismann P, Stevic Z, Halaschek-Wiener J, Deecke L, Kostic V, Przedborski S. Increased levels of the pro-inflammatory prostaglandin PGE2 in CSF from ALS patients. Neurology. 2002;58:1277–9. 11971099 10.1212/wnl.58.8.1277

[169] Ilzecka J. Prostaglandin E2 is increased in amyotrophic lateral sclerosis patients. Acta Neurol Scand. 2003;108:125–9. 12859290 10.1034/j.1600-0404.2003.00102.x

[170] Ho L, Luterman JD, Aisen PS, Pasinetti GM, Montine TJ, Morrow JD. Elevated CSF prostaglandin E2 levels in patients with probable AD. Neurology. 2000;55:323. 10908926 10.1212/wnl.55.2.323

[171] Minhas PS, Latif-Hernandez A, McReynolds MR, Durairaj AS, Wang Q, Rubin A, Joshi AU, He JQ, Gauba E, Liu L, et al. Restoring metabolism of myeloid cells reverses cognitive decline in ageing. Nature. 2021;590:122–8. 33473210 10.1038/s41586-020-03160-0 PMC8274816

[172] Hu Y, Hruscha A, Pan C, Schifferer M, Schmidt MK, Nuscher B, Giera M, Kostidis S, Burhan O, van Bebber F, et al. Mis-localization of endogenous TDP-43 leads to ALS-like early-stage metabolic dysfunction and progressive motor deficits. Mol Neurodegener. 2024;19:50. 38902734 10.1186/s13024-024-00735-7 PMC11188230

[173] Paolicelli RC, Jawaid A, Henstridge CM, Valeri A, Merlini M, Robinson JL, Lee EB, Rose J, Appel S, Lee VM, et al. TDP-43 Depletion in Microglia Promotes Amyloid Clearance but Also Induces Synapse Loss. Neuron. 2017;95(297–308): e296. 10.1016/j.neuron.2017.05.037 PMC5519492 28669544

[174] Hamad AA, Amer BE, Hawas Y, Mabrouk MA, Meshref M. Masitinib as a neuroprotective agent: a scoping review of preclinical and clinical evidence. Neurol Sci. 2024;45:1861–73. 38105307 10.1007/s10072-023-07259-w PMC11021265

[175] Li T, Martin E, Abada YS, Boucher C, Ces A, Youssef I, Fenaux G, Forand Y, Legrand A, Nachiket N, et al. Effects of Chronic Masitinib Treatment in APPswe/PSEN1dE9 Transgenic Mice Modeling Alzheimer’s Disease. J Alzheimers Dis. 2020;76:1339–45. 32623401 10.3233/JAD-200466

[176] Forlenza OV, Diniz BS, Talib LL, Mendonca VA, Ojopi EB, Gattaz WF, Teixeira AL. Increased serum IL-1beta level in Alzheimer’s disease and mild cognitive impairment. Dement Geriatr Cogn Disord. 2009;28:507–12. 19996595 10.1159/000255051

[177] Melchiorri D, Merlo S, Micallef B, Borg JJ, Drafi F. Alzheimer’s disease and neuroinflammation: will new drugs in clinical trials pave the way to a multi-target therapy? Front Pharmacol. 2023;14:1196413. 37332353 10.3389/fphar.2023.1196413 PMC10272781

[178] Yazar T, Olgun Yazar H, Cihan M. Evaluation of serum galectin-3 levels at Alzheimer patients by stages: a preliminary report. Acta Neurol Belg. 2021;121:949–54. 32852752 10.1007/s13760-020-01477-1

[179] Boza-Serrano A, Vrillon A, Minta K, Paulus A, Camprubi-Ferrer L, Garcia M, Andreasson U, Antonell A, Wennstrom M, Gouras G, et al. Galectin-3 is elevated in CSF and is associated with Abeta deposits and tau aggregates in brain tissue in Alzheimer’s disease. Acta Neuropathol. 2022;144:843–59. 35895141 10.1007/s00401-022-02469-6 PMC9547798

[180] Zheng H, Liu Q, Zhou S, Luo H, Zhang W. Role and therapeutic targets of P2X7 receptors in neurodegenerative diseases. Front Immunol. 2024;15:1345625. 38370420 10.3389/fimmu.2024.1345625 PMC10869479

[181] Lobo A: First patient dosed in Parkinson’s clinical trial of Ventus’ VENT-02. In *Book First patient dosed in Parkinson’s clinical trial of Ventus’ VENT-02* (Editor ed.^eds.). City; 2025.

[182] Swanson KV, Deng M, Ting JP. The NLRP3 inflammasome: molecular activation and regulation to therapeutics. Nat Rev Immunol. 2019;19:477–89. 31036962 10.1038/s41577-019-0165-0 PMC7807242

[183] Chen X, Zhang P, Zhang Y, Wei M, Tian T, Zhu D, Guan Y, Wei W, Ma Y. The research progression of direct NLRP3 inhibitors to treat inflammatory disorders. Cell Immunol. 2024;397–398: 104810. 38324950 10.1016/j.cellimm.2024.104810

[184] Lonnemann N, Hosseini S, Marchetti C, Skouras DB, Stefanoni D, D’Alessandro A, Dinarello CA, Korte M. The NLRP3 inflammasome inhibitor OLT1177 rescues cognitive impairment in a mouse model of Alzheimer’s disease. Proc Natl Acad Sci U S A. 2020;117:32145–54. 33257576 10.1073/pnas.2009680117 PMC7749353

[185] Flores J, Fillion ML, LeBlanc AC. Caspase-1 inhibition improves cognition without significantly altering amyloid and inflammation in aged Alzheimer disease mice. Cell Death Dis. 2022;13:864. 36220815 10.1038/s41419-022-05290-x PMC9553979

[186] Davide Basco P: Targeting the Inflammasome Pathway with an ANTI-ASC Immunotherapy in Alzheimer’s Disease. AD/PD 2024.

[187] Ofengeim D, Yuan J. Regulation of RIP1 kinase signalling at the crossroads of inflammation and cell death. Nat Rev Mol Cell Biol. 2013;14:727–36. 24129419 10.1038/nrm3683

[188] Ito Y, Ofengeim D, Najafov A, Das S, Saberi S, Li Y, Hitomi J, Zhu H, Chen H, Mayo L, et al. RIPK1 mediates axonal degeneration by promoting inflammation and necroptosis in ALS. Science. 2016;353:603–8. 27493188 10.1126/science.aaf6803 PMC5444917

[189] Ofengeim D, Mazzitelli S, Ito Y, DeWitt JP, Mifflin L, Zou C, Das S, Adiconis X, Chen H, Zhu H, et al. RIPK1 mediates a disease-associated microglial response in Alzheimer’s disease. Proc Natl Acad Sci U S A. 2017;114:E8788–97. 28904096 10.1073/pnas.1714175114 PMC5642727

[190] Iannielli A, Bido S, Folladori L, Segnali A, Cancellieri C, Maresca A, Massimino L, Rubio A, Morabito G, Caporali L, et al. Pharmacological Inhibition of Necroptosis Protects from Dopaminergic Neuronal Cell Death in Parkinson’s Disease Models. Cell Rep. 2018;22:2066–79. 29466734 10.1016/j.celrep.2018.01.089 PMC5842028

[191] Grievink HW, Heuberger J, Huang F, Chaudhary R, Birkhoff WAJ, Tonn GR, Mosesova S, Erickson R, Moerland M, Haddick PCG, et al. DNL104, a Centrally Penetrant RIPK1 Inhibitor, Inhibits RIP1 Kinase Phosphorylation in a Randomized Phase I Ascending Dose Study in Healthy Volunteers. Clin Pharmacol Ther. 2020;107:406–14. 31437302 10.1002/cpt.1615

[192] Weisel K, Scott N, Berger S, Wang S, Brown K, Powell M, Broer M, Watts C, Tompson DJ, Burriss SW, et al. A randomised, placebo-controlled study of RIPK1 inhibitor GSK2982772 in patients with active ulcerative colitis. BMJ Open Gastroenterol 2021;8:e000680 10.1136/bmjgast-2021-000680 PMC8365785 34389633

[193] Jones NS, Kshirsagar S, Mohanan V, Ramakrishnan V, Di Nucci F, Ma L, Mao J, Ding H, Klabunde S, Vucic D, et al. A phase I, randomized, ascending-dose study to assess safety, pharmacokinetics, and activity of GDC-8264, a RIP1 inhibitor, in healthy volunteers. Clin Transl Sci. 2023;16:1997–2009. 37596814 10.1111/cts.13607 PMC10582670

[194] Tolosa E, Vila M, Klein C, Rascol O. LRRK2 in Parkinson disease: challenges of clinical trials. Nat Rev Neurol. 2020;16:97–107. 31980808 10.1038/s41582-019-0301-2

[195] Moehle MS, Webber PJ, Tse T, Sukar N, Standaert DG, DeSilva TM, Cowell RM, West AB. LRRK2 inhibition attenuates microglial inflammatory responses. J Neurosci. 2012;32:1602–11. 22302802 10.1523/JNEUROSCI.5601-11.2012 PMC3532034

[196] Russo I, Berti G, Plotegher N, Bernardo G, Filograna R, Bubacco L, Greggio E. Leucine-rich repeat kinase 2 positively regulates inflammation and down-regulates NF-kappaB p50 signaling in cultured microglia cells. J Neuroinflammation. 2015;12:230. 26646749 10.1186/s12974-015-0449-7 PMC4673731

[197] Langston RG, Beilina A, Reed X, Kaganovich A, Singleton AB, Blauwendraat C, Gibbs JR, Cookson MR. Association of a common genetic variant with Parkinson’s disease is mediated by microglia. Sci Transl Med. 2022;14:eabp8869. 35895835 10.1126/scitranslmed.abp8869 PMC9809150

[198] Jennings D, Huntwork-Rodriguez S, Vissers M, Daryani VM, Diaz D, Goo MS, Chen JJ, Maciuca R, Fraser K, Mabrouk OS, et al. LRRK2 Inhibition by BIIB122 in Healthy Participants and Patients with Parkinson’s Disease. Mov Disord. 2023;38:386–98. 36807624 10.1002/mds.29297

[199] Naaldijk Y, Fernandez B, Fasiczka R, Fdez E, Leghay C, Croitoru I, Kwok JB, Boulesnane Y, Vizeneux A, Mutez E, et al. A potential patient stratification biomarker for Parkinson s disease based on LRRK2 kinase-mediated centrosomal alterations in peripheral blood-derived cells. NPJ Parkinsons Dis. 2024;10:12. 38191886 10.1038/s41531-023-00624-8 PMC10774440

[200] Lin Z, Chen C, Yang D, Ding J, Wang G, Ren H. DJ-1 inhibits microglial activation and protects dopaminergic neurons in vitro and in vivo through interacting with microglial p65. Cell Death Dis. 2021;12:715. 34274951 10.1038/s41419-021-04002-1 PMC8286256

[201] Kim JH, Choi DJ, Jeong HK, Kim J, Kim DW, Choi SY, Park SM, Suh YH, Jou I, Joe EH. DJ-1 facilitates the interaction between STAT1 and its phosphatase, SHP-1, in brain microglia and astrocytes: A novel anti-inflammatory function of DJ-1. Neurobiol Dis. 2013;60:1–10. 23969237 10.1016/j.nbd.2013.08.007

[202] Trudler D, Weinreb O, Mandel SA, Youdim MB, Frenkel D. DJ-1 deficiency triggers microglia sensitivity to dopamine toward a pro-inflammatory phenotype that is attenuated by rasagiline. J Neurochem. 2014;129:434–47. 24355073 10.1111/jnc.12633

[203] Group F-NBW: FDA-NIH Biomarker Working Group BEST (Biomarkers, EndpointS, and Other Tools) Resource. Food and Drug Administration (US), Silver Spring, MD (2016) Co-published by National Institutes of Health (US): Bethesda (MD) (https://www.ncbinlmnihgov/books/NBK326791/, consulted in February 2023) 2016.

[204] In *BEST (Biomarkers, EndpointS, and other Tools) Resource.* Silver Spring (MD) Bethesda (MD); 2016

[205] Sherwani SI, Khan HA, Ekhzaimy A, Masood A, Sakharkar MK. Significance of HbA1c Test in Diagnosis and Prognosis of Diabetic Patients. Biomark Insights. 2016;11:95–104. 27398023 10.4137/BMI.S38440 PMC4933534

[206] Cusumano AM, Tzanno-Martins C, Rosa-Diez GJ. The Glomerular Filtration Rate: From the Diagnosis of Kidney Function to a Public Health Tool. Front Med (Lausanne). 2021;8: 769335. 34926510 10.3389/fmed.2021.769335 PMC8675900

[207] Hsu S, Gordon BA, Hornbeck R, Norton JB, Levitch D, Louden A, Ziegemeier E, Laforce R Jr, Chhatwal J, Day GS, et al. Discovery and validation of autosomal dominant Alzheimer’s disease mutations. Alzheimer’s research & therapy. 2018;10:67. 10.1186/s13195-018-0392-9 PMC6052673 30021643

[208] Fung S, Smith CL, Prater KE, Case A, Green K, Osnis L, Winston C, Kinoshita Y, Sopher B, Morrison RS, et al. Early-Onset Familial Alzheimer Disease Variant PSEN2 N141I Heterozygosity is Associated with Altered Microglia Phenotype. J Alzheimers Dis. 2020;77:675–88. 32741831 10.3233/JAD-200492 PMC7592656

[209] Zhao A, Jiao Y, Ye G, Kang W, Tan L, Li Y, Deng Y, Liu J, Alzheimer’s Disease Neuroimaging I. Soluble TREM2 levels associate with conversion from mild cognitive impairment to Alzheimer’s disease. J Clin Invest 2022;132:e158708 10.1172/JCI158708 PMC9753995 36519540

[210] Morenas-Rodriguez E, Li Y, Nuscher B, Franzmeier N, Xiong C, Suarez-Calvet M, Fagan AM, Schultz S, Gordon BA, Benzinger TLS, et al. Soluble TREM2 in CSF and its association with other biomarkers and cognition in autosomal-dominant Alzheimer’s disease: a longitudinal observational study. Lancet Neurol. 2022;21:329–41. 35305339 10.1016/S1474-4422(22)00027-8 PMC8926925

[211] Bu XL, Sun PY, Fan DY, Wang J, Sun HL, Cheng Y, Zeng GH, Chen DW, Li HY, Yi X, et al. Associations of plasma soluble CD22 levels with brain amyloid burden and cognitive decline in Alzheimer’s disease. Sci Adv. 2022;8:eabm5667. 35363517 10.1126/sciadv.abm5667 PMC10938586

[212] Jack CR Jr, Andrews JS, Beach TG, Buracchio T, Dunn B, Graf A, Hansson O, Ho C, Jagust W, McDade E, et al. Revised criteria for diagnosis and staging of Alzheimer’s disease: Alzheimer’s Association Workgroup. Alzheimers Dement. 2024;20:5143–69. 38934362 10.1002/alz.13859 PMC11350039

[213] Huang Y, Wei J, Cooper A, Morris MJ. Parkinson’s disease: From genetics to molecular dysfunction and targeted therapeutic approaches. Genes Dis. 2023;10:786–98. 37396535 10.1016/j.gendis.2021.12.015 PMC10308076

[214] Lind-Holm Mogensen F, Scafidi A, Poli A, Michelucci A. PARK7/DJ-1 in microglia: implications in Parkinson’s disease and relevance as a therapeutic target. J Neuroinflammation. 2023;20:95. 37072827 10.1186/s12974-023-02776-z PMC10111685

[215] Dwyer Z, Rudyk C, Thompson A, Farmer K, Fenner B, Fortin T, Derksen A, Sun H, Hayley S. Clint: Leucine-rich repeat kinase-2 (LRRK2) modulates microglial phenotype and dopaminergic neurodegeneration. Neurobiol Aging. 2020;91:45–55. 32247534 10.1016/j.neurobiolaging.2020.02.017

[216] Calabresi P, Mechelli A, Natale G, Volpicelli-Daley L, Di Lazzaro G, Ghiglieri V. Alpha-synuclein in Parkinson’s disease and other synucleinopathies: from overt neurodegeneration back to early synaptic dysfunction. Cell Death Dis. 2023;14:176. 36859484 10.1038/s41419-023-05672-9 PMC9977911

[217] Yamashita KY, Bhoopatiraju S, Silverglate BD, Grossberg GT. Biomarkers in Parkinson’s disease: A state of the art review. Biomarkers in Neuropsychiatry. 2023;9: 100074.

[218] Maurel C, Dangoumau A, Marouillat S, Brulard C, Chami A, Hergesheimer R, Corcia P, Blasco H, Andres CR. Vourc’h P: Causative Genes in Amyotrophic Lateral Sclerosis and Protein Degradation Pathways: a Link to Neurodegeneration. Mol Neurobiol. 2018;55:6480–99. 29322304 10.1007/s12035-017-0856-0

[219] Greaves CV, Rohrer JD. An update on genetic frontotemporal dementia. J Neurol. 2019;266:2075–86. 31119452 10.1007/s00415-019-09363-4 PMC6647117

[220] Reijn TS, Abdo WF, Schelhaas HJ, Verbeek MM. CSF neurofilament protein analysis in the differential diagnosis of ALS. J Neurol. 2009;256:615–9. 19296046 10.1007/s00415-009-0131-z

[221] Albagli EA, Calliari A, Gendron TF, Zhang YJ. HDGFL2 cryptic protein: a portal to detection and diagnosis in neurodegenerative disease. Mol Neurodegener. 2024;19:79. 39456026 10.1186/s13024-024-00768-y PMC11515212

[222] Suarez-Calvet M, Capell A, Araque Caballero MA, Morenas-Rodriguez E, Fellerer K, Franzmeier N, Kleinberger G, Eren E, Deming Y, Piccio L, et al. CSF progranulin increases in the course of Alzheimer’s disease and is associated with sTREM2, neurodegeneration and cognitive decline. EMBO Mol Med. 2018;10:e9712. 10.15252/emmm.201809712 PMC6284390 30482868

[223] Batzu L, Westman E, Pereira JB. Alzheimer’s Disease Neuroimaging I: Cerebrospinal fluid progranulin is associated with increased cortical thickness in early stages of Alzheimer’s disease. Neurobiol Aging. 2020;88:61–70. 31980280 10.1016/j.neurobiolaging.2019.12.012

[224] Kulczynska-Przybik A, Slowik A, Mroczko P, Borawski B, Groblewska M, Borawska R, Mroczko B. Cerebrospinal Fluid and Blood CX3CL1 as a Potential Biomarker in Early Diagnosis and Prognosis of Dementia. Curr Alzheimer Res. 2020;17:709–21. 33167838 10.2174/1567205017666201109095657

[225] Arranz AM, De Strooper B. The role of astroglia in Alzheimer’s disease: pathophysiology and clinical implications. Lancet Neurol. 2019;18:406–14. 30795987 10.1016/S1474-4422(18)30490-3

[226] Mavroudis I, Chowdhury R, Petridis F, Karantali E, Chatzikonstantinou S, Balmus IM, Luca IS, Ciobica A, Kazis D. YKL-40 as a potential biomarker for the differential diagnosis of Alzheimer’s disease. Medicina (Kaunas, Lithuania). 2021;58:60. 10.3390/medicina58010060 PMC8777884 35056368

[227] Muszyński P, Groblewska M, Kulczyńska-Przybik A, Kułakowska A, Mroczko B. YKL-40 as a potential biomarker and a possible target in therapeutic strategies of Alzheimer’s disease. Curr Neuropharmacol. 2017;15:906–17. 28183245 10.2174/1570159X15666170208124324 PMC5652033

[228] Sutphen CL, McCue L, Herries EM, Xiong C, Ladenson JH, Holtzman DM, Fagan AM. Adni: Longitudinal decreases in multiple cerebrospinal fluid biomarkers of neuronal injury in symptomatic late onset Alzheimer’s disease. Alzheimers Dement. 2018;14:869–79. 29580670 10.1016/j.jalz.2018.01.012 PMC6110083

[229] Franzmeier N, Dehsarvi A, Steward A, Biel D, Dewenter A, Roemer SN, Wagner F, Gross M, Brendel M, Moscoso A, et al. Elevated CSF GAP-43 is associated with accelerated tau accumulation and spread in Alzheimer’s disease. Nat Commun. 2024;15:202. 38172114 10.1038/s41467-023-44374-w PMC10764818

[230] Portelius E, Olsson B, Hoglund K, Cullen NC, Kvartsberg H, Andreasson U, Zetterberg H, Sandelius A, Shaw LM, Lee VMY, et al. Cerebrospinal fluid neurogranin concentration in neurodegeneration: relation to clinical phenotypes and neuropathology. Acta Neuropathol. 2018;136:363–76. 29700597 10.1007/s00401-018-1851-x PMC6096740

[231] Clarke MTM, Brinkmalm A, Foiani MS, Woollacott IOC, Heller C, Heslegrave A, Keshavan A, Fox NC, Schott JM, Warren JD, et al. CSF synaptic protein concentrations are raised in those with atypical Alzheimer’s disease but not frontotemporal dementia. Alzheimers Res Ther. 2019;11:105. 31847891 10.1186/s13195-019-0564-2 PMC6918699

[232] Liu W, Lin H, He X, Chen L, Dai Y, Jia W, Xue X, Tao J, Chen L. Neurogranin as a cognitive biomarker in cerebrospinal fluid and blood exosomes for Alzheimer’s disease and mild cognitive impairment. Transl Psychiatry. 2020;10:125. 32350238 10.1038/s41398-020-0801-2 PMC7190828

[233] Heller C, Foiani MS, Moore K, Convery R, Bocchetta M, Neason M, Cash DM, Thomas D, Greaves CV, Woollacott IO, et al. Plasma glial fibrillary acidic protein is raised in progranulin-associated frontotemporal dementia. J Neurol Neurosurg Psychiatry. 2020;91:263–70. 31937580 10.1136/jnnp-2019-321954

[234] Verberk IMW, Thijssen E, Koelewijn J, Mauroo K, Vanbrabant J, de Wilde A, Zwan MD, Verfaillie SCJ, Ossenkoppele R, Barkhof F, et al. Combination of plasma amyloid beta((1–42/1-40)) and glial fibrillary acidic protein strongly associates with cerebral amyloid pathology. Alzheimers Res Ther. 2020;12:118. 32988409 10.1186/s13195-020-00682-7 PMC7523295

[235] Bellaver B, Povala G, Ferreira PCL, Ferrari-Souza JP, Leffa DT, Lussier FZ, Benedet AL, Ashton NJ, Triana-Baltzer G, Kolb HC, et al. Astrocyte reactivity influences amyloid-β effects on tau pathology in preclinical Alzheimer’s disease. Nat Med. 2023;29:1775–81. 37248300 10.1038/s41591-023-02380-x PMC10353939

[236] Gundogdu F, Soylu F, Erkan L, Tatli O, Mavi S, Yavuzcan A. The role of serum CA-125 levels and CA-125 tissue expression positivity in the prediction of the recurrence of stage III and IV epithelial ovarian tumors (CA-125 levels and tissue CA-125 in ovarian tumors). Arch Gynecol Obstet. 2011;283:1397–402. 20645105 10.1007/s00404-010-1589-8

[237] Rustin GJ, Marples M, Nelstrop AE, Mahmoudi M, Meyer T. Use of CA-125 to define progression of ovarian cancer in patients with persistently elevated levels. J Clin Oncol. 2001;19:4054–7. 11600607 10.1200/JCO.2001.19.20.4054

[238] Nakanishi H. Microglial cathepsin B as a key driver of inflammatory brain diseases and brain aging. Neural Regen Res. 2020;15:25–9. 31535638 10.4103/1673-5374.264444 PMC6862407

[239] Bai H, Yang B, Yu W, Xiao Y, Yu D, Zhang Q. Cathepsin B links oxidative stress to the activation of NLRP3 inflammasome. Exp Cell Res. 2018;362:180–7. 29196167 10.1016/j.yexcr.2017.11.015

[240] Do KV, Hjorth E, Wang Y, Jun B, Kautzmann MI, Ohshima M, Eriksdotter M, Schultzberg M, Bazan NG. Cerebrospinal Fluid Profile of Lipid Mediators in Alzheimer’s Disease. Cell Mol Neurobiol. 2023;43:797–811. 35362880 10.1007/s10571-022-01216-5 PMC9957874

[241] Jiang Y, Uhm H, Ip FC, Ouyang L, Lo RMN, Cheng EYL, Cao X, Tan CMC, Law BCH, Ortiz-Romero P, et al. A blood-based multi-pathway biomarker assay for early detection and staging of Alzheimer’s disease across ethnic groups. Alzheimers Dement. 2024;20:2000–15. 38183344 10.1002/alz.13676 PMC10984431

[242] Ashutosh K. W, Cotter R, Borgmann K, Wu L, Persidsky R, Sakhuja N, Ghorpade A: CXCL8 protects human neurons from amyloid-β-induced neurotoxicity: relevance to Alzheimer’s disease. Biochem Biophys Res Commun. 2011;412:565–71. 21840299 10.1016/j.bbrc.2011.07.127 PMC3236067

[243] Kiyota T, Gendelman HE, Weir RA, Higgins EE, Zhang G, Jain M. CCL2 affects β-amyloidosis and progressive neurocognitive dysfunction in a mouse model of Alzheimer’s disease. Neurobiol Aging. 2013;34:1060–8. 23040664 10.1016/j.neurobiolaging.2012.08.009 PMC4011558

[244] Mintun MA, Lo AC, Duggan Evans C, Wessels AM, Ardayfio PA, Andersen SW, Shcherbinin S, Sparks J, Sims JR, Brys M, et al. Donanemab in Early Alzheimer’s Disease. N Engl J Med. 2021;384:1691–704. 33720637 10.1056/NEJMoa2100708

[245] van Dyck CH, Swanson CJ, Aisen P, Bateman RJ, Chen C, Gee M, Kanekiyo M, Li D, Reyderman L, Cohen S, et al. Lecanemab in Early Alzheimer’s Disease. N Engl J Med. 2023;388:9–21. 36449413 10.1056/NEJMoa2212948

[246] Menon S, Armstrong S, Hamzeh A, Visanji NP, Sardi SP, Tandon A. Alpha-Synuclein targeting therapeutics for Parkinson’s disease and related synucleinopathies. Front Neurol. 2022;13: 852003. 35614915 10.3389/fneur.2022.852003 PMC9124903

[247] Steinacker P, Verde F, Fang L, Feneberg E, Oeckl P, Roeber S, Anderl-Straub S, Danek A, Diehl-Schmid J, Fassbender K, et al. Chitotriosidase (CHIT1) is increased in microglia and macrophages in spinal cord of amyotrophic lateral sclerosis and cerebrospinal fluid levels correlate with disease severity and progression. J Neurol Neurosurg Psychiatry. 2018;89:239–47. 29142138 10.1136/jnnp-2017-317138

[248] Gille B, De Schaepdryver M, Dedeene L, Goossens J, Claeys KG, Van Den Bosch L, Tournoy J, Van Damme P, Poesen K. Inflammatory markers in cerebrospinal fluid: independent prognostic biomarkers in amyotrophic lateral sclerosis? J Neurol Neurosurg Psychiatry. 2019;90:1338–46. 31175169 10.1136/jnnp-2018-319586

[249] Huang F, Zhu Y, Hsiao-Nakamoto J, Tang X, Dugas JC, Moscovitch-Lopatin M, Glass JD, Brown RH Jr, Ladha SS, Lacomis D, et al. Longitudinal biomarkers in amyotrophic lateral sclerosis. Ann Clin Transl Neurol. 2020;7:1103–16. 32515902 10.1002/acn3.51078 PMC7359115

[250] Ehrhart J, Smith AJ, Kuzmin-Nichols N, Zesiewicz TA, Jahan I, Shytle RD, Kim SH, Sanberg CD, Vu TH, Gooch CL, et al. Humoral factors in ALS patients during disease progression. J Neuroinflammation. 2015;12:127. 26126965 10.1186/s12974-015-0350-4 PMC4487852

[251] Mashima R, Sakai E, Tanaka M, Kosuga M, Okuyama T. The levels of urinary glycosaminoglycans of patients with attenuated and severe type of mucopolysaccharidosis II determined by liquid chromatography-tandem mass spectrometry. Mol Genet Metab Rep. 2016;7:87–91. 27331006 10.1016/j.ymgmr.2016.03.009 PMC4908047

[252] Salerno S, Viviano M, Baglini E, Poggetti V, Giorgini D, Castagnoli J, Barresi E, Castellano S, Da Settimo F, Taliani S: TSPO Radioligands for Neuroinflammation: An Overview. Molecules 2024;29:4212. 10.3390/molecules29174212 PMC11397380 39275061

[253] Zhang PF, Hu H, Tan L, Yu JT. Microglia Biomarkers in Alzheimer’s Disease. Mol Neurobiol. 2021;58:3388–404. 33713018 10.1007/s12035-021-02348-3

[254] Rauchmann BS, Schneider-Axmann T, Alexopoulos P, Perneczky R. CSF soluble TREM2 as a measure of immune response along the Alzheimer’s disease continuum. Neurobiol Aging. 2019;74:182–90. 30458365 10.1016/j.neurobiolaging.2018.10.022 PMC6331262

[255] Molinuevo JL, Ayton S, Batrla R, Bednar MM, Bittner T, Cummings J, Fagan AM, Hampel H, Mielke MM, Mikulskis A, et al. Current state of Alzheimer’s fluid biomarkers. Acta Neuropathol. 2018;136:821–53. 30488277 10.1007/s00401-018-1932-x PMC6280827

[256] Kamphuis W, Middeldorp J, Kooijman L, Sluijs JA, Kooi EJ, Moeton M, Freriks M, Mizee MR, Hol EM. Glial fibrillary acidic protein isoform expression in plaque related astrogliosis in Alzheimer’s disease. Neurobiol Aging. 2014;35:492–510. 24269023 10.1016/j.neurobiolaging.2013.09.035

[257] Miller TM, Cudkowicz ME, Genge A, Shaw PJ, Sobue G, Bucelli RC, Chio A, Van Damme P, Ludolph AC, Glass JD, et al. Trial of Antisense Oligonucleotide Tofersen for SOD1 ALS. N Engl J Med. 2022;387:1099–110. 36129998 10.1056/NEJMoa2204705

[258] McCampbell A, Cole T, Wegener AJ, Tomassy GS, Setnicka A, Farley BJ, Schoch KM, Hoye ML, Shabsovich M, Sun L, et al. Antisense oligonucleotides extend survival and reverse decrement in muscle response in ALS models. J Clin Invest. 2018;128:3558–67. 30010620 10.1172/JCI99081 PMC6063493

[259] Winer L, Srinivasan D, Chun S, Lacomis D, Jaffa M, Fagan A, Holtzman DM, Wancewicz E, Bennett CF, Bowser R, et al. SOD1 in cerebral spinal fluid as a pharmacodynamic marker for antisense oligonucleotide therapy. JAMA Neurol. 2013;70:201–7. 23147550 10.1001/jamaneurol.2013.593 PMC3812918

[260] Krishnan G, Raitcheva D, Bartlett D, Prudencio M, McKenna-Yasek DM, Douthwright C, Oskarsson BE, Ladha S, King OD, Barmada SJ, et al. Poly(GR) and poly(GA) in cerebrospinal fluid as potential biomarkers for C9ORF72-ALS/FTD. Nat Commun. 2022;13:2799. 35589711 10.1038/s41467-022-30387-4 PMC9119980

[261] Tran H, Moazami MP, Yang H, McKenna-Yasek D, Douthwright CL, Pinto C, Metterville J, Shin M, Sanil N, Dooley C, et al. Suppression of mutant C9orf72 expression by a potent mixed backbone antisense oligonucleotide. Nat Med. 2022;28:117–24. 34949835 10.1038/s41591-021-01557-6 PMC8861976

[262] Ledermann J, Harter P, Gourley C, Friedlander M, Vergote I, Rustin G, Scott C, Meier W, Shapira-Frommer R, Safra T, et al. Olaparib maintenance therapy in platinum-sensitive relapsed ovarian cancer. N Engl J Med. 2012;366:1382–92. 22452356 10.1056/NEJMoa1105535

[263] Sims JR, Zimmer JA, Evans CD, Lu M, Ardayfio P, Sparks J, Wessels AM, Shcherbinin S, Wang H, Monkul Nery ES, et al. Donanemab in Early Symptomatic Alzheimer Disease: The TRAILBLAZER-ALZ 2 Randomized Clinical Trial. JAMA. 2023;330:512–27. 37459141 10.1001/jama.2023.13239 PMC10352931

[264] Roberts SG, Blute ML, Bergstralh EJ, Slezak JM, Zincke H. PSA doubling time as a predictor of clinical progression after biochemical failure following radical prostatectomy for prostate cancer. Mayo Clin Proc. 2001;76:576–81. 11393495 10.4065/76.6.576

[265] Zetterberg H, Skillbäck T, Mattsson N, Trojanowski JQ, Portelius E, Shaw LM, Weiner MW, Blennow K. Association of Cerebrospinal Fluid Neurofilament Light Concentration With Alzheimer Disease Progression. JAMA Neurol. 2016;73:60–7. 26524180 10.1001/jamaneurol.2015.3037 PMC5624219

[266] Palmqvist S, Tideman P, Cullen N, Zetterberg H, Blennow K, Dage JL, Stomrud E, Janelidze S, Mattsson-Carlgren N, Hansson O. Prediction of future Alzheimer’s disease dementia using plasma phospho-tau combined with other accessible measures. Nat Med. 2021;27:1034–42. 34031605 10.1038/s41591-021-01348-z

[267] Janelidze S, Mattsson N, Palmqvist S, Smith R, Beach TG, Serrano GE, Chai X, Proctor NK, Eichenlaub U, Zetterberg H, et al. Plasma P-tau181 in Alzheimer’s disease: relationship to other biomarkers, differential diagnosis, neuropathology and longitudinal progression to Alzheimer’s dementia. Nat Med. 2020;26:379–86. 32123385 10.1038/s41591-020-0755-1

[268] Zhao A, Jiao Y, Ye G, Kang W, Tan L, Li Y, Deng Y, Liu J. Soluble TREM2 levels associate with conversion from mild cognitive impairment to Alzheimer’s disease. J Clin Inv. 2022;132:e158708. 10.1172/JCI158708 PMC9753995 36519540

[269] Ebenau JL, Pelkmans W, Verberk IMW, Verfaillie SCJ, van den Bosch KA, van Leeuwenstijn M, Collij LE, Scheltens P, Prins ND, Barkhof F, et al. Association of CSF, Plasma, and Imaging Markers of Neurodegeneration With Clinical Progression in People With Subjective Cognitive Decline. Neurology. 2022;98:e1315–26. 35110378 10.1212/WNL.0000000000200035 PMC8967429

[270] Tarawneh R, Lee JM, Ladenson JH, Morris JC, Holtzman DM. CSF VILIP-1 predicts rates of cognitive decline in early Alzheimer disease. Neurology. 2012;78:709–19. 22357717 10.1212/WNL.0b013e318248e568 PMC3306162

[271] Kester MI, Teunissen CE, Sutphen C, Herries EM, Ladenson JH, Xiong C, Scheltens P, van der Flier WM, Morris JC, Holtzman DM, Fagan AM. Cerebrospinal fluid VILIP-1 and YKL-40, candidate biomarkers to diagnose, predict and monitor Alzheimer’s disease in a memory clinic cohort. Alzheimers Res Ther. 2015;7:59. 26383836 10.1186/s13195-015-0142-1 PMC4574487

[272] Brookmeyer R, Abdalla N. Estimation of lifetime risks of Alzheimer’s disease dementia using biomarkers for preclinical disease. Alzheimer’s & dementia : the journal of the Alzheimer’s Association. 2018;14:981–8. 10.1016/j.jalz.2018.03.005 PMC6097953 29802030

[273] Pedersen CC, Ushakova A, Alves G, Tysnes O-B, Blennow K, Zetterberg H, Maple-Grødem J, Lange J. Serum neurofilament light at diagnosis: a prognostic indicator for accelerated disease progression in Parkinson’s Disease. npj Parkinson’s Disease. 2024;10:162. 39164268 10.1038/s41531-024-00768-1 PMC11336184

[274] Che N, Ou R, Li C, Zhang L, Wei Q, Wang S, Jiang Q, Yang T, Xiao Y, Lin J, et al. Plasma GFAP as a prognostic biomarker of motor subtype in early Parkinson’s disease. npj Parkinson’s Disease. 2024;10:48. 38429295 10.1038/s41531-024-00664-8 PMC10907600

[275] Lu CH, Macdonald-Wallis C, Gray E, Pearce N, Petzold A, Norgren N, Giovannoni G, Fratta P, Sidle K, Fish M, et al. Neurofilament light chain: A prognostic biomarker in amyotrophic lateral sclerosis. Neurology. 2015;84:2247–57. 25934855 10.1212/WNL.0000000000001642 PMC4456658

[276] Abu-Rumeileh S, Vacchiano V, Zenesini C, Polischi B, de Pasqua S, Fileccia E, Mammana A, Di Stasi V, Capellari S, Salvi F, et al. Diagnostic-prognostic value and electrophysiological correlates of CSF biomarkers of neurodegeneration and neuroinflammation in amyotrophic lateral sclerosis. J Neurol. 2020;267:1699–708. 32100123 10.1007/s00415-020-09761-z

[277] Thouvenot E, Demattei C, Lehmann S, Maceski-Maleska A, Hirtz C, Juntas-Morales R, Pageot N, Esselin F, Alphandery S, Vincent T, Camu W. Serum neurofilament light chain at time of diagnosis is an independent prognostic factor of survival in amyotrophic lateral sclerosis. Eur J Neurol. 2020;27:251–7. 31437330 10.1111/ene.14063

[278] Vacchiano V, Mastrangelo A, Zenesini C, Masullo M, Quadalti C, Avoni P, Polischi B, Cherici A, Capellari S, Salvi F, et al. Plasma and CSF Neurofilament Light Chain in Amyotrophic Lateral Sclerosis: A Cross-Sectional and Longitudinal Study. Front Aging Neurosci. 2021;13: 753242. 34744694 10.3389/fnagi.2021.753242 PMC8569186

[279] Zecca C, Dell’Abate MT, Pasculli G, Capozzo R, Barone R, Arima S, Pollice A, Brescia V, Tortelli R, Logroscino G. Role of plasma phosphorylated neurofilament heavy chain (pNfH) in amyotrophic lateral sclerosis. J Cell Mol Med. 2022;26:3608–15. 35715961 10.1111/jcmm.17232 PMC9258711

[280] Falzone YM, Domi T, Agosta F, Pozzi L, Schito P, Fazio R, Del Carro U, Barbieri A, Comola M, Leocani L, et al. Serum phosphorylated neurofilament heavy-chain levels reflect phenotypic heterogeneity and are an independent predictor of survival in motor neuron disease. J Neurol. 2020;267:2272–80. 32306171 10.1007/s00415-020-09838-9 PMC7166001

[281] Ferrell PB Jr, McLeod HL. Carbamazepine, HLA-B*1502 and risk of Stevens-Johnson syndrome and toxic epidermal necrolysis: US FDA recommendations. Pharmacogenomics. 2008;9:1543–6. 18855540 10.2217/14622416.9.10.1543 PMC2586963

[282] Sveikata L, Charidimou A, Viswanathan A. Vessels Sing Their ARIAs: The Role of Vascular Amyloid in the Age of Aducanumab. Stroke. 2022;53:298–302. 34905943 10.1161/STROKEAHA.121.036873

[283] Sin MK, Zamrini E, Ahmed A, Nho K, Hajjar I. Anti-Amyloid Therapy, AD, and ARIA: Untangling the Role of CAA. J Clin Med 2023;12:6792. 10.3390/jcm12216792 PMC10647766 37959255

[284] Chartier-Harlin MC, Parfitt M, Legrain S, Perez-Tur J, Brousseau T, Evans A, Berr C, Vidal O, Roques P, Gourlet V, et al. Apolipoprotein E, epsilon 4 allele as a major risk factor for sporadic early and late-onset forms of Alzheimer’s disease: analysis of the 19q13.2 chromosomal region. Hum Mol Genet. 1994;3:569–74. 8069300 10.1093/hmg/3.4.569

[285] Genin E, Hannequin D, Wallon D, Sleegers K, Hiltunen M, Combarros O, Bullido MJ, Engelborghs S, De Deyn P, Berr C, et al. APOE and Alzheimer disease: a major gene with semi-dominant inheritance. Mol Psychiatry. 2011;16:903–7. 21556001 10.1038/mp.2011.52 PMC3162068

[286] Farrer LA, Cupples LA, Haines JL, Hyman B, Kukull WA, Mayeux R, Myers RH, Pericak-Vance MA, Risch N, van Duijn CM. Effects of age, sex, and ethnicity on the association between apolipoprotein E genotype and Alzheimer disease. A meta-analysis. APOE and Alzheimer Disease Meta Analysis Consortium. Jama 1997, 278:1349–1356. 9343467

[287] Kweon SH, Ryu HG, Park H, Lee S, Kim N, Kwon SH, Ma SX, Kim S, Ko HS. Linking Gba1 E326K mutation to microglia activation and mild age-dependent dopaminergic Neurodegeneration. bioRxiv 2024.

[288] Benatar M, Wuu J, Andersen PM, Lombardi V, Malaspina A. Neurofilament light: A candidate biomarker of presymptomatic amyotrophic lateral sclerosis and phenoconversion. Ann Neurol. 2018;84:130–9. 30014505 10.1002/ana.25276 PMC11348288

[289] van der Ende EL, Meeter LH, Poos JM, Panman JL, Jiskoot LC, Dopper EGP, Papma JM, de Jong FJ, Verberk IMW, Teunissen C, et al. Serum neurofilament light chain in genetic frontotemporal dementia: a longitudinal, multicentre cohort study. Lancet Neurol. 2019;18:1103–11. 31701893 10.1016/S1474-4422(19)30354-0

[290] De Schaepdryver M, Goossens J, De Meyer S, Jeromin A, Masrori P, Brix B, Claeys KG, Schaeverbeke J, Adamczuk K, Vandenberghe R, et al. Serum neurofilament heavy chains as early marker of motor neuron degeneration. Ann Clin Transl Neurol. 2019;6:1971–9. 31518073 10.1002/acn3.50890 PMC6801162

[291] Abati E, Bresolin N, Comi G, Corti S. Silence superoxide dismutase 1 (SOD1): a promising therapeutic target for amyotrophic lateral sclerosis (ALS). Expert Opin Ther Targets. 2020;24:295–310. 32125907 10.1080/14728222.2020.1738390

[292] DeJesus-Hernandez M, Mackenzie IR, Boeve BF, Boxer AL, Baker M, Rutherford NJ, Nicholson AM, Finch NA, Flynn H, Adamson J, et al. Expanded GGGGCC hexanucleotide repeat in noncoding region of C9ORF72 causes chromosome 9p-linked FTD and ALS. Neuron. 2011;72:245–56. 21944778 10.1016/j.neuron.2011.09.011 PMC3202986

[293] Renton AE, Majounie E, Waite A, Simon-Sanchez J, Rollinson S, Gibbs JR, Schymick JC, Laaksovirta H, van Swieten JC, Myllykangas L, et al. A hexanucleotide repeat expansion in C9ORF72 is the cause of chromosome 9p21-linked ALS-FTD. Neuron. 2011;72:257–68. 21944779 10.1016/j.neuron.2011.09.010 PMC3200438

[294] Elden AC, Kim HJ, Hart MP, Chen-Plotkin AS, Johnson BS, Fang X, Armakola M, Geser F, Greene R, Lu MM, et al. Ataxin-2 intermediate-length polyglutamine expansions are associated with increased risk for ALS. Nature. 2010;466:1069–75. 20740007 10.1038/nature09320 PMC2965417

[295] Bakker E, Hendrikse NM, Ehmann F, van der Meer DS, Llinares Garcia J, Vetter T, Starokozhko V, Mol PGM. Biomarker Qualification at the European Medicines Agency: A Review of Biomarker Qualification Procedures From 2008 to 2020. Clin Pharmacol Ther. 2022;112:69–80. 35137949 10.1002/cpt.2554 PMC9313861

[296] McMackin R, Bede P, Ingre C, Malaspina A, Hardiman O. Biomarkers in amyotrophic lateral sclerosis: current status and future prospects. Nat Rev Neurol. 2023;19:754–68. 37949994 10.1038/s41582-023-00891-2

[297] Horie K, Salvado G, Barthelemy NR, Janelidze S, Li Y, He Y, Saef B, Chen CD, Jiang H, Strandberg O, et al. CSF MTBR-tau243 is a specific biomarker of tau tangle pathology in Alzheimer’s disease. Nat Med. 2023;29:1954–63. 37443334 10.1038/s41591-023-02443-z PMC10427417

[298] Abu-Rumeileh S, Steinacker P, Polischi B, Mammana A, Bartoletti-Stella A, Oeckl P, Baiardi S, Zenesini C, Huss A, Cortelli P, et al. CSF biomarkers of neuroinflammation in distinct forms and subtypes of neurodegenerative dementia. Alzheimers Res Ther. 2019;12:2. 31892365 10.1186/s13195-019-0562-4 PMC6937795

[299] Agnello L, Gambino CM, Ciaccio AM, Salemi G, Brighina F, Ragonese P, Piccoli T, Blandino V, Di Stefano V, Cacciabaudo F, et al. The value of serum glial fibrillary acidic protein as a biomarker of astrogliosis in different neurological diseases. Clin Chim Acta. 2025;572: 120248. 40113024 10.1016/j.cca.2025.120248

[300] Grillo P, Riboldi GM, Pisani A, Kang UJ, Fereshtehnejad SM: Amplification parameters of the alpha-synuclein seed amplification assay on CSF predict the clinical subtype of Parkinson’s Disease at 10-year follow-up. medRxiv 2025. 10.1177/1877718X261417468 PMC13347583 41610263

[301] Grossauer A, Hemicker G, Krismer F, Peball M, Djamshidian A, Poewe W, Seppi K, Heim B. alpha-Synuclein Seed Amplification Assays in the Diagnosis of Synucleinopathies Using Cerebrospinal Fluid-A Systematic Review and Meta-Analysis. Mov Disord Clin Pract. 2023;10:737–47. 37205253 10.1002/mdc3.13710 PMC10187020

[302] Irwin KE, Jasin P, Braunstein KE, Sinha IR, Garret MA, Bowden KD, Chang K, Troncoso JC, Moghekar A, Oh ES, et al. A fluid biomarker reveals loss of TDP-43 splicing repression in presymptomatic ALS-FTD. Nat Med. 2024;30:382–93. 38278991 10.1038/s41591-023-02788-5 PMC10878965

[303] Cordts I, Wachinger A, Scialo C, Lingor P, Polymenidou M, Buratti E, Feneberg E. TDP-43 Proteinopathy Specific Biomarker Development. Cells 2023;12:597. 10.3390/cells12040597 PMC9954136 36831264

[304] Nilsson J, Pichet Binette A, Palmqvist S, Brum WS, Janelidze S, Ashton NJ, Spotorno N, Stomrud E, Gobom J, Zetterberg H, et al. Cerebrospinal fluid biomarker panel for synaptic dysfunction in a broad spectrum of neurodegenerative diseases. Brain. 2024;147:2414–27. 38325331 10.1093/brain/awae032 PMC11224614

[305] Pesamaa I, Muller SA, Robinson S, Darcher A, Paquet D, Zetterberg H, Lichtenthaler SF, Haass C. A microglial activity state biomarker panel differentiates FTD-granulin and Alzheimer’s disease patients from controls. Mol Neurodegener. 2023;18:70. 37775827 10.1186/s13024-023-00657-w PMC10543321

[306] Suarez-Calvet M, Karikari TK, Ashton NJ, Lantero Rodriguez J, Mila-Aloma M, Gispert JD, Salvado G, Minguillon C, Fauria K, Shekari M, et al. Novel tau biomarkers phosphorylated at T181, T217 or T231 rise in the initial stages of the preclinical Alzheimer’s continuum when only subtle changes in Abeta pathology are detected. EMBO Mol Med. 2020;12: e12921. 33169916 10.15252/emmm.202012921 PMC7721364

[307] Lin YS, Kwon HS, Lee WJ, Hwang M, Jeong JH, Koh SH, Choi SH, Fuh JL. Cross-cultural validation of plasma p-tau217 and p-tau181 as precision biomarkers for amyloid PET positivity: An East Asian study in Taiwan and Korea. Alzheimers Dement. 2025;21: e14565. 39877979 10.1002/alz.14565 PMC11775528

[308] Schindler SE, Petersen KK, Saef B, Tosun D, Shaw LM, Zetterberg H, Dage JL, Ferber K, Triana-Baltzer G, Du-Cuny L, et al. Head-to-head comparison of leading blood tests for Alzheimer’s disease pathology. Alzheimers Dement. 2024;20:8074–96. 39394841 10.1002/alz.14315 PMC11567821

[309] Barthelemy NR, Salvado G, Schindler SE, He Y, Janelidze S, Collij LE, Saef B, Henson RL, Chen CD, Gordon BA, et al. Highly accurate blood test for Alzheimer’s disease is similar or superior to clinical cerebrospinal fluid tests. Nat Med. 2024;30:1085–95. 38382645 10.1038/s41591-024-02869-z PMC11031399

[310] Kwon HS, Kim JY, Koh SH, Choi SH, Lee EH, Jeong JH, Jang JW, Park KW, Kim EJ, Hong JY, et al. Predicting cognitive stage transition using p-tau181, Centiloid, and other measures. Alzheimers Dement. 2023;19:4641–50. 36988152 10.1002/alz.13054 PMC12009171

[311] Kwon HS, Hwang M, Koh SH, Choi SH, Lee JH, Kim HJ, Park SH, Park HH, Jeong JH, Han MH, Kim JY. Comparison of plasma p-tau217 and p-tau181 in predicting amyloid positivity and prognosis among Korean memory clinic patients. Sci Rep. 2025;15:7791. 40044785 10.1038/s41598-025-90232-8 PMC11882798

[312] Ashton NJ, Janelidze S, Mattsson-Carlgren N, Binette AP, Strandberg O, Brum WS, Karikari TK, Gonzalez-Ortiz F, Di Molfetta G, Meda FJ, et al. Differential roles of Abeta42/40, p-tau231 and p-tau217 for Alzheimer’s trial selection and disease monitoring. Nat Med. 2022;28:2555–62. 36456833 10.1038/s41591-022-02074-w PMC9800279

[313] Therriault J, Vermeiren M, Servaes S, Tissot C, Ashton NJ, Benedet AL, Karikari TK, Lantero-Rodriguez J, Brum WS, Lussier FZ, et al. Association of Phosphorylated Tau Biomarkers With Amyloid Positron Emission Tomography vs Tau Positron Emission Tomography. JAMA Neurol. 2023;80:188–99. 36508198 10.1001/jamaneurol.2022.4485 PMC9856704

[314] Horie K, Salvado G, Koppisetti RK, Janelidze S, Barthelemy NR, He Y, Sato C, Gordon BA, Jiang H, Benzinger TLS, et al. Plasma MTBR-tau243 biomarker identifies tau tangle pathology in Alzheimer’s disease. Nat Med 2025. 10.1038/s41591-025-03617-7 PMC12176612 40164726

[315] Khalil M, Teunissen CE, Otto M, Piehl F, Sormani MP, Gattringer T, Barro C, Kappos L, Comabella M, Fazekas F, et al. Neurofilaments as biomarkers in neurological disorders. Nat Rev Neurol. 2018;14:577–89. 30171200 10.1038/s41582-018-0058-z

[316] Lambertsen KL, Soares CB, Gaist D, Nielsen HH. Neurofilaments: The C-Reactive Protein of Neurology. Brain sciences 2020;10:56. 10.3390/brainsci10010056 PMC7016784 31963750

[317] Lee E-H, Kwon HS, Koh S-H, Choi SH, Jin J-H, Jeong JH, Jang J-W, Park KW, Kim E-J, Kim HJ, et al. Serum neurofilament light chain level as a predictor of cognitive stage transition. Alzheimer’s Research & Therapy. 2022;14:6. 10.1186/s13195-021-00953-x PMC8742445 34996525

[318] Benatar M, Wuu J, Andersen PM, Bucelli RC, Andrews JA, Otto M, Farahany NA, Harrington EA, Chen W, Mitchell AA, et al. Design of a Randomized, Placebo-Controlled, Phase 3 Trial of Tofersen Initiated in Clinically Presymptomatic SOD1 Variant Carriers: the ATLAS Study. Neurotherapeutics. 2022;19:1248–58. 35585374 10.1007/s13311-022-01237-4 PMC9587202

[319] Beltran TA. Normative Values for Serum Neurofilament Light Chain in US Adults. J Clin Neurol. 2024;20:46–9. 38179631 10.3988/jcn.2022.0340 PMC10782095

[320] Manouchehrinia A, Piehl F, Hillert J, Kuhle J, Alfredsson L, Olsson T, Kockum I. Confounding effect of blood volume and body mass index on blood neurofilament light chain levels. Ann Clin Transl Neurol. 2020;7:139–43. 31893563 10.1002/acn3.50972 PMC6952306

[321] Witzel S, Frauhammer F, Steinacker P, Devos D, Pradat PF, Meininger V, Halbgebauer S, Oeckl P, Schuster J, Anders S, et al. Neurofilament light and heterogeneity of disease progression in amyotrophic lateral sclerosis: development and validation of a prediction model to improve interventional trials. Transl Neurodegener. 2021;10:31. 34433481 10.1186/s40035-021-00257-y PMC8390195

[322] Magen I, Yacovzada NS, Yanowski E, Coenen-Stass A, Grosskreutz J, Lu CH, Greensmith L, Malaspina A, Fratta P, Hornstein E. Circulating miR-181 is a prognostic biomarker for amyotrophic lateral sclerosis. Nat Neurosci. 2021;24:1534–41. 34711961 10.1038/s41593-021-00936-z

[323] Gendron TF, Chew J, Stankowski JN, Hayes LR, Zhang YJ, Prudencio M, Carlomagno Y, Daughrity LM, Jansen-West K, Perkerson EA, et al. Poly(GP) proteins are a useful pharmacodynamic marker for C9ORF72-associated amyotrophic lateral sclerosis. Sci Transl Med 2017;9:eaai7886. 10.1126/scitranslmed.aai7866 PMC5576451 28356511

[324] Bellaver B, Povala G, Ferreira PCL, Ferrari-Souza JP, Leffa DT, Lussier FZ, Benedet AL, Ashton NJ, Triana-Baltzer G, Kolb HC, et al. Astrocyte reactivity influences amyloid-beta effects on tau pathology in preclinical Alzheimer’s disease. Nat Med. 2023;29:1775–81. 37248300 10.1038/s41591-023-02380-x PMC10353939

[325] Lin J, Ou R, Li C, Hou Y, Zhang L, Wei Q, Pang D, Liu K, Jiang Q, Yang T, et al. Plasma glial fibrillary acidic protein as a biomarker of disease progression in Parkinson’s disease: a prospective cohort study. BMC Med. 2023;21:420. 37932720 10.1186/s12916-023-03120-1 PMC10626747

[326] Verde F, Milone I, Maranzano A, Colombo E, Torre S, Solca F, Doretti A, Gentile F, Manini A, Bonetti R, et al. Serum levels of glial fibrillary acidic protein in patients with amyotrophic lateral sclerosis. Ann Clin Transl Neurol. 2023;10:118–29. 36525477 10.1002/acn3.51708 PMC9852391

[327] Neumann M, Sampathu DM, Kwong LK, Truax AC, Micsenyi MC, Chou TT, Bruce J, Schuck T, Grossman M, Clark CM, et al. Ubiquitinated TDP-43 in frontotemporal lobar degeneration and amyotrophic lateral sclerosis. Science. 2006;314:130–3. 17023659 10.1126/science.1134108

[328] Nelson PT, Dickson DW, Trojanowski JQ, Jack CR, Boyle PA, Arfanakis K, Rademakers R, Alafuzoff I, Attems J, Brayne C, et al. Limbic-predominant age-related TDP-43 encephalopathy (LATE): consensus working group report. Brain. 2019;142:1503–27. 31039256 10.1093/brain/awz099 PMC6536849

[329] Meneses A, Koga S, O’Leary J, Dickson DW, Bu G, Zhao N. TDP-43 Pathology in Alzheimer’s Disease. Mol Neurodegener. 2021;16:84. 34930382 10.1186/s13024-021-00503-x PMC8691026

[330] Noto Y, Shibuya K, Sato Y, Kanai K, Misawa S, Sawai S, Mori M, Uchiyama T, Isose S, Nasu S, et al. Elevated CSF TDP-43 levels in amyotrophic lateral sclerosis: specificity, sensitivity, and a possible prognostic value. Amyotroph Lateral Scler. 2011;12:140–3. 21126161 10.3109/17482968.2010.541263

[331] Kasai T, Tokuda T, Ishigami N, Sasayama H, Foulds P, Mitchell DJ, Mann DM, Allsop D, Nakagawa M. Increased TDP-43 protein in cerebrospinal fluid of patients with amyotrophic lateral sclerosis. Acta Neuropathol. 2009;117:55–62. 18989684 10.1007/s00401-008-0456-1

[332] Majumder V, Gregory JM, Barria MA, Green A, Pal S. TDP-43 as a potential biomarker for amyotrophic lateral sclerosis: a systematic review and meta-analysis. BMC Neurol. 2018;18:90. 29954341 10.1186/s12883-018-1091-7 PMC6027783

[333] Irwin KE, Sheth U, Wong PC, Gendron TF. Fluid biomarkers for amyotrophic lateral sclerosis: a review. Mol Neurodegener. 2024;19:9. 38267984 10.1186/s13024-023-00685-6 PMC10809579

[334] Ling JP, Pletnikova O, Troncoso JC, Wong PC. TDP-43 repression of nonconserved cryptic exons is compromised in ALS-FTD. Science. 2015;349:650–5. 26250685 10.1126/science.aab0983 PMC4825810

[335] Mehta PR, Brown AL, Ward ME, Fratta P. The era of cryptic exons: implications for ALS-FTD. Mol Neurodegener. 2023;18:16. 36922834 10.1186/s13024-023-00608-5 PMC10018954

[336] Seddighi S, Qi YA, Brown AL, Wilkins OG, Bereda C, Belair C, Zhang YJ, Prudencio M, Keuss MJ, Khandeshi A, et al. Mis-spliced transcripts generate de novo proteins in TDP-43-related ALS/FTD. Sci Transl Med. 2024;16:eadg7162. 38277467 10.1126/scitranslmed.adg7162 PMC11325748

[337] Calliari A, Daughrity LM, Albagli EA, Castellanos Otero P, Yue M, Jansen-West K, Islam NN, Caulfield T, Rawlinson B, DeTure M, et al. HDGFL2 cryptic proteins report presence of TDP-43 pathology in neurodegenerative diseases. Mol Neurodegener. 2024;19:29. 38539264 10.1186/s13024-024-00718-8 PMC10967196

[338] Johnson ECB, Bian S, Haque RU, Carter EK, Watson CM, Gordon BA, Ping L, Duong DM, Epstein MP, McDade E, et al. Cerebrospinal fluid proteomics define the natural history of autosomal dominant Alzheimer’s disease. Nat Med. 2023;29:1979–88. 37550416 10.1038/s41591-023-02476-4 PMC10427428

[339] Matuskey D, Tinaz S, Wilcox KC, Naganawa M, Toyonaga T, Dias M, Henry S, Pittman B, Ropchan J, Nabulsi N, et al. Synaptic Changes in Parkinson Disease Assessed with in vivo Imaging. Ann Neurol. 2020;87:329–38. 31953875 10.1002/ana.25682 PMC7065227

[340] Mecca AP, Chen MK, O’Dell RS, Naganawa M, Toyonaga T, Godek TA, Harris JE, Bartlett HH, Zhao W, Nabulsi NB, et al. In vivo measurement of widespread synaptic loss in Alzheimer’s disease with SV2A PET. Alzheimers Dement. 2020;16:974–82. 32400950 10.1002/alz.12097 PMC7383876

[341] Hansson O. Biomarkers for neurodegenerative diseases. Nat Med. 2021;27:954–63. 34083813 10.1038/s41591-021-01382-x

[342] Ng SC, de la Monte SM, Conboy GL, Karns LR, Fishman MC. Cloning of human GAP-43: growth association and ischemic resurgence. Neuron. 1988;1:133–9. 3272163 10.1016/0896-6273(88)90197-3

[343] Represa A, Deloulme JC, Sensenbrenner M, Ben-Ari Y, Baudier J. Neurogranin: immunocytochemical localization of a brain-specific protein kinase C substrate. J Neurosci. 1990;10:3782–92. 2269883 10.1523/JNEUROSCI.10-12-03782.1990 PMC6570038

[344] Serrano-Pozo A, Frosch MP, Masliah E, Hyman BT. Neuropathological alterations in Alzheimer disease. Cold Spring Harb Perspect Med. 2011;1: a006189. 22229116 10.1101/cshperspect.a006189 PMC3234452

[345] Ulland TK, Colonna M. TREM2 - a key player in microglial biology and Alzheimer disease. Nat Rev Neurol. 2018;14:667–75. 30266932 10.1038/s41582-018-0072-1

[346] Gratuze M, Chen Y, Parhizkar S, Jain N, Strickland MR, Serrano JR, Colonna M, Ulrich JD, Holtzman DM: Activated microglia mitigate Aβ-associated tau seeding and spreading. J Exp Med. 2021;218:e20210542. 10.1084/jem.20210542 PMC8190588 34100905

[347] Nabizadeh F, Seyedmirzaei H, Karami S. Neuroimaging biomarkers and CSF sTREM2 levels in Alzheimer’s disease: a longitudinal study. Sci Rep. 2024;14:15318. 38961148 10.1038/s41598-024-66211-w PMC11222555

[348] Hu WT, Ozturk T, Kollhoff A, Wharton W, Christina Howell J. Higher CSF sTNFR1-related proteins associate with better prognosis in very early Alzheimer’s disease. Nat Commun. 2021;12:4001. 34183654 10.1038/s41467-021-24220-7 PMC8238986

[349] Schlepckow K, Morenas-Rodríguez E, Hong S, Haass C. Stimulation of TREM2 with agonistic antibodies-an emerging therapeutic option for Alzheimer’s disease. The Lancet Neurology. 2023;22:1048–60. 37863592 10.1016/S1474-4422(23)00247-8

[350] Zhang X, Zhong X, Wang L, Li H, Yang L, Li X, Yu X, Xie A. Effects of soluble TREM2 on motor progression in Parkinson’s disease. Neurosci Lett. 2023;807: 137277. 37105353 10.1016/j.neulet.2023.137277

[351] Jiao L, Yang J, Wang W, Liu X, Fu Y, Fan D. sTREM2 cerebrospinal fluid levels are a potential biomarker in amyotrophic lateral sclerosis and associate with UMN burden. Front Neurol. 2024;15:1515252. 39722698 10.3389/fneur.2024.1515252 PMC11669252

[352] Batzu L, Westman E, Pereira JB. Cerebrospinal fluid progranulin is associated with increased cortical thickness in early stages of Alzheimer’s disease. Neurobiol Aging. 2020;88:61–70. 31980280 10.1016/j.neurobiolaging.2019.12.012

[353] Morenas-Rodriguez E, Cervera-Carles L, Vilaplana E, Alcolea D, Carmona-Iragui M, Dols-Icardo O, Ribosa-Nogue R, Munoz-Llahuna L, Sala I, Belen Sanchez-Saudinos M, et al. Progranulin Protein Levels in Cerebrospinal Fluid in Primary Neurodegenerative Dementias. J Alzheimers Dis. 2016;50:539–46. 26682689 10.3233/JAD-150746

[354] Philips T, De Muynck L, Thu HN, Weynants B, Vanacker P, Dhondt J, Sleegers K, Schelhaas HJ, Verbeek M, Vandenberghe R, et al. Microglial upregulation of progranulin as a marker of motor neuron degeneration. J Neuropathol Exp Neurol. 2010;69:1191–200. 21107132 10.1097/NEN.0b013e3181fc9aea

[355] Perea JR, Lleo A, Alcolea D, Fortea J, Avila J, Bolos M. Decreased CX3CL1 Levels in the Cerebrospinal Fluid of Patients With Alzheimer’s Disease. Front Neurosci. 2018;12:609. 30245615 10.3389/fnins.2018.00609 PMC6137321

[356] Llorens F, Thune K, Tahir W, Kanata E, Diaz-Lucena D, Xanthopoulos K, Kovatsi E, Pleschka C, Garcia-Esparcia P, Schmitz M, et al. YKL-40 in the brain and cerebrospinal fluid of neurodegenerative dementias. Mol Neurodegener. 2017;12:83. 29126445 10.1186/s13024-017-0226-4 PMC5681777

[357] Baldacci F, Lista S, Palermo G, Giorgi FS, Vergallo A, Hampel H. The neuroinflammatory biomarker YKL-40 for neurodegenerative diseases: advances in development. Expert Rev Proteomics. 2019;16:593–600. 31195846 10.1080/14789450.2019.1628643

[358] Pinteac R, Montalban X, Comabella M: Chitinases and chitinase-like proteins as biomarkers in neurologic disorders. Neurol Neuroimmunol Neuroinflamm 2021;8:e921. 10.1212/NXI.0000000000000921 PMC7803328 33293459

[359] Russo C, Valle MS, Casabona A, Malaguarnera L. Chitinase Signature in the Plasticity of Neurodegenerative Diseases. Int J Mol Sci 2023;24:6301. 10.3390/ijms24076301 PMC10094409 37047273

[360] Belien J, Swinnen S, D’Hondt R. Verdu de Juan L, Dedoncker N, Matthys P, Bauer J, Vens C, Moylett S, Dubois B: CHIT1 at diagnosis predicts faster disability progression and reflects early microglial activation in multiple sclerosis. Nat Commun. 2024;15:5013. 38866782 10.1038/s41467-024-49312-y PMC11169395

[361] Budge KM, Neal ML, Richardson JR, Safadi FF. Glycoprotein NMB: an Emerging Role in Neurodegenerative Disease. Mol Neurobiol. 2018;55:5167–76. 28856541 10.1007/s12035-017-0707-z

[362] Huttenrauch M, Ogorek I, Klafki H, Otto M, Stadelmann C, Weggen S, Wiltfang J, Wirths O. Glycoprotein NMB: a novel Alzheimer’s disease associated marker expressed in a subset of activated microglia. Acta Neuropathol Commun. 2018;6:108. 30340518 10.1186/s40478-018-0612-3 PMC6194687

[363] Zhu XC, Mizutani Y, Ohdake R, Tatebe H, Maeda T, Shima S, Ueda A, Ito M, Ito S, Tokuda T, Watanabe H. CSF GPNMB in Parkinson’s disease: A potential association with age and microglial activation. J Parkinsons Dis. 2024;14:1533–42. 39957200 10.1177/1877718X241288712 PMC13348315

[364] Hansson O, Edelmayer RM, Boxer AL, Carrillo MC, Mielke MM, Rabinovici GD, Salloway S, Sperling R, Zetterberg H, Teunissen CE. The Alzheimer’s Association appropriate use recommendations for blood biomarkers in Alzheimer’s disease. Alzheimers Dement. 2022;18:2669–86. 35908251 10.1002/alz.12756 PMC10087669

[365] Kim DK, Han D, Park J, Choi H, Park JC, Cha MY, Woo J, Byun MS, Lee DY, Kim Y, Mook-Jung I. Deep proteome profiling of the hippocampus in the 5XFAD mouse model reveals biological process alterations and a novel biomarker of Alzheimer’s disease. Exp Mol Med. 2019;51:1–17. 31727875 10.1038/s12276-019-0326-z PMC6856180

[366] Shafit-Zagardo B, Gruber RC, DuBois JC. The role of TAM family receptors and ligands in the nervous system: From development to pathobiology. Pharmacol Ther. 2018;188:97–117. 29514053 10.1016/j.pharmthera.2018.03.002 PMC6067981

[367] Li L, Ren J, Pan C, Li Y, Xu J, Dong H, Chen Y, Liu W. Serum miR-214 Serves as a Biomarker for Prodromal Parkinson’s Disease. Front Aging Neurosci. 2021;13: 700959. 34776924 10.3389/fnagi.2021.700959 PMC8581655

[368] Largeau B, Dupont AC, Guilloteau D, Santiago-Ribeiro MJ, Arlicot N. TSPO PET Imaging: From Microglial Activation to Peripheral Sterile Inflammatory Diseases? Contrast Media Mol Imaging. 2017;2017:6592139. 29114179 10.1155/2017/6592139 PMC5632884

[369] De Picker LJ, Morrens M, Branchi I, Haarman BCM, Terada T, Kang MS, Boche D, Tremblay ME, Leroy C, Bottlaender M, Ottoy J. TSPO PET brain inflammation imaging: A transdiagnostic systematic review and meta-analysis of 156 case-control studies. Brain Behav Immun. 2023;113:415–31. 37543251 10.1016/j.bbi.2023.07.023

[370] Femminella GD, Dani M, Wood M, Fan Z, Calsolaro V, Atkinson R, Edginton T, Hinz R, Brooks DJ, Edison P. Microglial activation in early Alzheimer trajectory is associated with higher gray matter volume. Neurology. 2019;92:e1331–43. 30796139 10.1212/WNL.0000000000007133 PMC6511099

[371] Horti AG, Naik R, Foss CA, Minn I, Misheneva V, Du Y, Wang Y, Mathews WB, Wu Y, Hall A, et al. PET imaging of microglia by targeting macrophage colony-stimulating factor 1 receptor (CSF1R). Proc Natl Acad Sci U S A. 2019;116:1686–91. 30635412 10.1073/pnas.1812155116 PMC6358677

[372] Mills KA, Du Y, Coughlin JM, Foss CA, Horti AG, Jenkins KR, Skorobogatova Y, Spiro E, Motley CS, Dannals RF, et al. Exploring [11C]CPPC as a CSF1R-targeted PET imaging marker for early Parkinson’s disease severity. J Clin Invest. 2025;15;135(12):e186591. 10.1172/JCI186591 PMC12165784 40232849

[373] Kim K, Moore DH, Makriyannis A, Abood ME. AM1241, a cannabinoid CB2 receptor selective compound, delays disease progression in a mouse model of amyotrophic lateral sclerosis. Eur J Pharmacol. 2006;542:100–5. 16781706 10.1016/j.ejphar.2006.05.025

[374] Ni R, Mu L, Ametamey S. Positron emission tomography of type 2 cannabinoid receptors for detecting inflammation in the central nervous system. Acta Pharmacol Sin. 2019;40:351–7. 29921889 10.1038/s41401-018-0035-5 PMC6460366

[375] van der Wildt B, Janssen B, Pekosak A, Steen EJL, Schuit RC, Kooijman EJM, Beaino W, Vugts DJ, Windhorst AD. Novel Thienopyrimidine-Based PET Tracers for P2Y(12) Receptor Imaging in the Brain. ACS Chem Neurosci. 2021;12:4465–74. 34757711 10.1021/acschemneuro.1c00641 PMC8640995

[376] Zhou R, Ji B, Kong Y, Qin L, Ren W, Guan Y, Ni R. PET Imaging of Neuroinflammation in Alzheimer’s Disease. Front Immunol. 2021;12: 739130. 34603323 10.3389/fimmu.2021.739130 PMC8481830

[377] Cummings J, Apostolova L, Rabinovici GD, Atri A, Aisen P, Greenberg S, Hendrix S, Selkoe D, Weiner M, Petersen RC, Salloway S. Lecanemab: Appropriate use recommendations. J Prev Alzheimers Dis. 2023;10:362–77. 37357276 10.14283/jpad.2023.30 PMC10313141

[378] Angelova DM, Brown DR. Altered Processing of beta-Amyloid in SH-SY5Y Cells Induced by Model Senescent Microglia. ACS Chem Neurosci. 2018;9:3137–52. 30052418 10.1021/acschemneuro.8b00334

[379] Zeineh MM, Chen Y, Kitzler HH, Hammond R, Vogel H, Rutt BK. Activated iron-containing microglia in the human hippocampus identified by magnetic resonance imaging in Alzheimer disease. Neurobiol Aging. 2015;36:2483–500. 26190634 10.1016/j.neurobiolaging.2015.05.022 PMC4839298

[380] Taquet M, Jankovski A, Rensonnet G, Jacobs D. des Rieux A, Macq B, Warfield SK, Scherrer B: Extra-axonal restricted diffusion as an in-vivo marker of reactive microglia. Sci Rep. 2019;9:13874. 31554896 10.1038/s41598-019-50432-5 PMC6761095

[381] Yi SY, Barnett BR, Torres-Velazquez M, Zhang Y, Hurley SA, Rowley PA, Hernando D, Yu JJ. Detecting Microglial Density With Quantitative Multi-Compartment Diffusion MRI. Front Neurosci. 2019;13:81. 30837826 10.3389/fnins.2019.00081 PMC6389825

[382] Morgan DG, Mielke MM. Knowledge gaps in Alzheimer’s disease immune biomarker research. Alzheimers Dement. 2021;17:2030–42. 33984178 10.1002/alz.12342 PMC8884450

[383] Dawson TM, Golde TE, Lagier-Tourenne C. Animal models of neurodegenerative diseases. Nat Neurosci. 2018;21:1370–9. 30250265 10.1038/s41593-018-0236-8 PMC6615039

[384] Hasselmann J, Coburn MA, England W, Figueroa Velez DX, Kiani Shabestari S, Tu CH, McQuade A, Kolahdouzan M, Echeverria K, Claes C, et al. Development of a Chimeric Model to Study and Manipulate Human Microglia In Vivo. Neuron. 2019;103(1016–1033): e1010. 10.1016/j.neuron.2019.07.002 PMC7138101 31375314

[385] Abels ER, Nieland L, Hickman S, Broekman MLD, El Khoury J, Maas SLN: Comparative Analysis Identifies Similarities between the Human and Murine Microglial Sensomes. Int J Mol Sci 2021;22:1495. 10.3390/ijms22031495 PMC7867338 33540859

[386] Olah M, Menon V, Habib N, Taga MF, Ma Y, Yung CJ, Cimpean M, Khairallah A, Coronas-Samano G, Sankowski R, et al. Single cell RNA sequencing of human microglia uncovers a subset associated with Alzheimer’s disease. Nat Commun. 2020;11:6129. 33257666 10.1038/s41467-020-19737-2 PMC7704703

[387] Zeng H, Huang J, Zhou H, Meilandt WJ, Dejanovic B, Zhou Y, Bohlen CJ, Lee SH, Ren J, Liu A, et al. Integrative in situ mapping of single-cell transcriptional states and tissue histopathology in a mouse model of Alzheimer’s disease. Nat Neurosci. 2023;26:430–46. 36732642 10.1038/s41593-022-01251-x PMC11332722

[388] Choi H, Lee EJ, Shin JS, Kim H, Bae S, Choi Y, Lee DS. Spatiotemporal characterization of glial cell activation in an Alzheimer’s disease model by spatially resolved transcriptomics. Exp Mol Med. 2023;55:2564–75. 38036733 10.1038/s12276-023-01123-9 PMC10767047

[389] Abdullahi Tunde Aborode a OAEb, Isreal Ayobami Onifade c, Emmanuel Olotu d, Oche Joseph Otorkpa e, Qasim Mehmood f, Suliat Iyabode Abdulai g, Abdullahi Jamiu h, Abraham Osinuga i, Christian Inya Oko j, Sodiq Fakorede k, Mustapha Mangdow k, Oloyede Babatunde l, Zainab Olapade m, Awolola Gbonjubola Victoria n, Abosede Salami o, Idowu A. Usman p, Victor Ifechukwude Agboli p, Ridwan Olamilekan Adesola q: The role of machine learning in discovering biomarkers and predicting treatment strategies for neurodegenerative diseases: A narrative review. NeuroMarkers 2025;2.

[390] Angelucci F, Ai AR, Piendel L, Cerman J, Hort J. Integrating AI in fighting advancing Alzheimer: diagnosis, prevention, treatment, monitoring, mechanisms, and clinical trials. Curr Opin Struct Biol. 2024;87:102857. 38838385 10.1016/j.sbi.2024.102857

[391] Cecot J, Zarzecki K, Mandryk M. Potential Benefits of Using Artificial Intelligence to Diagnose Alzheimer’s Disease. J Clin Neurol. 2024;20:548–9. 39227341 10.3988/jcn.2024.0288 PMC11372216

[392] Friedman BA, Srinivasan K, Ayalon G, Meilandt WJ, Lin H, Huntley MA, Cao Y, Lee SH, Haddick PCG, Ngu H, et al. Diverse Brain Myeloid Expression Profiles Reveal Distinct Microglial Activation States and Aspects of Alzheimer’s Disease Not Evident in Mouse Models. Cell Rep. 2018;22:832–47. 29346778 10.1016/j.celrep.2017.12.066

[393] Gosselin D, Skola D, Coufal NG, Holtman IR, Schlachetzki JCM, Sajti E, Jaeger BN, O’Connor C, Fitzpatrick C, Pasillas MP, et al: An environment-dependent transcriptional network specifies human microglia identity. Science 2017;356:eaal3222. 10.1126/science.aal3222 PMC5858585 28546318

[394] Geirsdottir L, David E, Keren-Shaul H, Weiner A, Bohlen SC, Neuber J, Balic A, Giladi A, Sheban F, Dutertre CA, et al. Cross-Species Single-Cell Analysis Reveals Divergence of the Primate Microglia Program. Cell. 2019;179(1609–1622): e1616. 10.1016/j.cell.2019.11.010 31835035

[395] Speicher AM, Wiendl H, Meuth SG, Pawlowski M. Generating microglia from human pluripotent stem cells: novel in vitro models for the study of neurodegeneration. Mol Neurodegener. 2019;14:46. 31856864 10.1186/s13024-019-0347-z PMC6921408

[396] Penney J, Ralvenius WT, Tsai LH. Modeling Alzheimer’s disease with iPSC-derived brain cells. Mol Psychiatry. 2020;25:148–67. 31391546 10.1038/s41380-019-0468-3 PMC6906186

[397] Zhang W, Jiang J, Xu Z, Yan H, Tang B, Liu C, Chen C, Meng Q. Microglia-containing human brain organoids for the study of brain development and pathology. Mol Psychiatry. 2023;28:96–107. 36474001 10.1038/s41380-022-01892-1 PMC9734443

[398] Popova G, Soliman SS, Kim CN, Keefe MG, Hennick KM, Jain S, Li T, Tejera D, Shin D, Chhun BB, et al. Human microglia states are conserved across experimental models and regulate neural stem cell responses in chimeric organoids. Cell Stem Cell. 2021;28(2153–2166): e2156. 10.1016/j.stem.2021.08.015 PMC8642295 34536354

[399] Keren-Shaul H, Spinrad A, Weiner A, Matcovitch-Natan O, Dvir-Szternfeld R, Ulland TK, David E, Baruch K, Lara-Astaiso D, Toth B, et al. A Unique Microglia Type Associated with Restricting Development of Alzheimer’s Disease. Cell. 2017;169(1276–1290): e1217. 10.1016/j.cell.2017.05.018 28602351

[400] Gordon BA, Blazey TM, Su Y, Hari-Raj A, Dincer A, Flores S, Christensen J, McDade E, Wang G, Xiong C, et al. Spatial patterns of neuroimaging biomarker change in individuals from families with autosomal dominant Alzheimer’s disease: a longitudinal study. Lancet Neurol. 2018;17:241–50. 29397305 10.1016/S1474-4422(18)30028-0 PMC5816717

[401] Xie Z, Situ Y, Deng L, Liang M, Ding H, Guo Z, Xu Q, Liang Z, Shao Z. Identification of therapeutic targets for Alzheimer’s Disease Treatment using bioinformatics and machine learning. Sci Rep. 2025;15:3888. 39890844 10.1038/s41598-025-88134-w PMC11785788

[402] Liang P, Wang Y, Liu J, Huang H, Li Y, Kang J, Li G, Wu H. Identification and Exploration of Immunity-Related Genes and Natural Products for Alzheimer’s Disease Based on Bioinformatics, Molecular Docking, and Molecular Dynamics. Immun Inflamm Dis. 2025;13: e70166. 40192032 10.1002/iid3.70166 PMC11973734

[403] Chudzik A, Sledzianowski A, Przybyszewski AW: Machine Learning and Digital Biomarkers Can Detect Early Stages of Neurodegenerative Diseases. Sensors (Basel) 2024;24:1572. 10.3390/s24051572 PMC10934426 38475108

[404] Menendez-Gonzalez M. Implementing a tridimensional diagnostic framework for personalized medicine in neurodegenerative diseases. Alzheimers Dement. 2025;21: e14591. 39976261 10.1002/alz.14591 PMC11840702

[405] Jellinger KA. Recent update on the heterogeneity of the Alzheimer’s disease spectrum. J Neural Transm (Vienna). 2022;129:1–24. 34919190 10.1007/s00702-021-02449-2

[406] Palmqvist S, Tideman P, Cullen N, Zetterberg H, Blennow K. Alzheimer’s Disease Neuroimaging I, Dage JL, Stomrud E, Janelidze S, Mattsson-Carlgren N, Hansson O: Prediction of future Alzheimer’s disease dementia using plasma phospho-tau combined with other accessible measures. Nat Med. 2021;27:1034–42. 34031605 10.1038/s41591-021-01348-z

[407] Isonaka R, Sullivan P, Holmes C, Goldstein DS. A pathophysiological biomarker combination separates Lewy body from non-Lewy body neurogenic orthostatic hypotension ​. Clin Auton Res. 2024;34:329–39. 38844644 10.1007/s10286-024-01035-2

[408] Goldstein DS, Holmes C, Sullivan P, Lopez G, Gelsomino J, Moore S, Isonaka R, Wu T, Sharabi Y: Cardiac noradrenergic deficiency revealed by 18F-dopamine positron emission tomography identifies preclinical central Lewy body diseases. J Clin Invest 2024;134:e172460. 10.1172/JCI172460 PMC10760969 37883190

[409] Kwon HS, Koh SH. Neuroinflammation in neurodegenerative disorders: the roles of microglia and astrocytes. Transl Neurodegener. 2020;9:42. 33239064 10.1186/s40035-020-00221-2 PMC7689983

[410] Leng F, Edison P. Neuroinflammation and microglial activation in Alzheimer disease: where do we go from here? Nat Rev Neurol. 2021;17:157–72. 33318676 10.1038/s41582-020-00435-y

[411] Morotti A, Pilotto A, Zanola D, Galli A, Caratozzolo S, Gasparotti R, Padovani A. Cerebral Amyloid Angiopathy in Alzheimer Disease: A Comparison Between Different Versions of the Boston Criteria. Neurology. 2025;104: e210248. 39836667 10.1212/WNL.0000000000210248

[412] Franzmeier N, Roemer-Cassiano SN, Bernhardt AM, Dehsarvi A, Dewenter A, Steward A, Biel D, Frontzkowski L, Zhu Z, Gnorich J, et al. Alpha synuclein co-pathology is associated with accelerated amyloid-driven tau accumulation in Alzheimer’s disease. Mol Neurodegener. 2025;20:31. 40098057 10.1186/s13024-025-00822-3 PMC11916967

[413] Sheena M. Posey Norris R, Eva Childers, Rapporteur, and Sarah Carter: *Multimodal Biomarkers for Central Nervous System Disorders* National Academies Forum on Neuroscience and Nervous System Disorders; 2023. 37651556

[414] Mehdipour Ghazi M, Selnes P, Timon-Reina S, Tecelao S, Ingala S, Bjornerud A, Kirsebom BE, Fladby T, Nielsen M. Comparative analysis of multimodal biomarkers for amyloid-beta positivity detection in Alzheimer’s disease cohorts. Front Aging Neurosci. 2024;16:1345417. 38469163 10.3389/fnagi.2024.1345417 PMC10925621

[415] Ravichandran S, Snyder PJ, Alber J, Murchison CF, Chaby LE, Jeromin A, Arthur E. Association and multimodal model of retinal and blood-based biomarkers for detection of preclinical Alzheimer’s disease. Alzheimers Res Ther. 2025;17:19. 39794837 10.1186/s13195-024-01668-5 PMC11720872

[416] Companion Diagnostics Explained: Their Critical Role in Cancer Care and Our Latest Approvals (https://www.foundationmedicine.com/blog/companion-diagnostics-explained-their-critical-role-cancer-care-and-our-latest-approvals).

[417] Robinson JL, Xie SX, Baer DR, Suh E, Van Deerlin VM, Loh NJ, Irwin DJ, McMillan CT, Wolk DA, Chen-Plotkin A, et al. Pathological combinations in neurodegenerative disease are heterogeneous and disease-associated. Brain. 2023;146:2557–69. 36864661 10.1093/brain/awad059 PMC10232273

[418] Wen J, Yang Z, Nasrallah IM, Cui Y, Erus G, Srinivasan D, Abdulkadir A, Mamourian E, Hwang G, Singh A, et al. Genetic and clinical correlates of two neuroanatomical AI dimensions in the Alzheimer’s disease continuum. Transl Psychiatry. 2024;14:420. 39368996 10.1038/s41398-024-03121-5 PMC11455841

