# Supplementary material for: Biomarkers and therapeutic strategies targeting microglia in neurodegenerative diseases: current status and future directions
Source: Mol Neurodegener. 2025 Jul 10;20:82. doi: 10.1186/s13024-025-00867-4 (PMC12247225; doi:10.1186/s13024-025-00867-4)
Supplement: Supplementary file 1 — Additional file 1. [file 13024_2025_867_MOESM1_ESM.docx]

**Supplementary Table 1**. Summary of Molecular Targets and Microglia-Targeting Agents in Preclinical Studies (Refer to Fig. 2 for Proposed List).

| **Classification by Targeted Microglial Function** | **Molecular Target** | **Candidate**  **Agents** | **Mode of Action** | **Disease Models** | **Summary of Main Outcome** | **Reference** |
| --- | --- | --- | --- | --- | --- | --- |
| A. Targeting for microglial phagocytosis | CD22 | CD22 inhibitor | Inhibit | AD | CD22 inhibition increased phagocytic capacity and revealed an influence on BMP and TGFβ-associated gene networks, restoring age-related impairments of microglia surveillance capacity. | [1] |
|  | CD33 | CD33 knockout,  AAV-mediated miRCD33 Inhibitor, Lintuzumab, AL003 | Inhibit | AD | Blocked CD33 expression promoted Aβ phagocytosis and clearance; decreased neuroinflammatory (by CD33 knockout, AAV-mediated miRCD33 Inhibitor, Lintuzumab, AL003). | [2-4] |
|  | COX2-S565 acetylation | 15-R-Lipoxin A4 | Activate | AD | Elevation of SphK1 increased SPMs secretion, especially 15-R-Lipoxin A4, by promoting acetylation of S565 of COX2 using acetyl-CoA, improving AD-like pathology in APP/PS1 mice. | [5] |
|  | IDE | genetic modulation | Activate | AD | IDE promotes the degradation of amyloid-β in the brain. Without an IDE, microglia are delayed in their response to stimuli, including the induction of phagocytosis of myelin and the degradation of Aβ oligomers, which provokes transitory effects that alter microglial phenotypes. IDE as a modulator of microglial phenotypes improves cognitive impairment associated with AD and diabetes. | [6] |
|  | LILRB4 | anti-human LILRB4 mAb | Inhibit | AD | Systemic treatment of these mice with an anti-human LILRB4 mAb reduced Aβ load and enhanced microglia activation and phagocytosis. | [7] |
|  | miR-181c-5p | miR-181c-5p inhibitor | Inhibit | AD | Downregulated miR-181c-5p impaired the phagocytosis and degradation of Aβ by BV2 cells. | [8, 9] |
|  | miR-214-3p | miR-214-3p inhibitor | Inhibit | ALS  PD | Plasma miRNA-214-3p is an accessible biomarker for predicting the future progression speed related to phagocytic dysfunction in ALS patients./miR–214–3p inhibits autophagy and promotes dopaminergic neuron apoptosis. | [10, 11] |
|  | NCKAP1 | NCKAP1 overexpression | Activate | ALS | NCKAP1 overexpression rescued impaired phagocytosis via regulated actin polymerization in rapidly progressive ALS patients derived iMGs. | [12] |
|  | PGRN | PGRN overexpression | Activate | FTD | Lentivirus-mediated PGRN overexpression lowered plaque load in AD mice with aggressive amyloid plaque pathology and prevented spatial memory deficits and hippocampal neuronal loss in AD mice. | [13] |
|  | Piezo1 | Yoda1 | Activate | AD | Activation of PIEZO1 with a selective agonist, Yoda1, improved microglial phagocytosis, resulting in Aβ clearance in human iMGL and mouse models of AD. | [14, 15] |
|  | P2Y6R | P2Y6R antagonist | Inhibit | AD  PD | Inhibition of P2Y6R reduces microglia recruitment, activation, and phagocytosis. | [16-19] |
|  | Pyk2 | Pyk2 inhibitor | Inhibit | AD | Pyk2 inhibition enhances phagocytosis in proteolytic activity and modulates microglial functions, potentially reducing neuroinflammation. | [20] |
|  | SYK | SYK deletion | Activate | AD | Microglia-specific deletion of SYK in microglia leads to exacerbated Aβ deposition, aggravated neuropathology, and cognitive defects in the 5xFAD mouse; CLEC7A-induced activation of SYK in 5xFAD mice promotes improved clearance of Aβ. | [21] |
|  | TAM receptors | Tyro3, Axl, MerTK activators | Activate | AD | TAM system is required for microglial recognition of, response to, and phagocytosis of Aβ plaques; TAM-mediated microglial phagocytosis of Aβ material does not inhibit but promotes the formation of dense-core plaques. | [22] |
| B. Preclinical TREM2-targeting agents | TREM2 | 4D9 | Activate | AD | 4D9 reduced amyloidogenesis, enhanced microglial TREM2 expression, and reduced a homeostatic marker, suggesting a protective function by driving microglia toward a disease-associated state. | [23] |
|  | TREM2 | Ab18 TVD-Ig/αTfR | Activate | AD | Ab18 TVD-Ig/αTfR showed a considerable reduction of amyloid burden with increased microglia migration to and phagocytosis of amyloid plaques, improved synaptic and neuronal marker intensity, improved cognitive functions, reduced endogenous tau hyperphosphorylation, and decreased phosphorylated neurofilament H. | [24] |
|  | TREM2 | ASOs-Trem2 | Inhibit | AD | ASO-mediated Trem2 knockdown triggers increased microglial phagocytosis, slowing amyloid deposition in mice. | [25] |
|  | TREM2 | Intermittent hypoxia training (IHT) | Activate | AD | Intermittent hypoxia training enhances Aβ endocytosis by plaque-associated microglia via VPS35-dependent TREM2 recycling. | [26] |
|  | TREM2 | Microglia-targeted gene delivery systems (PHSA@PF/pTREM2) | Activate | AD | PHSA@PF/pTREM2 upregulates the TREM2 level and regulates microglial polarization toward the M2-phenotype for remodeling the inﬂammatory microenvironment and enhanced Aβ clearance, leading to an improvement of cognitive performance in APP/PS1 mice. | [27] |
|  | human APOE  and TREM2 | RNase-H active ASOs | Inhibit | AD | APOE- and TREM2-targeting ASOs are pharmacologically active and can modify the phenotype of human microglia in vivo using a xenotransplantation mouse model grafted with human microglia. | [28] |
| C. Targeting the microglial phenotype switch | HCAR2 | Niaspan | Activate | AD | Activation of HCAR2 with an FDA-approved formulation of niacin (Niaspan) in 5xFAD mice leads to reduced plaque burden and neuronal dystrophy, attenuation of neuronal loss, and rescue of working memory deficits. | [29] |
|  | miR-132 | miR-132 mimic | Activate | AD | miR-132 shifts human iPSC-derived microglia from a disease-associated toward a homeostatic state. | [30] |
|  | miR-155 | miR-155 inhibitor  or deletion | Inhibit | AD  ALS | Microglial deletion of miR-155 induces a pre-MGnD activation state via interferon-γ (IFN-γ) signaling, and blocking IFN-γ signaling attenuates MGnD induction and microglial phagocytosis.  Genetic ablation of miR-155 reverses impaired microglia phagocytic function in SOD1 mice, and the administration of anti-miR-155 inhibitor delays disease onset and extends survival in SOD1 mice. | [31, 32] |
|  | PPAR-γ | Rosiglitazone | Activate | PD | Rosiglitazone is an FDA-approved drug for treating type 2 diabetes mellitus by improving insulin action through activation of the nuclear peroxisome proliferator-activated receptor-gamma (PPAR-γ), modulating microglia polarization, and boosting M2 over the pro-inflammatory phenotype. | [33] |
|  | REV-ERBs | REV-ERB inhibitors or genetic knockdown | Inhibit | AD | Inhibition of REV-ERBs promoted microglia polarization toward a phagocytic phenotype, enhancing microglia-mediated Aβ degradation and limiting amyloid plaque deposition in AD. | [34] |
| D. Targeting the microglial metabolic pathway | HK2 | HK2 partial antagonist | Inhibit | AD | In the AD brain, microglial HK2 is upregulated and promotes inflammation through metabolic and non-metabolic functions. Partial antagonism of HK2 reduces pathology by modulating NF-κB signaling via IKBα, while complete loss worsens inflammation due to mitochondrial dysfunction. | [35] |
|  | H4K12la | PKM2 inhibitor | Inhibit | AD | In AD, H4K12la is elevated in microglia near Aβ plaques, promoting glycolytic gene expression and driving a glycolysis/H4K12la/PKM2 feedback loop that worsens microglial dysfunction. Inhibiting PKM2 or deleting it specifically in microglia reduces activation and improves cognitive function in AD mice. | [36] |
| E. Targeting for microglia-mediated neuroinflammation | AGE-RAGE pathway | Dl-3-n-butylphthalide (NBP) | Inhibit | AD | NBP was approved by the FDA of China in 2002 for clinical use as an anti–cerebral ischemia agent, alleviating cognitive impairment by augmenting the number of activated microglia around Aβ plaques and ameliorating AGE-RAGE-mediated neuroinflammation. | [37] |
|  | AKAP8L | AKAP8L knockdown | Inhibit | AD | AKAP8L knockdown and rapamycin treatment enhanced cognitive impairment via autophagy inhibition and neuroinflammation triggering in the diabetes-associated cognitive impairment (DACI) model. | [38] |
|  | AMPKα1 | DW14006 | Activate | AD | Direct AMPKα1 activator DW14006 reduced amyloid plaque deposition by promoting microglial phagocytosis and ameliorated innate immune response by polarizing microglia to an anti-inflammatory phenotype. | [39] |
|  | ASC | ACI-6635 | Inhibit | AD | Anti-ASC antibody, ACI-6635, has been shown to reduce ASC-induced Aß42 aggregation in vitro, while similar administration in APP/PS1 mice can reduce plaque size and the amount of plaque-adjacent ASCs. | [40] |
|  | BACE-1 | BACE-1 deletion | Inhibit | AD | Targeted deletion of Bace-1 in microglia enhances amyloid clearance and improves cognitive performance. | [41] |
|  | Caspase-1 | VX-740 and VX-765 | Inhibit | AD | VX-740 and VX-765 selectively inhibit caspase-1, which can block the secretion of IL-1β and IL-18, thereby reducing inflammatory responses. Inhibition of caspase-1 has effectively ameliorated cognitive impairment in AD mice. | [42] |
|  | CB2R | CB2R agonist | Activate | AD  PD  ALS | CB2 receptor activation reduces protein aggregation-based pathology and attenuates inflammation and dementia-related symptoms. | [43] |
|  | Combination with galectin-1 and SOD1 | AAV9.SOD1.shRNA.Gal1 | Inhibit | ALS | AAV9.SOD1.shRNA.Gal-1 treatment decreases inflammatory markers, rescues motor neuron death, and extends the survival of SOD1G93A mice. | [44] |
|  | Complement C3 | Complement C3 inhibitor | Inhibit | AD | C3 or downstream complement activation fragments may play an essential role in Aβ plaque pathology, glial responses to plaques, and neuronal dysfunction in the brains of APP/PS1 mice. | [45] |
|  | COX2 acetylation | N-AS-triggered SPMs | Activate | AD | Treatment with N-AS increases acetyl-S565 COX2 and N-AS-triggered SPMs in AD mice microglia, leading to neuroinflammation resolution, microglial phagocytosis, and improved memory. | [46] |
|  | IGFBPL1 | IGFBPL1 deficiency or selective deletion of IGF1R | Activate | AD | IGFBPL1 is a master driver of microglia homeostasis and resolution of neuroinflammation in glaucoma and brain tauopathy. | [47] |
|  | miR-146a | miR-146a mimic | Activate | AD | miR-146a reduced cognitive deficits in learning and memory, attenuated neuroinflammation, reduced Aβ levels, ameliorated plaque-associated neuritic pathology, and prevented neuronal loss in APP/PS1 transgenic mice.; switched the microglial phenotype, reduced pro-inflammatory cytokines, and enhanced phagocytic function. | [48] |
|  | miR-218-5p-Ddx41 axis | miR-218-5p inhibitor | Inhibit | PD | miR-218-5p suppresses microglia-mediated neuroinflammation and preserves DA neurons via Ddx41/IFN-I. | [49] |
|  | NF-κB | (E)-4-(2-nitrovinyl) benzoic acid (BANA) | Inhibit | ALS | (E)-4-(2-nitrovinyl) benzoic acid (BANA) reduces reactive microglia and prolongs survival by inhibiting NF-κB activation. | [50] |
|  | NLRP3 | N, N′-Diacetyl- p-phenylenediamine (DAPPD) | Inhibit | AD | DAPPD reduced Aβ burden and improved cognitive function; it suppressed the expression of NLRP3 inflammasome-associated proteins through its impact on the NF-κB pathway. | [51] |
|  | NLRP3 | Diphenyl diselenide (DPDS) | Inhibit | ALS | DPDS improved motor deficit, prolonged survival, and reduced motor neuronal loss by suppressing microglia activation by inhibiting NLRP3 inflammasome activation and IΚb/NF-κB pathway. | [52] |
|  | NLRP3 | Dihydromyricetin (DHM) | Inhibit | AD | DHM prevents the progression of AD-like pathology through the inhibition of NLRP3 inflammasome-based microglia-mediated neuroinflammation. | [53] |
|  | NLRP3 | SCAP deficiency | Inhibit | AD | Microglial SCAP deficiency protects against diabetes-associated cognitive impairment through inhibiting NLRP3 inflammasome-mediated neuroinflammation. | [54] |
|  | NLRP3 | JC-124 | Inhibit | AD | JC-124, as a specific NLRP3 inflammasome inhibitor, blocks NLRP3 formation and caspase-1 activation. | [55] |
|  | P2RX7 | GSK1482160 | Inhibit | AD | The oral administration of the P2RX7-specific inhibitor GSK1482160 mitigates disease phenotypes in P301S mice, likely by suppressing the release of microglial exosomes, decreasing tau accumulation in the brain, and improving working and contextual memory. | [56] |
|  | PKCδ | PKCδ inhibitor | Inhibit | AD | PKCδ levels were increased dramatically in the CSF of AD patients; inhibition of PKCδ attenuated Aβ-induced microglial responses and improved cognitive function in an AD mouse model. | [57] |
|  | RIPK1 | RIPK1 inhibitor | Inhibit | AD | Inhibition of RIPK1 reduced neuroinflammation, decreased the cerebral Aβ load, and improved the behavioral deficits by enhancing the microglial degradation of Aβ. | [58] |
|  | SPMs | RvD1 and LXA4 | Activate | AD  PD | SPMs hold great promise for neuroprotection in AD by altering gene expression of pro-inflammatory genes, modulating macrophage function, serving as a biomarker for AD status, and promoting the resolution of neuroinflammation. In PD, data from in vitro, in vivo, and observational studies suggest SPM can cross the blood-brain barrier, inhibit microglial activation, and decrease induced markers of inflammation, possibly due to their ability to downregulate NFκB signaling pathways. | [59-62] |
|  | STING | cGAMP | Activate | AD | cGAMP decreased Aβ burden and neuron loss and improved cognitive impairment via elevating TREM2 expression in AD mouse models. | [63] |

Abbreviations: AAV; Adeno-associated Virus, acetyl-S565 COX2; acetylation of serine 565 residues of cyclooxygenase-2, AD; Alzheimer's disease, AGE; advanced glycation end products, AKAP8L; A-kinase anchor protein 8-like, ALS; Amyotrophic lateral sclerosis, AMPKα1; AMP-activated protein kinase α1, APOE; Apoprotein E, ASC; Apoptosis-associated speck-like protein containing a caspase recruitment domain, ASO; Antisense oligonucleotide, Aβ; Amyloid β, BACE1;  Beta-site amyloid precursor protein cleaving enzyme1, BANA; (E)-4-(2-nitrovinyl) benzoic acid, C3; Complement component 3, CB2R; Cannabinoid receptor type 2, cGAMP; Cyclic guanosine monophosphate–adenosine monophosphate, DAPPD; N, N′-Diacetyl- p-phenylenediamine, DHM; Dihydromyricetin, DLT; Antiallergic drug desloratadine, DM; Diabetes mellitus, DPDS; Diphenyl diselenide, FTD; Frontotemporal dementia, Gal1; Galectin-1, H4K12la; Histone H4 lysine 12 lactylation, HCAR2; Hydroxycarboxylic acid receptor 2, HK2; Hexokinase 2, H4K12la; Histone H4 lysine 12 lactylation, IDE; Insulin-degrading enzyme, IGFBPL1; Insulin-like growth factor binding protein-like 1, IHT; Intermittent hypoxia training, LXA4; Lipoxin A4, LILRB4; Leukocyte Ig-like receptor B4, mAb; Monoclonal antibody, miRNA (miR); microRNA, NCKAP1; NCK Associated Protein 1, NaR; Sodium rutin, NBP; Dl-3-n-butylphthalide, N-AS; N-acetyl sphongosine, NLRP3; NLR family pyrin domain containing 3, P2RX7; Purinergic Receptor P2X7, P2Y6R; P2Y6 receptor, PD; Parkinson’s disease, PGRN; Progranulin, Piezo 1; Piezo 1 Ion Channel Protein, PKCδ; Protein kinase C delta, PKM2; Pyruvate kinase M2, PPAR; Peroxisome proliferator-activated receptors, Pyk2; Protein tyrosine kinase 2-beta, RAGE; receptor for AGE, RIPK1; Receptor-interacting serine/threonine-protein kinase 1, RvD1; Resolvin D1**,** SCAP; Sterol Regulatory Element-Binding Protein (SREBP) cleavage-activating protein, shRNA; Short hairpin RNA, SOD1; Superoxide dismutase type 1, SPMs; Specialized pro-resolving mediators, TAM receptors; Tyro3, Axl and MerTK, TDP-43; TREM2; Triggering Receptor Expressed on Myeloid Cells 2.

**References**

1. Aires V, Coulon-Bainier C, Pavlovic A, Ebeling M, Schmucki R, Schweitzer C, Kueng E, Gutbier S, Harde E: **CD22 Blockage Restores Age-Related Impairments of Microglia Surveillance Capacity.** *Front Immunol* 2021, **12:**684430.

2. Griciuc A, Federico AN, Natasan J, Forte AM, McGinty D, Nguyen H, Volak A, LeRoy S, Gandhi S, Lerner EP, et al: **Gene therapy for Alzheimer's disease targeting CD33 reduces amyloid beta accumulation and neuroinflammation.** *Hum Mol Genet* 2020, **29:**2920-2935.

3. Miles LA, Hermans SJ, Crespi GAN, Gooi JH, Doughty L, Nero TL, Markulic J, Ebneth A, Wroblowski B, Oehlrich D, et al: **Small Molecule Binding to Alzheimer Risk Factor CD33 Promotes Abeta Phagocytosis.** *iScience* 2019, **19:**110-118.

4. Malik M, Chiles J, 3rd, Xi HS, Medway C, Simpson J, Potluri S, Howard D, Liang Y, Paumi CM, Mukherjee S, et al: **Genetics of CD33 in Alzheimer's disease and acute myeloid leukemia.** *Hum Mol Genet* 2015, **24:**3557-3570.

5. Lee JY, Han SH, Park MH, Baek B, Song IS, Choi MK, Takuwa Y, Ryu H, Kim SH, He X, et al: **Neuronal SphK1 acetylates COX2 and contributes to pathogenesis in a model of Alzheimer's Disease.** *Nat Commun* 2018, **9:**1479.

6. Corraliza-Gomez M, Bermejo T, Lilue J, Rodriguez-Iglesias N, Valero J, Cozar-Castellano I, Arranz E, Sanchez D, Ganfornina MD: **Insulin-degrading enzyme (IDE) as a modulator of microglial phenotypes in the context of Alzheimer's disease and brain aging.** *J Neuroinflammation* 2023, **20:**233.

7. Hou J, Chen Y, Cai Z, Heo GS, Yuede CM, Wang Z, Lin K, Saadi F, Trsan T, Nguyen AT, et al: **Antibody-mediated targeting of human microglial leukocyte Ig-like receptor B4 attenuates amyloid pathology in a mouse model.** *Sci Transl Med* 2024, **16:**eadj9052.

8. Li R, Yao S, Wei F, Chen M, Zhong Y, Zou C, Chen L, Wei L, Yang C, Zhang X, Liu Y: **Downregulation of miR-181c-5p in Alzheimer's disease weakens the response of microglia to Abeta phagocytosis.** *Sci Rep* 2024, **14:**11487.

9. Yan Y, Yan H, Teng Y, Wang Q, Yang P, Zhang L, Cheng H, Fu S: **Long non-coding RNA 00507/miRNA-181c-5p/TTBK1/MAPT axis regulates tau hyperphosphorylation in Alzheimer's disease.** *J Gene Med* 2020, **22:**e3268.

10. Noh M-Y, Kwon M-S, Oh K-W, Nahm M, Park J, Kim Y-E, Jin HK, Bae J-s, Ki C-S, Kim SH: **Plasma miRNA-214 is a predictive candidate biomarker of progression speed in patients with ALS.** *bioRxiv* 2022**:**2022.2005.2004.490596.

11. Dong H, Yan J, Huang P, Wang X, Zhang R, Zhang C, Wang W, Qian W, Zhou J, Zhao Y, et al: **miR-214-3p promotes the pathogenesis of Parkinson's disease by inhibiting autophagy.** *Biomed Pharmacother* 2024, **171:**116123.

12. Noh MY, Kwon MS, Oh KW, Nahm M, Park J, Kim YE, Ki CS, Jin HK, Bae JS, Kim SH: **Role of NCKAP1 in the Defective Phagocytic Function of Microglia-Like Cells Derived from Rapidly Progressing Sporadic ALS.** *Mol Neurobiol* 2023, **60:**4761-4777.

13. Ek M, Nilvebrant J, Nygren PA, Stahl S, Lindberg H, Lofblom J: **An anti-sortilin affibody-peptide fusion inhibits sortilin-mediated progranulin degradation.** *Front Immunol* 2024, **15:**1437886.

14. Ikiz ED, Hascup ER, Bae C, Hascup KN: **Microglial Piezo1 mechanosensitive channel as a therapeutic target in Alzheimer's disease.** *Front Cell Neurosci* 2024, **18:**1423410.

15. Jantti H, Sitnikova V, Ishchenko Y, Shakirzyanova A, Giudice L, Ugidos IF, Gomez-Budia M, Korvenlaita N, Ohtonen S, Belaya I, et al: **Microglial amyloid beta clearance is driven by PIEZO1 channels.** *J Neuroinflammation* 2022, **19:**147.

16. Dundee JM, Brown GC: **The microglial P2Y(6) receptor as a therapeutic target for neurodegenerative diseases.** *Transl Neurodegener* 2024, **13:**47.

17. Umpierre AD, Li B, Ayasoufi K, Simon WL, Zhao S, Xie M, Thyen G, Hur B, Zheng J, Liang Y, et al: **Microglial P2Y(6) calcium signaling promotes phagocytosis and shapes neuroimmune responses in epileptogenesis.** *Neuron* 2024, **112:**1959-1977 e1910.

18. Yang X, Lou Y, Liu G, Wang X, Qian Y, Ding J, Chen S, Xiao Q: **Microglia P2Y6 receptor is related to Parkinson's disease through neuroinflammatory process.** *J Neuroinflammation* 2017, **14:**38.

19. Timmerman R, Zuiderwijk-Sick EA, Bajramovic JJ: **P2Y6 receptor-mediated signaling amplifies TLR-induced pro-inflammatory responses in microglia.** *Front Immunol* 2022, **13:**967951.

20. Lee JW, Mizuno K, Watanabe H, Lee IH, Tsumita T, Hida K, Yawaka Y, Kitagawa Y, Hasebe A, Iimura T, Kong SW: **Enhanced phagocytosis associated with multinucleated microglia via Pyk2 inhibition in an acute beta-amyloid infusion model.** *J Neuroinflammation* 2024, **21:**196.

21. Ennerfelt H, Frost EL, Shapiro DA, Holliday C, Zengeler KE, Voithofer G, Bolte AC, Lammert CR, Kulas JA, Ulland TK, Lukens JR: **SYK coordinates neuroprotective microglial responses in neurodegenerative disease.** *Cell* 2022, **185:**4135-4152 e4122.

22. Huang Y, Happonen KE, Burrola PG, O'Connor C, Hah N, Huang L, Nimmerjahn A, Lemke G: **Microglia use TAM receptors to detect and engulf amyloid beta plaques.** *Nat Immunol* 2021, **22:**586-594.

23. Schlepckow K, Monroe KM, Kleinberger G, Cantuti-Castelvetri L, Parhizkar S, Xia D, Willem M, Werner G, Pettkus N, Brunner B, et al: **Enhancing protective microglial activities with a dual function TREM2 antibody to the stalk region.** *EMBO Mol Med* 2020, **12:**e11227.

24. Zhao P, Xu Y, Jiang L, Fan X, Li L, Li X, Arase H, Zhao Y, Cao W, Zheng H, et al: **A tetravalent TREM2 agonistic antibody reduced amyloid pathology in a mouse model of Alzheimer's disease.** *Sci Transl Med* 2022, **14:**eabq0095.

25. Schoch KM, Ezerskiy LA, Morhaus MM, Bannon RN, Sauerbeck AD, Shabsovich M, Jafar-Nejad P, Rigo F, Miller TM: **Acute Trem2 reduction triggers increased microglial phagocytosis, slowing amyloid deposition in mice.** *Proc Natl Acad Sci U S A* 2021, **118**.

26. Wang X, Xie Y, Fan X, Wu X, Wang D, Zhu L: **Intermittent hypoxia training enhances Abeta endocytosis by plaque associated microglia via VPS35-dependent TREM2 recycling in murine Alzheimer's disease.** *Alzheimers Res Ther* 2024, **16:**121.

27. Wang P, Yang P, Qian K, Li Y, Xu S, Meng R, Guo Q, Cheng Y, Cao J, Xu M, et al: **Precise gene delivery systems with detachable albumin shell remodeling dysfunctional microglia by TREM2 for treatment of Alzheimer's disease.** *Biomaterials* 2022, **281:**121360.

28. Vandermeulen L, Geric I, Fumagalli L, Kreir M, Lu A, Nonneman A, Premereur J, Wolfs L, Policarpo R, Fattorelli N, et al: **Regulation of human microglial gene expression and function via RNAase-H active antisense oligonucleotides in vivo in Alzheimer's disease.** *Mol Neurodegener* 2024, **19:**37.

29. Moutinho M, Puntambekar SS, Tsai AP, Coronel I, Lin PB, Casali BT, Martinez P, Oblak AL, Lasagna-Reeves CA, Lamb BT, Landreth GE: **The niacin receptor HCAR2 modulates microglial response and limits disease progression in a mouse model of Alzheimer's disease.** *Sci Transl Med* 2022, **14:**eabl7634.

30. Walgrave H, Penning A, Tosoni G, Snoeck S, Davie K, Davis E, Wolfs L, Sierksma A, Mars M, Bu T, et al: **microRNA-132 regulates gene expression programs involved in microglial homeostasis.** *iScience* 2023, **26:**106829.

31. Yin Z, Herron S, Silveira S, Kleemann K, Gauthier C, Mallah D, Cheng Y, Margeta MA, Pitts KM, Barry JL, et al: **Identification of a protective microglial state mediated by miR-155 and interferon-gamma signaling in a mouse model of Alzheimer's disease.** *Nat Neurosci* 2023, **26:**1196-1207.

32. Butovsky O, Jedrychowski MP, Cialic R, Krasemann S, Murugaiyan G, Fanek Z, Greco DJ, Wu PM, Doykan CE, Kiner O, et al: **Targeting miR-155 restores abnormal microglia and attenuates disease in SOD1 mice.** *Ann Neurol* 2015, **77:**75-99.

33. Pisanu A, Lecca D, Mulas G, Wardas J, Simbula G, Spiga S, Carta AR: **Dynamic changes in pro- and anti-inflammatory cytokines in microglia after PPAR-gamma agonist neuroprotective treatment in the MPTPp mouse model of progressive Parkinson's disease.** *Neurobiol Dis* 2014, **71:**280-291.

34. Lee J, Kim DE, Griffin P, Sheehan PW, Kim DH, Musiek ES, Yoon SY: **Inhibition of REV-ERBs stimulates microglial amyloid-beta clearance and reduces amyloid plaque deposition in the 5XFAD mouse model of Alzheimer's disease.** *Aging Cell* 2020, **19:**e13078.

35. Codocedo JF, Mera-Reina C, Bor-Chian Lin P, Fallen PB, Puntambekar SS, Casali BT, Jury-Garfe N, Martinez P, Lasagna-Reeves CA, Landreth GE: **Therapeutic targeting of immunometabolism reveals a critical reliance on hexokinase 2 dosage for microglial activation and Alzheimer's progression.** *Cell Rep* 2024, **43:**114488.

36. Pan RY, He L, Zhang J, Liu X, Liao Y, Gao J, Liao Y, Yan Y, Li Q, Zhou X, et al: **Positive feedback regulation of microglial glucose metabolism by histone H4 lysine 12 lactylation in Alzheimer's disease.** *Cell Metab* 2022, **34:**634-648 e636.

37. Lu J, Zhang J, Wang X, Yuan F, Xin B, Li J, Yang Q, Li X, Zhang J, Wang X, et al: **Dl-3-n-butylphthalide promotes microglial phagocytosis and inhibits microglial inflammation via regulating AGE-RAGE pathway in APP/PS1 mice.** *Brain Res Bull* 2024, **212:**110969.

38. Zhang WY, Wei QQ, Zhang T, Wang CS, Chen J, Wang JH, Xie X, Jiang P: **Microglial AKAP8L: a key mediator in diabetes-associated cognitive impairment via autophagy inhibition and neuroinflammation triggering.** *J Neuroinflammation* 2024, **21:**177.

39. Lv J, Wang W, Zhu X, Xu X, Yan Q, Lu J, Shi X, Wang Z, Zhou J, Huang X, et al: **DW14006 as a direct AMPKalpha1 activator improves pathology of AD model mice by regulating microglial phagocytosis and neuroinflammation.** *Brain Behav Immun* 2020, **90:**55-69.

40. Davide Basco P: **TARGETING THE INFLAMMASOME PATHWAY WITH AN ANTI-ASC IMMUNOTHERAPY IN ALZHEIMER’S DISEASE.** In *Book TARGETING THE INFLAMMASOME PATHWAY WITH AN ANTI-ASC IMMUNOTHERAPY IN ALZHEIMER’S DISEASE* (Editor ed.^eds.). City; 2024.

41. Singh N, Das B, Zhou J, Hu X, Yan R: **Targeted BACE-1 inhibition in microglia enhances amyloid clearance and improved cognitive performance.** *Sci Adv* 2022, **8:**eabo3610.

42. Flores J, Fillion ML, LeBlanc AC: **Caspase-1 inhibition improves cognition without significantly altering amyloid and inflammation in aged Alzheimer disease mice.** *Cell Death Dis* 2022, **13:**864.

43. Vuic B, Milos T, Tudor L, Konjevod M, Nikolac Perkovic M, Jazvinscak Jembrek M, Nedic Erjavec G, Svob Strac D: **Cannabinoid CB2 Receptors in Neurodegenerative Proteinopathies: New Insights and Therapeutic Potential.** *Biomedicines* 2022, **10**.

44. Baird MC, Likhite SB, Vetter TA, Caporale JR, Girard HB, Roussel FS, Howard AE, Schwartz MK, Reed AR, Kaleem A, et al: **Combination AAV therapy with galectin-1 and SOD1 downregulation demonstrates superior therapeutic effect in a severe ALS mouse model.** *Mol Ther Methods Clin Dev* 2024, **32:**101312.

45. Shi Q, Chowdhury S, Ma R, Le KX, Hong S, Caldarone BJ, Stevens B, Lemere CA: **Complement C3 deficiency protects against neurodegeneration in aged plaque-rich APP/PS1 mice.** *Sci Transl Med* 2017, **9**.

46. Lee JY, Han SH, Park MH, Song IS, Choi MK, Yu E, Park CM, Kim HJ, Kim SH, Schuchman EH, et al: **N-AS-triggered SPMs are direct regulators of microglia in a model of Alzheimer's disease.** *Nat Commun* 2020, **11:**2358.

47. Pan L, Cho KS, Wei X, Xu F, Lennikov A, Hu G, Tang J, Guo S, Chen J, Kriukov E, et al: **IGFBPL1 is a master driver of microglia homeostasis and resolution of neuroinflammation in glaucoma and brain tauopathy.** *Cell Rep* 2023, **42:**112889.

48. Liang C, Zou T, Zhang M, Fan W, Zhang T, Jiang Y, Cai Y, Chen F, Chen X, Sun Y, et al: **MicroRNA-146a switches microglial phenotypes to resist the pathological processes and cognitive degradation of Alzheimer's disease.** *Theranostics* 2021, **11:**4103-4121.

49. Wang D, Gao H, Qin Q, Li J, Zhao J, Qu Y, Li J, Xiong Y, Min Z, Mao Z, Xue Z: **MicroRNA-218-5p-Ddx41 axis restrains microglia-mediated neuroinflammation through downregulating type I interferon response in a mouse model of Parkinson's disease.** *J Transl Med* 2024, **22:**63.

50. Ibarburu S, Kovacs M, Varela V, Rodriguez-Duarte J, Ingold M, Invernizzi P, Porcal W, Arevalo AP, Perelmuter K, Bollati-Fogolin M, et al: **A Nitroalkene Benzoic Acid Derivative Targets Reactive Microglia and Prolongs Survival in an Inherited Model of ALS via NF-kappaB Inhibition.** *Neurotherapeutics* 2021, **18:**309-325.

51. Park MH, Lee M, Nam G, Kim M, Kang J, Choi BJ, Jeong MS, Park KH, Han WH, Tak E, et al: **N,N'-Diacetyl-p-phenylenediamine restores microglial phagocytosis and improves cognitive defects in Alzheimer's disease transgenic mice.** *Proc Natl Acad Sci U S A* 2019, **116:**23426-23436.

52. Zhang C, Wang H, Liang W, Yang Y, Cong C, Wang Y, Wang S, Wang X, Wang D, Huo D, Feng H: **Diphenyl diselenide protects motor neurons through inhibition of microglia-mediated inflammatory injury in amyotrophic lateral sclerosis.** *Pharmacol Res* 2021, **165:**105457.

53. Feng J, Wang JX, Du YH, Liu Y, Zhang W, Chen JF, Liu YJ, Zheng M, Wang KJ, He GQ: **Dihydromyricetin inhibits microglial activation and neuroinflammation by suppressing NLRP3 inflammasome activation in APP/PS1 transgenic mice.** *CNS Neurosci Ther* 2018, **24:**1207-1218.

54. Zhu W, Zhang H, Niu T, Liu K, Fareeduddin Mohammed Farooqui H, Sun R, Chen X, Yuan Y, Wang S: **Microglial SCAP deficiency protects against diabetes-associated cognitive impairment through inhibiting NLRP3 inflammasome-mediated neuroinflammation.** *Brain Behav Immun* 2024, **119:**154-170.

55. Yin J, Zhao F, Chojnacki JE, Fulp J, Klein WL, Zhang S, Zhu X: **NLRP3 Inflammasome Inhibitor Ameliorates Amyloid Pathology in a Mouse Model of Alzheimer's Disease.** *Mol Neurobiol* 2018, **55:**1977-1987.

56. Ruan Z, Delpech JC, Venkatesan Kalavai S, Van Enoo AA, Hu J, Ikezu S, Ikezu T: **P2RX7 inhibitor suppresses exosome secretion and disease phenotype in P301S tau transgenic mice.** *Mol Neurodegener* 2020, **15:**47.

57. Du Y, Guo T, Hao Y, Li C, Tang L, Li X, Zhang X, Li L, Yao D, Xu X, et al: **PKCdelta serves as a potential biomarker and therapeutic target for microglia-mediated neuroinflammation in Alzheimer's disease.** *Alzheimers Dement* 2024, **20:**5511-5527.

58. Ofengeim D, Mazzitelli S, Ito Y, DeWitt JP, Mifflin L, Zou C, Das S, Adiconis X, Chen H, Zhu H, et al: **RIPK1 mediates a disease-associated microglial response in Alzheimer's disease.** *Proc Natl Acad Sci U S A* 2017, **114:**E8788-E8797.

59. Ponce J, Ulu A, Hanson C, Cameron-Smith E, Bertoni J, Wuebker J, Fisher A, Siu KC, Marmelat V, Adamec J, Bhatti D: **Role of Specialized Pro-resolving Mediators in Reducing Neuroinflammation in Neurodegenerative Disorders.** *Front Aging Neurosci* 2022, **14:**780811.

60. Zhu M, Wang X, Hjorth E, Colas RA, Schroeder L, Granholm AC, Serhan CN, Schultzberg M: **Pro-Resolving Lipid Mediators Improve Neuronal Survival and Increase Abeta42 Phagocytosis.** *Mol Neurobiol* 2016, **53:**2733-2749.

61. Decker C, Sadhu S, Fredman G: **Pro-Resolving Ligands Orchestrate Phagocytosis.** *Front Immunol* 2021, **12:**660865.

62. Li L, Cheng SQ, Sun YQ, Yu JB, Huang XX, Dong YF, Ji J, Zhang XY, Hu G, Sun XL: **Resolvin D1 reprograms energy metabolism to promote microglia to phagocytize neutrophils after ischemic stroke.** *Cell Rep* 2023, **42:**112617.

63. Xu Q, Xu W, Cheng H, Yuan H, Tan X: **Efficacy and mechanism of cGAMP to suppress Alzheimer's disease by elevating TREM2.** *Brain Behav Immun* 2019, **81:**495-508.
